# Supplementary material for: The Safety and Efficacy of Glucosamine and/or Chondroitin in Humans: A Systematic Review
Source: Nutrients. 2025 Jun 24;17(13):2093. doi: 10.3390/nu17132093 (PMC12250884; doi:10.3390/nu17132093)
Supplement: Supplementary file 1 [file nutrients-17-02093-s001.zip › Table S1.pdf]

<u>**Table S1. Overview of Key Characteristics of Glucosamine and/or Chondroitin Studies**</u>

RCT

First Author (Year)<br>Research Design | Geographic Region<br>N of Participants | Length of Study	Use of Glucosamine or Chondroitin	Intervention and Comparator Groups with Dosing	Health Condition	Measures for Efficacy and Safety Outcomes
**Alayat (2017)<br>RCT | Middle East<br>67 | 3 months**	Glucosamine + Chondroitin	Group 1: High-intensity laser therapy (HILT), glucosamine sulfate 500 mg + chondroitin sulfate 400 mg three times daily (GCS), and exercises (HILT + GCS + EX)<br><br>Group 2: GCS + exercises (GCS + EX)<br><br>Group 3: Placebo laser + exercises (PL + EX)	Osteoarthritis	VAS<br>WOMAC<br>Synovial thickness and femoral cartilage thickness on ultrasound
**Alhayek (2023)<br>RCT | Middle East<br>36 | 3 months**	Glucosamine + Chondroitin	Twin-Block appliance + Jointance gel (soluble glucosamine, chondroitin, menthol, ginger, eucalyptus, lavender)<br><br>Comparator: Twin-Block appliance only	Temporomandibular joint dysfunction	Anterior joint space<br>Superior and posterior joint spaces<br>TMJ linear measurements<br>Pain and tension levels.<br>Anterior and posterior glenoid and anterior condylar distances to the PTV reference plain
**Amalraj (2019)<br>RCT | Asia<br>24 | 90 days**	Glucosamine + Chondroitin	Glucosamine 1500 mg and chondroitin 1200 mg daily<br><br>Comparator: Acujoint (Boswellia serrata, Piper nigrum, Kaempferia galanga, and Curcuma longa) 250 mg daily	Osteoarthritis, Joint pain	WOMAC<br>VAS<br>LFI<br>Functional ability score<br>Biomarkers: hsCRP and ESR
**Armagan (2015)<br>RCT | Middle East<br>70 | 6 months**	Glucosamine only	Glucosamine sulfate 1500 mg per day orally<br><br>Comparator: Home exercise program	Osteoarthritis, Joint pain	VAS<br>WOMAC<br>20-minute walking time<br>Joint cartilage thickness
**Babur (2022)<br>RCT | Middle East<br>24 | 4 weeks**	Glucosamine + Chondroitin	Glucosamine 500 mg + chondroitin sulfate sodium 400 mg three times daily + manual therapy and resistance exercise training<br><br>Comparator: Manual therapy and resistance exercise training	Osteoarthritis	VAS<br>KOOS<br>Knee ROM<br>Modified sphygmomanometer test for isometric muscle strength in knee flexion and extension<br>5XRSS<br>Biodex balance system fall risk score<br>In-Body 720
**Basak (2004)<br>RCT | Asia<br>12 | 9 days**	Glucosamine only	1,000 mg (2 x 500 mg given 12 hours apart) dose of timed release glucosamine sulfate (TimeOsamine)<br><br>Comparator: 1500 mg (3 x 500 mg given 8 hours apart) dose of the powder-filled glucosamine sulfate formulation	Other: Bioavailability	Cmax of the powder filled and timed release formulations.<br>Tmax of TimeOsamine.<br>AUC of timeOsamine and powder filled formulation.
**Boeri (2024)<br>RCT | Europe<br>50 | 1 year**	Glucosamine + Chondroitin	Oral preparation (capsule) of HA (100 mg), CS (400 mg), N-acetylglucosamine (200 mg), and vitamin C (80 mg) once daily plus an oral preparation of cranberry, D-	Other: urinary tract infection	Female sexual function index score<br>IPSS

		mannose, propolis extract, turmeric, and Boswellia twice daily<br><br>Comparator: Oral preparation of cranberry, D-mannose, propolis extract, turmeric, and Boswellia twice daily		
**Cahlin (2011)**<br>**RCT | Europe**<br>**59 | 4 years**	Glucosamine only	1200 mg glucosamine sulfate<br><br>Comparator: Placebo	Osteoarthritis, Temporomandibular joint dysfunction	VAS<br>Verbal rating scale (VRS)
**Catanzaro (2013)**<br>**RCT | Europe**<br>**60 | 18 weeks**	Glucosamine + Chondroitin	Group B: Glucosamine 500 mg + chondroitin sulfate 400 mg (frequency/route unspecified)<br><br>Comparator: Group A: LD-1227 (dose/frequency/route unspecified)	Osteoarthritis	VAS<br>LI<br>WOMAC<br>KOOS<br>Biomarkers: IL-6, IL-β, CRP, TNF-sR1 and TNF-sR2
**Chopra (2013)**<br>**RCT | Asia**<br>**440 | 6 months**	Glucosamine only	● Oral glucosamine sulphate 2 g daily, three times a day in equally divided doses<br>●<br>● Comparators:<br>● 2 capsules, three times daily: Shunthi-guduchi SGCG capsule (400 mg) containing Zingiber officinale, Tinospora cordifolia, Phyllanthus emblica and B. serrata.<br>●<br>● 2 capsule, three times daily: Shunthi-guduchi SGC capsule (400 mg) containing the same ingredients as SGCG but without B. serrata extract and a higher quantity of excipients<br>●<br>● Celecoxib 200 mg daily	Osteoarthritis	WOMAC<br>Global assessment (patient and physician)<br>HAQ<br>Biomarkers: urinary CTX-II, serum hyaluronic acid
**Clegg (2006)**<br>**RCT | United States**<br>**1583 | 24 weeks**	Glucosamine + Chondroitin	● 500 mg glucosamine three times daily<br>●<br>● 400 mg of chondroitin sulfate three times daily<br>●<br>● 500 mg glucosamine plus 400 mg chondroitin sulfate three times daily<br>●<br>●<br>● Comparators:<br>● 200 mg of celecoxib daily<br>●<br>● Placebo	Osteoarthritis, Joint pain	Rate of response<br>OMERACT-OARSI response rates
**Cohen (2003)**<br>**RCT | Australia**<br>**63 | 8 weeks**	Glucosamine + Chondroitin	Topical glucosamine sulfate (30 mg/g) chondroitin sulfate (50 mg/g), shark cartilage (140 mg/g), camphor and peppermint oil<br><br>Comparator: Placebo	Osteoarthritis	VAS<br>WOMAC<br>SF-36
**Cömert Kılıç (2021)**<br>**RCT | Middle East**<br>**26 | 12 months**	Glucosamine + Chondroitin	Study group: A single-session arthrocentesis plus intra articular HA injection followed by 3 months of oral GCM supplementation (750 mg glucosamine hydrochloride, 600 mg chondroitin sulfate, and 350 mg	Osteoarthritis, Temporomandibular joint dysfunction	Pain complaints and joint sounds<br>Masticatory efficiency<br>Lateral mandibular motion<br>Maximal interincisal opening

		methylsulfonylmethane, at 2 x 1 dosage daily<br><br>Comparator: Control group: a single-session arthrocentesis plus intra articular HA injection (Hyalgan, 20 mg per 2 ml)		Protrusive mandibular motion<br>VAS<br>CBCT scans of progressions of hard-tissue TMJ structures.
**Crowley (2009)**<br>**RCT | Canada**<br>**52 | 90 days**	Glucosamine + Chondroitin	2 capsules (each containing 375 mg of glucosamine HCl + 300 mg of chondroitin sulfate) in the morning and 2 in the evening for a daily dose of 1500 mg glucosamine and 1200 mg chondroitin<br><br>Comparator: 2 placebo capsules in the morning and 2 undenatured type II collagen (UC-II) in the evening (each containing 20 mg UC-II standardized to 5 mg of bioactive UC-II) for a daily dose of 40 mg UC-II containing 10 mg of bioactive UC-II	Osteoarthritis	WOMAC<br>VAS<br>LI<br>Knee flexion<br>Time to walk 50 meters<br>Time to climb 10 steps<br>Global assessment (patient and physician)
**Czajka (2018)**<br>**RCT | Europe**<br>**120 | 90 Days**	Glucosamine + Chondroitin	● Per 50 mL (1 dose) daily: 2% Glucosamine HCl and 1% Chondroitin Sulphate<br>●<br>● Comparator: Placebo	Skin elasticity<br><br>Joint pain	SkinLab USB Elasticity Module<br>Histological examination<br>Self-perception questionnaires<br>Joint pain/Lysholm scoring questionnaires
**Damlar (2015)**<br>**RCT | Middle East**<br>**31 | 8 weeks**	Glucosamine + Chondroitin	● 1500 mg glucosamine and 1200 mg chondroitin sulphate per day<br>●<br>● Comparator: 50 mg tramadol HCl twice daily	Temporomandibular joint dysfunction	Maximum mouth opening assessment<br>Biomarkers: PGE2, IL-1β, IL-6, TNF-α
**Das (2000)**<br>**RCT | United States**<br>**93 | 6 months**	Glucosamine + Chondroitin	1000 mg FCHG49 glucosamine HCl, 800 mg TRH122 sodium chondroitin sulfate, and 152 mg manganese ascorbate twice daily (Cosamin DS)<br><br>Comparator: Placebo	Osteoarthritis	Lesquene ISK<br>WOMAC<br>Global assessment (patient)
**Eraslan (2015)**<br>**RCT | Middle East**<br>**34 | 8 weeks**	Glucosamine only	1000 mg Glucosamine Sulfate daily<br><br>Comparator: Placebo	Joint Pain	VAS<br>IKDC<br>LYS<br>Peak torque<br>Average power
**Erhan (2012)**<br>**RCT | Middle East**<br>**60 | 4 weeks**	Glucosamine + Chondroitin	Topical glucosamine-chondroitin cream (1500 mg of glucosamine sulfate, 1200 mg of chondroitin sulfate, and aromatic oils)<br><br>Physical therapy and placebo cream massaged around the knee 3 times a day.	Osteoarthritis	WOMAC
**Esfandiari (2017)**<br>**RCT | Middle East**<br>**88 | 3 months**	Glucosamine only	750 mg glucosamine sulfate three times a day<br><br>Comparator: Placebo	Other: intraocular pressure	Mean IOP<br>Ocular response analyzer parameters
**Filipović (2022)**<br>**RCT | Europe**<br>**111 | 12 months**	Glucosamine only	Group 1: 1500 mg/day crystalline glucosamine sulfate (CGS) powder for oral solution once daily (with daily intake for the first 6 and last 3 months, with a 3-month pause in months 7 to 9)<br><br>Comparator: Ibuprofen 400 mg tablets taken three or four times a day, or diclofenac sodium, one 75 mg capsule a day (with continuous use for the first 15 days,	Osteoarthritis	WOMAC<br>LI<br>Radiological findings of JSW

		then only when pain occurred, which was limited to 5 days)		
**Fransen (2015)**<br>**RCT | Australia**<br>**605 | 2 years**	Glucosamine + Chondroitin	Two capsules of glucosamine sulfate 753 mg once daily<br><br>Two capsules of chondroitin sulfate 400 mg once daily<br><br>One glucosamine sulfate 753 mg and one chondroitin sulfate 400 mg capsule daily<br><br>Comparator: Placebo	Osteoarthritis	Medial tibio-femoral joint space narrowing<br>WOMAC<br>SF-12<br>50-foot walk time
**Giordano (2009)**<br>**RCT | Europe**<br>**60 | 24 weeks**	Glucosamine only	1500 mg glucosamine daily<br><br>Comparator: Placebo	Osteoarthritis	VAS<br>WOMAC<br>NSAID consumption
**Gruenwald (2009)**<br>**RCT | Europe**<br>**177 | 26 weeks**	Glucosamine only	Group A: 3 capsules daily; each capsule contained 500 mg glucosamine sulfate 2 KCl; 444 mg fish oil; 200 mg omega-3-fatty acids; 120 µg vitamin A; 0.75 µg vitamin D; 1.5 mg vitamin E<br><br>Comparator: Group B: 3 capsules daily; each capsule contained 500 mg glucosamine sulfate 2 KCl; 444 mg mixture of palm oil [70%], rapeseed oil [15%], and sunflower oil [15%]; 120 µg vitamin A; 0.75 µg vitamin D; 1.5 mg vitamin E	Osteoarthritis	WOMAC<br>OA symptom assessment
**Herrero-Beaumont (2007)**<br>**RCT | Europe**<br>**318 | 6 months**	Glucosamine only	1,500 mg glucosamine sulfate once daily<br><br>Comparators:<br>Acetaminophen 1 g tablets 3 times a day<br><br>Placebo	Osteoarthritis	LI
**Hochberg (2008)**<br>**RCT | United States**<br>**1583 | 24 weeks**	Glucosamine + Chondroitin	5 groups:<br>Glucosamine 500 mg three times daily<br><br>Chondroitin sulfate 400 mg three times daily<br><br>Combination glucosamine + chondroitin sulfate in the same dosages<br><br>Celecoxib 200 mg daily<br><br>Placebo	Osteoarthritis	WOMAC<br>VAS<br>LFI<br>Knee flexion<br>Biomarkers: COMP, CRP, IL-6. MMP-3
**Hochberg (2016)**<br>**RCT | Europe**<br>**606 | 6 months**	Glucosamine + Chondroitin	400 mg chondroitin sulfate plus 500 mg glucosamine hydrochloride three times a day<br><br>Comparator: Celecoxib 200 mg daily + placebo	Osteoarthritis	WOMAC<br>VAS<br>Presence of joint swelling/effusion<br>Rescue medication use<br>OMERACT-OARSI<br>EUroQoL-5D
**Kanzaki (2012)**<br>**RCT | Asia**<br>**40 | 16 weeks**	Glucosamine + Chondroitin	1200 mg glucosamine hydrochloride, 60 mg chondroitin sulfate, and 45 mg quercetin glycosides once daily (GCQ)	Osteoarthritis	JOA<br>VAS<br>Biomarkers: CTX-II and CPII

		Comparator: Placebo		
**Kanzaki (2015)**<br>**RCT | Asia**<br>**100 | 16 weeks**	Glucosamine + Chondroitin	Once daily GCQID tablets containing 1,200 mg glucosamine hydrochloride, 60 mg of chondroitin sulfate, 45 mg of type II collagen peptides, 90 mg of quercetin glycosides, 10 mg of imidazole peptides, and 5 mcg of vitamin D<br><br>Comparator: Placebo	Osteoarthritis, Joint pain	Efficacy assessment<br>Knee-joint functions<br>Locomotor functions<br>Knee-extensor strength<br>Walking speed
**Kawasaki (2008)**<br>**RCT | Asia**<br>**142 | 18 months**	Glucosamine only	Glucosamine 1500 mg daily<br><br>Comparators:<br>Risedronate 2.5 mg/day<br><br>No supplement	Osteoarthritis	Range of motion<br>WOMAC<br>VAS<br>JOA score
**Khanna (2020)**<br>**RCT | Asia**<br>**80 | 84 days**	Glucosamine + Chondroitin	● 415 mg chondroitin sulfate (CHN) and 500 mg glucosamine hydrochloride (GLN) twice a day<br>●<br>● Comparator: 400 mg curcumagalactomannosides (CGM) and 500 mg glucosamine hydrochloride (GLN) twice a day	Osteoarthritis, Joint pain	Treadmill walking score<br>VAS<br>KPS score<br>WOMAC<br>Physical function
**Kongtharvonskul (2016)**<br>**RCT | Asia**<br>**148 | 6 months**	Glucosamine only	1500 mg crystalline glucosamine sulfate (pCGS) and 50 mg diacerein daily<br><br>Comparator: 1500 mg crystalline glucosamine sulfate (pCGS) and placebo	Osteoarthritis	VAS<br>WOMAC
**Kwoh (2014)**<br>**RCT | United States**<br>**201 | 24 Weeks**	Glucosamine only	1500 mg glucosamine hydrochloride<br><br>Comparator: Placebo	Joint Pain	WORMS<br>WOMAC 3.1<br>BML<br>Biomarkers: Urinary creatinine and urinary CTX-II
**Leffler (1999)**<br>**RCT | United States**<br>**34 | 16 weeks**	Glucosamine + Chondroitin	Oral Cosamin [glucosamine HCl (1500 mg/day), chondroitin sulfate (1200 mg/day), and manganese ascorbate (228 mg/day) in 3 divided doses]. After 8 weeks, patients crossed over to the regimen not followed previously.<br><br>Comparator: Placebo	Other: Degenerative joint disease (DJD)	VAS<br>Patient assessment of treatment results<br>Physical examination scores<br>Disability score of Lequesne and Roland<br>Physical assessment of severity<br>Running time.
**Lomonte (2018)**<br>**RCT | South America/Latin America**<br>**100 | 12 weeks**	Glucosamine + Chondroitin	3 capsules a day of 500 mg glucosamine sulfate and 400 mg chondroitin sulfate (Eurofarma Laboratórios)<br><br>Comparator: Cosamin DS 3 capsules a day (containing 500 mg glucosamine hydrochloride and 400 mg chondroitin sulfate)	Osteoarthritis	Analgesic efficacy<br>VAS<br>Joint pain and swelling<br>Use of rescue medication
**Lomonte (2021)**<br>**RCT | South America/Latin America**<br>**627 | 24 weeks**	Glucosamine + Chondroitin	● Ártico (1500 mg glucosamine sulfate and 1200 mg bovine chondroitin sulfate) one sachet daily<br>●<br>● Comparator: Condroflex (1500 mg glucosamine sulfate and 1200 mg bovine	Osteoarthritis	WOMAC<br>VAS<br>SF-12<br>OMERACT-OARSI<br>Rescue medication use

		chondroitin sulfate) one sachet daily (reference product/RP)		
**Lubis (2017)**<br>**RCT | Asia**<br>**147 | 12 weeks**	Glucosamine + Chondroitin	● Glucosamine-chondroitin sulfate (GC) group: 1500 mg of glucosamine + 1200 mg of chondroitin sulfate + 500 mg of saccharumlactis<br>●<br>● Glucosamine-chondroitin sulfate-MSM (GCM) group: 1500 mg of glucosamine + 1200 mg of chondroitin sulfate + 500 mg of MSM<br>●<br>● Comparator: Placebo	Osteoarthritis	VAS<br>WOMAC
**Lugo (2016)**<br>**RCT | Asia**<br>**191 | 180 days**	Glucosamine + Chondroitin	● GC group: 1500 mg glucosamine and 1200 mg chondroitin<br>●<br>● Comparators:<br>● UC=II group: 40 mg undenatured type II collagen<br>●<br>● Placebo	Osteoarthritis	WOMAC<br>LFI<br>VAS
**Luo (2022)**<br>**RCT | Asia**<br>**101 | 12 weeks**	Glucosamine + Chondroitin	● G + C group: 1500 mg glucosamine and 1200 mg chondroitin daily in two divided doses<br>●<br>● Comparators:<br>● TII group: Type II collagen 40 mg daily<br>●<br>● Placebo	Osteoarthritis	WOMAC<br>Health-related quality of life questionnaire
**Magrans-Courtney (2011)**<br>**RCT | United States**<br>**30 | 14 weeks**	Glucosamine + Chondroitin	● 1,500 mg/d of glucosamine, 1,200<br>● mg/d of chondroitin sulfate, 900 mg/d of methylsulfonylmethane (MSM), 120 mg/d of niacin, 120 mg/d of sodium, 45 mg/d of zinc, 300 mg/d of boswellia serrata extract, 180 mg/d of white willow bark extract, and 15 mg/d of rutin powder (3 caplets in the morning and 2 caplets in the evening 30 minutes before a meal) + exercise<br>●<br>● Comparator: Placebo + exercise	Osteoarthritis	SF-36<br>VAS<br>WOMAC<br>Body composition and resting energy expenditure<br>Knee range of motion/circumference<br>Exercise performance<br>Isokinetic knee extension and flexion<br>Balance and functional capacity<br>Biomarkers: CRP, IL-6 TNF-α, cortisol, leptin, lipids, glucose, insulin resistance (HOMAIR), and renal and liver function proteins/enzymes
**Mazières (2007)**<br>**RCT | Europe**<br>**307 | 32 weeks**	Chondroitin only	500 mg chondroitin sulfate twice a day<br><br>Comparator: placebo	Osteoarthritis, Joint pain	VAS<br>LFI<br>OMERACT-OARSI<br>SF-12
**Messier (2007)**<br>**RCT | United States**<br>**72 | 12 months**	Glucosamine + Chondroitin	1500/1200 mg of GH/CS daily (given as a once or three times per day regimen) for 6 months<br><br>Comparator: Placebo	Osteoarthritis	WOMAC<br>6-minute walk<br>MMSE<br>Balance<br>Knee strength
**Michel (2005)**<br>**RCT | Europe**	Chondroitin only	800 mg tablet of chondroitins 4 and 6 sulfate daily for two years (contained magnesium stearate,	Osteoarthritis	Minimum and mean JSW<br>WOMAC

**300 | 2 years**		hydroxypropyl methylcellulose, polyethylene glycol, and titanium dioxide.<br><br>Comparator: Placebo		
**Minoretti (2024)**<br>**RCT | Europe**<br>**51 | 4 weeks**	Glucosamine + Chondroitin	Glc + CS group: Cartijoint Forte (1 tablet/day) containing Glc (415 mg), CS (400 mg), and curcuminoids (50 mg)<br><br>Comparators:<br>HA group: Syalox 300 Plus (1 tablet/day) containing HA (300 mg) and Boswellia serrata extract (100 mg)<br><br>No supplements (watchful waiting)	Osteoarthritis	VAS<br>WOMAC<br>Biomarkers: serum adropin
**Monfort (2017)**<br>**RCT | Europe**<br>**49 | 4 months**	Chondroitin only	Chondroitin sulfate (CS) 800 mg once daily<br><br>Comparator: Placebo	Osteoarthritis	fMRI of patella pain<br>Interline pressure test
**Morita (2018)**<br>**RCT | Asia**<br>**73 | 12 months**	Chondroitin only	High dose: 1560 mg daily chondroitin sulfate (taken as 2 active tablets three times daily)<br><br>Comparator: Low dose: 260 mg daily chondroitin sulfate (taken as 1 active and 1 placebo tablet in the morning and two placebo tablets another two times a day)	Osteoarthritis	LI<br>VAS<br>Biomarkers: COMP and hyaluronic acid levels
**Nakamura (2007)**<br>**RCT | Asia**<br>**51 | 12 weeks**	Glucosamine only	Glucosamine hydrochloride 500 mg three times daily (total daily dose of 1500 mg) + conventional medication<br><br>Comparator: Placebo + conventional medication	Rheumatoid arthritis	Swollen joint count and painful joint count<br>Face scale and VAS pain score<br>ACR20 improvement<br>Global assessment (patient and physician)<br>Biomarkers: ESR, CRP, MMP-3 levels
**Nakasone (2011)**<br>**RCT | Asia**<br>**32 | 9 months**	Glucosamine + Chondroitin	1,200 mg of glucosamine hydrochloride, 200 mg of shark cartilage extract (with approximately 60 mg of chondroitin sulfate), 300 mg of MSM, 105 mg of guava leaf extract, 5.6 μg of vitamin D, and 7.35 mg of vitamin B at a daily dose of 7 tablets<br><br>Comparator: Placebo	Osteoarthritis	JKOM<br>VAS<br>Biomarkers: C2C, synovitis hyaluronan
**Nash (2018)**<br>**RCT | Europe**<br>**122 | 6 months**	Glucosamine + Chondroitin	2700 mg (1500 mg glucosamine hydrochloride and 1200 mg of chondroitin sulfate), divided twice daily<br><br>Comparator: Q-Actin [aqueous extract of Cucumis sativus (cucumber; CSE)] 10 mg twice daily	Osteoarthritis	WOMAC<br>VAS<br>LFI
**Navarro (2015)**<br>**RCT | United States**<br>**18 | 9 months**	Glucosamine + Chondroitin	1500 mg/d FCHG49 glucosamine hydrochloride (GHCl) + 1200 mg/d TRH122 sodium chondroitin sulfate (CS) taken as 3 capsules daily each capsule containing 500 mg GHCl and 400 CS<br><br>Comparator: Placebo	Other: Inflammation and oxidative stress	Mean CRP concentrations<br>KEGG pathway analysis<br>GO pathway analysis<br>Bonferroni correction
**Navarro (2019)**<br>**RCT | United States**<br>**10 | 4 weeks**	Glucosamine + Chondroitin	Cosamin DS: 1500 mg per day glucosamine hydrochloride + 1200 mg per day sodium chondroitin sulfate taken as 3 capsules daily (each capsule containing 500 mg glucosamine hydrochloride and 400 mg chondroitin sulfate)	Other: None	Fecal microbial alpha and beta diversity

		Comparator: Placebo		
**Navarro (2020)**<br>**RCT | Europe**<br>**606 | 6 Months**	Glucosamine + Chondroitin	500 mg Glucosamine Hydrochloride and 400 mg Chondroitin Sulfate three times a day<br><br>Comparator: 200 mg Celecoxib once daily	Joint Pain	WOMAC<br>Biomarkers: Ccl20, Ccsp-1, Ceacam1, Clorf38, CRP, Csf3, Cxcl12, Hbegf, IL-6, IL13, Itga5, Marveld2, Nckipsd, Npr3, Sfrs12, Spp1/Osteopontin, Thbs4, Tnfrsf17, Tsg101, Wnt16
**Nguyen (2001)**<br>**RCT | United States**<br>**45 | 12 weeks**	Glucosamine + Chondroitin	CS-GH three tablets twice daily for three months (each tablet contained GH 250 mg for a total of 1500 mg per day and CS 200 mg for a total of 1200 mg per day)<br><br>Comparator: Placebo	Temporomandibular joint dysfunction	VAS<br>Mc-Gill pain questionnaire<br>Mood and functioning score<br>TMJ palpation<br>Myofascial pain<br>Jaw range of motion (ROM)
**Nieman (2013)**<br>**RCT | United States**<br>**108 | 8 Weeks**	Glucosamine only	Glucosamine sulfate 1500 mg in three divided doses<br><br>Comparator: Placebo	Joint Pain	WOMAC<br>SF-36<br>12-point likert visual scale (12-VS)<br>6-minute walk test<br>Biomarkers: CRP, IL-6, TNFα, IL-8, IL-10
**Pavelká (2002)**<br>**RCT | Europe**<br>**202 | 3 years**	Glucosamine only	Oral glucosamine sulfate 1500 mg once a day<br><br>Comparator: Placebo	Osteoarthritis	Progressive joint space narrowing<br>LI<br>WOMAC
**Pelletier (2016)**<br>**RCT | Canada**<br>**194 | 24 months**	Chondroitin only	CS 1200 mg (three 400 mg capsules in the morning)<br><br>Comparator: Celecoxib 200 mg (one 200 mg capsule + two placebo capsules in the morning)	Osteoarthritis	Cartilage volume<br>BML grade<br>Synovitis severity<br>Synovial fluid volume<br>VAS<br>WOMAC<br>SF-36
**Petersen (2011)**<br>**RCT | Europe**<br>**36 | 12 weeks**	Glucosamine only	Glucosamine sulfate tablets of 500 mg 3 times daily + quadriceps muscle training<br><br>Comparators<br>Ibuprofen 600 mg twice daily + quadriceps muscle training<br><br>Placebo + quadriceps muscle training	Osteoarthritis	Muscle cross-sectional area (CSA), maximal isometric strength, maximal eccentric muscle strength and eccentric muscle work.
**Provenza (2015)**<br>**RCT | South America/Latin America**<br>**922 | 22 months**	Glucosamine + Chondroitin	Groups I and II:<br>500 mg glucosamine sulfate/400 mg chondroitin sulfate either as capsules three times daily or as a sachet preparation once daily, respectively<br><br>Group III: Cosamin DS (500 mg glucosamine hydrochloride/400 mg chondroitin sulfate) three times daily	Osteoarthritis	VAS<br>LI<br>Patient-reported pain in affected knee<br>Global assessment (patient and physician)<br>Acetaminophen consumption
**Puente (2017)**<br>**RCT | South America/Latin America**<br>**60 | 12 weeks**	Glucosamine + Chondroitin	Glucosamine plus chondroitin sulphate (GS/CS) 375/300 mg tablet once daily<br><br>Comparator: D-002 (beeswax alcohols) 50 mg tablet once daily	Osteoarthritis	WOMAC<br>VAS<br>Rescue medication consumption
**Railhac (2012)**	Chondroitin only	Structum (chondroitin sulfate 500 mg) oral twice daily	Osteoarthritis	VAS<br>LI

**RCT | Europe**<br>**43 | 48 weeks**		Comparator: Placebo		Cartilage volume and change in osteoarticular lesions
**Reginster (2001)**<br>**RCT | Europe**<br>**212 | 3 years**	Glucosamine only	1500 mg glucosamine sulphate once daily<br><br>Comparator: placebo	Osteoarthritis	Joint space narrowing.<br>WOMAC
**Rindone (2000)**<br>**RCT | United States**<br>**98 | 2 months**	Glucosamine only	Glucosamine 500 mg three times daily<br><br>Comparator: Placebo	Osteoarthritis	VAS
**Roman-Blas (2017)**<br>**RCT | Europe**<br>**164 | 6 months**	Glucosamine + Chondroitin	A sachet of a formulation containing 1,200 mg chondroitin sulfate plus 1,500 mg crystalline glucosamine sulfate once a day<br><br>Comparator: Placebo	Osteoarthritis, Joint pain	VAS<br>WOMAC<br>OMERACT-OARSI
**Rondanelli (2019)**<br>**RCT | Europe**<br>**60 | 12 weeks**	Chondroitin only	600 mg of Mythocondro (chondroitin sulfate) daily for 12 weeks<br><br>Comparator: Placebo	Osteoarthritis	TLKS<br>WOMAC<br>VAS<br>Biomarkers: CRP and ESR
**Rondanelli (2020)**<br>**RCT | Europe**<br>**48 | 8 weeks**	Chondroitin only	600 mg of Mythocondro (non animal chondroitin sulfate) daily<br><br>Comparator: Placebo	Osteoarthritis	WOMAC<br>BML<br>CRP<br>Total cholesterol and homeostasis model assessment (HOMA)
**Sawitzke (2008)**<br>**RCT | United States**<br>**357 | 24 months**	Glucosamine + Chondroitin	5 groups:<br>Glucosamine hydrochloride (G) 500 mg 3 times daily<br><br>Sodium chondroitin sulfate (CS) 400 mg 3 times daily<br><br>Combination of G+CS<br><br>Celecoxib 200 mg daily<br><br>Placebo	Osteoarthritis	JSW loss<br>JSW progression (> 0.48 mm)
**Scroggie (2003)**<br>**RCT | United States**<br>**38 | 90 days**	Glucosamine + Chondroitin	Cosamin DS (500 mg of glucosamine hydrochloride, 400 mg of chondroitin sulfate, 5 mg of manganese, and 66 mg of ascorbic acid) for a total dose of 1500 mg per day of glucosamine hydrochloride + 1200 mg per day of chondroitin sulfate<br><br>Comparator: Placebo	Other: Type 2 diabetes	Hemoglobin A1c concentrations
**Sevimli (2020)**<br>**RCT | Middle East**<br>**202 | 6 months**	Glucosamine + Chondroitin	Glucosamine + chondroitin sulfate (dose not specified)<br><br>Comparator: None	Osteoarthritis	WOMAC
**Sterzi (2016)**<br>**RCT | Europe**<br>**53 | 12 weeks**	Glucosamine + Chondroitin	• CartiJoint Forte 2 tablets per day containing 400 mg chondroitin sulfate (CS), 500 mg glucosamine hydrochloride (GH) and 50 mg BioCurcumin BCM-95 + physical therapy<br>•<br>• Comparator: Placebo + physical therapy	Osteoarthritis	VAS<br>WOMAC<br>LI<br>Knee range of motion<br>Biomarkers: CRP and ESR
**Thomas (2021)**	Glucosamine + Chondroitin	Chondroitin sulphate (CHN) 415 mg and glucosamine	Osteoarthritis	Walking performance

RCT | Asia<br>84 | 6 weeks		hydrochloride (GLN) 500 mg twice daily<br><br>Comparator: Low-dose curcumagalactomannosides (CGM) 400 mg once daily		VAS<br>KPS<br>WOMAC<br>Biomarkers: IL-1-β, IL-6, sVCAM, hsCRP
**Tío (2017)**<br>**RCT | Europe**<br>**70 | 6 months**	Chondroitin only	CS 800 mg orally once daily<br><br>Comparator: Acetaminophen 3 g orally daily	Osteoarthritis	Synovitis presence
**Truong (2019)**<br>**RCT | Asia**<br>**100 | 12 weeks**	Glucosamine only	Glucosamine 1500 mg (three 500 mg capsules) once daily (control group)<br><br>Comparators:<br>Group (A): Per capsule: 16.7 mg of Polycan, 250 mg of glucosamine; three capsules once a daily<br><br>Group (B): Per capsule: 16.7 mg of Polycan, 500 mg of glucosamine; three capsules once a daily	Osteoarthritis	WOMAC
**Tsuji (2016)**<br>**RCT | Asia**<br>**50 | 24 Weeks**	Glucosamine + Chondroitin	100 mg of N-acetyl glucosamine and 180 mg of chondroitin sulfate daily<br><br>Comparator: Placebo	Joint Pain	JKOM<br>VAS<br>PASE<br>Timed up and go test<br>6-minute walk test
**Uebelhart (2004)**<br>**RCT | Europe**<br>**120 | 1 year**	Chondroitin only	Condrosulf sachets (800 mg chondroitin sulfate) daily<br><br>Comparator: placebo	Osteoarthritis	Lequesne's algo-functional index (AFI)<br>VAS<br>Walking time<br>Global judgment (patient and physician)<br>Paracetamol consumption<br>Radiological progression and JSW
**Usha (2004)**<br>**RCT | Asia**<br>**118 | 12 weeks**	Glucosamine only	Group 1: Glucosamine (Glu) 500mg three times daily<br>Group 2: Methylsulfonylmethane (MSM) 500mg three times daily<br>Group 3: Glu 500 mg and MSM 500 mg three times daily<br><br>Comparator: Placebo	Osteoarthritis	LI<br>VAS<br>Joint mobility index<br>Mean pain index<br>Mean swelling index<br>Global assessment (patient and physician)
**Velickovic (2023)**<br>**RCT | Europe**<br>**120 | 6 months**	Glucosamine + Chondroitin	First experimental group: 1 capsule of the investigational product (IP) and one capsule of placebo twice daily (375 mg of GS, 300 mg of naCS, and 100 mg of SAMe)<br><br>Second experimental group: 2 capsules of IP twice daily (750 mg of GS, 600 mg of naCS, and 200 mg of SAMe)<br><br>Comparator: Placebo	Osteoarthritis	Articular cartilage thickness<br>VAS,<br>TLKS<br>WOMAC<br>SF -36
**Vicenzino (2019)**<br>**RCT | Australia**<br>**114 | 2 weeks**	Glucosamine + Chondroitin	Knee guard device containing 1 g gel of glucosamine sulphate 10 mg, chondroitin sulphate 2.5 mg, hyaluronic acid 2.5 mg and menthol 40 mg per gram 3-5 hours per day<br><br>Comparator: Diclofenac sodium (1%) gel 4 g applied 4 times daily	Other: Knee injury	Multiple linear regression analyses<br>KOOS-F and AFS response

**Wang (2021)**<br>**RCT | Asia**<br>**47 | 8 weeks**	Glucosamine + Chondroitin	A+HA group: 20 mL oral solution containing a mixture of 50 mg hyaluronic acid (HA), 750 mg glucosamine, and 250 mg chondroitin once daily in the morning<br>Comparator: Placebo	Osteoarthritis	WOMAC<br>SF-36
**Wang (2021)**<br>**RCT | Asia**<br>**80 | 8 weeks**	Glucosamine + Chondroitin	20 mL supplement mixture (50 mg hyaluronan + 750 mg glucosamine + 250 mg chondroitin) once daily<br>Comparator: Placebo	Osteoarthritis	KOOS<br>WOMAC<br>SF-36<br>CPSQI
**Wildi (2011)**<br>**RCT | Canada**<br>**69| 1 year**	Chondroitin only	Chondroitin sulfate 800mg (two capsules of 400 mg each) once daily for the first 6 months followed by 6 months of 800 mg of chondroitin sulfate once daily.<br>Comparator: Placebo once daily for the first 6 months followed by 6 months of 800mg chondroitin sulfate once daily	Osteoarthritis	Cartilage volume<br>BML score<br>WOMAC
**Wilkens (2010)**<br>**RCT | Europe**<br>**250 | 1 year**	Glucosamine only	1500 mg of oral glucosamine daily<br>Comparator: Placebo	Osteoarthritis; Other: Chronic low back pain	Mean RMDQ scores<br>LBP during activity<br>Quality of life EQ-5D
**Xia (2016)**<br>**RCT | Asia**<br>**150 | 8 weeks**	Glucosamine only	GS three times daily (dose unspecified)<br>Intra-articular injection hyaluronic acid (IAHA) 25 mg weekly<br>Comparator: Placebo three times daily	KBD	WOMAC
**Yue (2012)**<br>**RCT | Asia**<br>**251 | 6 months**	Glucosamine + Chondroitin	600 mg chondroitin sulfate twice daily<br>480 mg glucosamine hydrochloride three times daily<br>Combination of 480 mg glucosamine three times daily plus 600 mg chondroitin sulfate twice daily<br>Comparator: Placebo	KBD	WOMAC
**Zegels (2013)**<br>**RCT | Europe**<br>**353 | 3 months**	Chondroitin only	Group 1 (CS 1200): one oral gel sachet of chondroitin sulfate 1200 mg/day & one oral placebo capsule three times a day<br>Group 2 (CS 3*400): one oral placebo gel sachet/day & one oral capsule of CS 400 mg three times a day<br>Comparator: Placebo (Group 3)	Osteoarthritis	LI<br>VAS
**Zenk (2002)**<br>**RCT | United States**<br>**42 | 6 weeks**	Glucosamine only	Glucosamine sulfate 500 mg three times daily (Group 2)<br>Comparators:<br>Milk protein concentrate (MPC) 2000 mg twice daily (Group 1)<br>Placebo (Group 3)	Osteoarthritis	WOMAC OA index scores

**Zhang (2010)**<br>**RCT | Asia**<br>**80 | 8 months**	Glucosamine + Chondroitin	800 mg of chondroitin sulfate and glucosamine twice daily for 8 months<br><br>Comparator: Placebo	KBD	Mean joint space
**Zhang (2021)**<br>**RCT | Asia**<br>**128 | 12 weeks**	Glucosamine only	Combination group (ComG): Glucosamine hydrochloride (480mg) once every 12 hours + celecoxib<br><br>Comparator: Celecoxib tablets (200 mg), once every 24 hours for 4 weeks	Osteoarthritis	VAS<br>LI<br>Biomarkers: IgM rheumatoid factor, albumin/globulin, ESR, TNF-α, IL-6, hs-CRP levels

Scales: Western Ontario and McMaster Universities Arthritis Index (WOMAC), Short Form 12 (SF-12), Short Form 36 (SF-36), Whole-Organ MRI Score (WORMS), Japanese Knee Osteoarthritis Measure (JKOM), Visual Analog Scale (VAS), International Knee Documentation Committee Subjective Form (IKDC), Lysholm Knee Scoring Form (LYS), Physical Activity Scale for the Elderly (PASE), Lequesne index (LI), Lequesne Functional Index (LFI), Health Assessment Questionnaire (HAQ), Tegner Lysholm Knee Score (TLKS), Japan Orthopaedic Association (JOA), Knee disability and Osteoarthritis Outcome Score (KOOS), Karnofsky Performance Scale (KPS), Mini Mental State Examination (MMSE), Lequesne index of severity of osteoarthritis of the knee (ISK), Outcome Measures in Rheumatology Clinical Trials and Osteoarthritis Research Society International (OMERACT-OARSI), 5 repetition sit to stand test (5XRSS), Knee range of motion in flexion and extension (ROM), Chinese version of Pittsburgh Sleep Quality Index (CPSQI), EuroQol 5 Dimension Questionnaire (EuroQoL-5D)
Other abbreviations: C-reactive protein (CRP), high-sensitivity C-reactive protein (hsCRP), interleukin (IL), tumor necrosis factor alpha (TNFα), C-terminal crosslinking telopeptide of type II collagen (CTX-II), erythrocyte sedimentation rate (ESR), bone marrow lesion (BML), joint space width (JSW), cartilage oligomeric matrix protein (COMP), matrix metalloproteinase (MMP), homeostatic model assessment of insulin resistance ($HOMA_{IR}$), cartilage type II collagen degradation (C2C), C-propeptide of type II procollagen (CPII), soluble vascular cell adhesion molecule-1 (sVCAM)

Cohort

First Author (Year)<br>Study Design | Location<br>N of Patients | Study Length	Glucosamine and/or Chondroitin	Intervention (with Dose) and Comparator	Condition	Measures Used
**Bell (2012)**<br>**Cohort | United States**<br>**77,510 | Questionnaire over usage in 10 years prior to baseline**	Glucosamine + Chondroitin	Glucosamine and chondroitin	Osteoarthritis, Joint Pain, Mortality	Mortality rate
**Bhimani (2023)**<br>**Cohort | United States**<br>**38,021 | 17 years**	Glucosamine + Chondroitin	Glucosamine and chondroitin	Other: All cause mortality	Death rates
**Cho (2019)**<br>**Cohort | Asia**<br>**212 | Mean 369.1 days**	Chondroitin only	SYSADOA: diacerein, avocado soybean unsaponifiables, chondroitin, herbal drugs combining herbal extracts including JOINS (a mixed extract of three herbs: Clematis mandshurica, Prunella vulgaris, and Trichosanthes kirilowii); LAYLA (an ethanol extract prepared from 12 plant sources: Chaenomelis fructus, Achyranthis radix, Acanthopanax cortex, Cinnamomi cortex, Gentianae macrophyllae radix, Clematidis radix, Angelica gigantis radix, Cnidii rhizoma, Gastrodiae rhizoma, Carthami flos, Saposhnikoviae radix, and Dipsaci radix); and SHINBARO (a purified extract from a mixture of 6 oriental herbs: Ledebouriellae radix, Achyranthis radix, Acanthopanacis cortex, Cibotii rhizoma, Glycine semen, and Eucommiae cortex)	Osteoarthritis	Frequency of intra-articular injection<br>Impact on discontinuation of NSAID use<br>KL Grade

		Comparator: SYSADOA non-users		
**Cho (2023)**<br>**Cohort | Asia**<br>**1,201,930 | N/A**	Glucosamine + Chondroitin	Glucosamine, chondroitin, vitamin/mineral supplement intake	Other: Kidney Function (eGFR)	eGFR
**Hotaling (2011)**<br>**Cohort | United States**<br>**77,050 | 6 years**	Glucosamine + Chondroitin	Supplemental vitamins (multivitamins, beta-carotene, retinol, folic acid, vitamins B1, B3, B6, B12, C, D and E), supplemental minerals (calcium, iron, magnesium, zinc, and selenium), and anti-inflammatory supplements (glucosamine, chondroitin, saw-palmetto, ginko-biloba, fish oil and garlic)<br><br>Comparator: supplement non-users	Other: Urothelial cell carcinoma of the bladder	Incidence of urothelial carcinoma
**Kantor (2016)**<br>**Cohort | United States**<br>**96,400 | 9 years**	Glucosamine + Chondroitin	Regular use of glucosamine and chondroitin	Other: Colorectal cancer	Incidence of colorectal cancer
**King (2020)**<br>**Cohort | United States**<br>**16,686 | 12 years with a mortality follow-up 5 years later**	Glucosamine + Chondroitin	Regular consumption of glucosamine and chondroitin<br><br>Comparator: N/A	Other: All-cause mortality, cardiovascular mortality	Mortality status
**Li (2023)**<br>**Cohort | Asia**<br>**450,207 | 16 years**	Glucosamine only	Regular use of glucosamine	Other: Cancer	Incidence of cancer
**Lila (2023)**<br>**Cohort | Europe**<br>**1102 | 54-64 weeks**	Glucosamine only	Glucosamine 500 mg and chondroitin sulfate 400 mg combination capsule three times a day for three weeks, followed by a reduced dosage of two capsules daily<br><br>Comparator: N/A	Osteoarthritis	KOOS<br>HOOS<br>5-point satisfaction scale
**Ma (2019)**<br>**Cohort | Europe**<br>**466,039 | 7 year mean follow up**	Glucosamine only	Glucosamine users<br><br>Comparator: Glucosamine non-users	Other: CVD events (CVD death, coronary heart disease, stroke)	Incidence of CVD events
**Pocobelli (2010)**<br>**Cohort | United States**<br>**77,050 | 5 years**	Glucosamine + Chondroitin	Vitamin supplements, mineral supplements, and nonvitamin-nonmineral supplements<br><br>Comparator: N/A	Other: Total mortality	Mortality
**Raynauld (2016)**<br>**Cohort | Canada**<br>**429 | 6 years**	Glucosamine + Chondroitin	Exposed to glucosamine and/or chondroitin sulfate<br><br>Comparator: Not exposed	Osteoarthritis	JSW<br>Cartilage volume loss<br>WOMAC<br>KL grade
**Roubille (2015)**<br>**Cohort | Canada**	Glucosamine + Chondroitin	Stratified design: +/- Analgesics/NSAIDs, presence or absence of medial meniscus extrusion, and +/-	Osteoarthritis	JSW<br>Cartilage volume loss

**600 | 24 months**		glucosamine and chondroitin		KL grade
**Rovati (2016)**<br>**Cohort | Europe**<br>**6451 | 24 months**	Glucosamine + Chondroitin	SYSADOAs: glucosamine sulfate 1500 mg, glucosamine hydrochloride 1250 mg once daily, chondroitin sulfate 400 mg three times daily, diacerein 50 mg twice daily, or avocado soybean unsaponifiables 300 mg once daily<br><br>Comparator: no SYSADOAs	Osteoarthritis	NSAID consumption
**Yang (2015)**<br>**Cohort | United States**<br>**1,625 | 4 years**	Glucosamine + Chondroitin	Exposure to glucosamine/chondroitin<br><br>Comparator: No exposure	Osteoarthritis	WOMAC<br>JSW<br>SF-12
**Yu (2022)**<br>**Cohort | Asia**<br>**685,778 | Mean 6.13 years**	Glucosamine only	Glucosamine users<br><br>Comparator: Glucosamine non-users	Osteoarthritis	New onset of CVD events, CHD, and stroke
**Zheng (2023)**<br>**Cohort | Europe**<br>**494,814 | 8.9 years mean follow up**	Glucosamine only	Glucosamine users<br><br>Comparator: Glucosamine non-users<br><br>Covariates: antihypertensive drugs, insulin treatment, statin, opioids, aspirin, and other non-steroidal anti-inflammatory drugs (NSAIDs), chondroitin, dietary supplements for minerals, vitamins, and other nutrients (fish oil, calcium, iron, zinc, and selenium)	Other: Dementia	Incidence of dementia
**Zheng (2023)**<br>**Cohort | United States**<br>**412,136 | 9 year mean follow up**	Glucosamine + Chondroitin	Glucosamine use<br><br>Comparator: glucosamine non-use	Other: Heart failure	Incidence of heart failure
**Zhou (2023)**<br>**Cohort | Asia**<br>**214,945 | 12 years mean follow up**	Glucosamine only	Exposure to glucosamine<br><br>Comparator: No exposure to glucosamine	Other: Dementia	Incidence of dementia

Scales: Kellgren-Lawrence grade (KL), Western Ontario and McMaster Universities Arthritis Index (WOMAC), Joint Space Width (JSW), 12-Item Short-Form Health Survey (SF-12), Knee Injury and Osteoarthritis Outcome Score (KOOS), Hip Disability and Osteoarthritis Outcome Score (HOOS)
Other Abbreviations: Symptomatic slow-acting drugs for osteoarthritis (SYSADOA), Cardiovascular diseases (CVD); Coronary heart diseases (CHD); estimated glomerular filtration rate

Non-RCT Experimental

First Author (Year)<br>Study Design | Location<br>N of Patients | Study Length	Glucosamine and/or Chondroitin	Intervention (with Dose) and Comparator	Condition	Measures Used
**Belcaro (2014)**<br>**Non-RCT experimental | Europe**<br>**124 | 4 months**	Glucosamine + Chondroitin	Chondroitin 400 mg + Glucosamine 415 mg 2 capsules/daily<br><br>Comparator: Meriva 500 mg (Curcumin phospholipids complex) + Regenasure (Glucosamine) 500 mg once daily	Osteoarthritis	WOMAC<br>Karnofsky Performance Scale Index<br>Walking distance treadmill test<br>Need for concomitant drugs and medical attention

**Greenlee (2013)**<br>**Non-RCT experimental | United States**<br>**53 | 24 weeks**	Glucosamine + Chondroitin	Glucosamine-sulfate (1,500 mg/day) and chondroitin-sulfate (1,200 mg/day)<br><br>Participants could choose to take either two capsules three times daily or three capsules two times daily<br><br>Comparator: N/A	Joint Pain	OMERACT-OARSI<br>WOMAC<br>M-SACRAH<br>BPI
**Klein (2003)**<br>**Non-RCT experimental | United States**<br>**30 | 20 months**	Glucosamine + Chondroitin	1 to 2 cc injection of solution consisting of 0.5% chondroitin sulfate, 20% glucosamine hydrochloride, 12% DMSO, and 2% Marcaine injected into the intervertebral disc<br><br>Comparator: N/A	Other: Back pain	Roland-Morris scores<br>VAS
**Kubový (2012)**<br>**Non-RCT experimental | Europe**<br>**34 | 6 months**	Glucosamine + Chondroitin	1500 mg of glucosamine sulphate (GS) and 1200 mg of chondroitin sulphate (CHS) daily taken as 3 tablets orally per day (morning, noon, evening)<br><br>Comparator: Placebo	Osteoarthritis	WOMAC<br>Biorheometer evaluation
**Matsuno (2009)**<br>**Non-RCT experimental | Asia**<br>**68 | 3 months**	Glucosamine + Chondroitin	1200 mg glucosamine hydrochloride, 300 mg shark cartilage powder, 111 mg chondroitin, and 45 mg quercetin per day<br><br>Comparator: N/A	Osteoarthritis; Other: rheumatoid Arthritis	VAS<br>JOA<br>ROM<br>Concentration of C4S, C6S, HA, and chondrocalcin in synovial fluid<br>Molecular Weight of HA<br>Viscosity and Stringing of Synovial Fluid<br>Total Amount of Aspirated Synovial Fluid and Protein Concentration
**Muftic (2024)**<br>**Non-RCT experimental | Europe**<br>**60 | 3 months**	Glucosamine + Chondroitin	Cartinorm (1500 mg glucosamine sulfate, 800 mg chondroitin sulfate, 5000 mg forti gel, 250 mg vitamin C) once daily<br><br>Comparator: N/A	Osteoarthritis	VAS<br>Oswestry index
**Persiani (2007)**<br>**Non-RCT experimental | Europe**<br>**12 | 14 days**	Glucosamine only	Glucosamine 1500 mg once daily<br><br>Comparator: N/A	Osteoarthritis	Glucosamine concentrations in plasma and synovial fluid
**Puigdellivol (2019)**<br>**Non-RCT experimental | Europe**<br>**78 | 6 months**	Glucosamine + Chondroitin	Artipotect one tablet three times daily [consisting mainly of hydrolyzed collagen (500 mg/tablet), chondroitin sulfate (180 mg/tablet), glucosamine sulfate (140 mg/tablet), and devil's claw (50 mg/tablet)]<br><br>Comparator: N/A	Osteoarthritis	VAS<br>LFI<br>WOMAC
**Shankland (1998)**<br>**Non-RCT experimental | United**	Glucosamine + Chondroitin	1200 mg of chondroitin sulfate-4 and chondroitin sulfate-6 twice daily, 1600 mg of glucosamine HCI	Temporomandibular Joint Dysfunction	TMJ noises<br>Pain and swelling

**States**<br>**50 | 12 weeks**		twice daily, and 1000 mg of calcium ascorbate taken twice daily<br><br>Comparator: N/A		
**Tokhiriyon (2019)**<br>**Non-RCT experimental | Europe**<br>**27 | 2 months**	Glucosamine + Chondroitin	Biologically active additive (BAA) tablet containing chondroitin sulfate 100 mg and glucosamine sulfate 100 mg (along with other ingredients), taken as 2 tablets in the mornings once a day<br><br>Comparator: No BAA (control)	Osteoarthritis	Pain Index<br>Nail and hair fragility<br>Microcirculation activity test<br>Healing time of postoperative sutures
**Vreju (2019)**<br>**Non-RCT experimental | Europe**<br>**20 | 1 year**	Glucosamine + Chondroitin	500 g glucosamine sulfate, 400 mg chondroitin sulfate, 10 mg collagen type II, and 40 mg Harpagophytum procumbens per day<br><br>Comparator: N/A	Osteoarthritis	Cartilage thickness using musculoskeletal US and MRI
**Weimann (2001)**<br>**Non-RCT experimental | Europe**<br>**9 | N/A**	Glucosamine only	Glucosamine sulfate concentrations at 0.00001 mg/mL, 0.0001 mg/mL, 0.001 mg/mL, 0.01 mg/mL, 0.1 mg/mL and 1 mg/mL with sera samples of patients with HIT and platelets from healthy donors who have not taken any medications<br><br>Comparator: N/A	Other: Heparin-induced thrombocytopenia	Platelet function test<br>PF4/heparin and PF4/glucosamine ELISA

Scales: Outcome Measure in Rheumatology Clinical Trials and Osteoarthritis Research Society International (OMERACT-OARSI) criteria, Western Ontario and McMaster Universities Osteoarthritis Index (WOMAC), Modified Score for the Assessment and Quantification of Chronic Rheumatoid Affections of the Hands (M-SACRAH), Brief Pain Inventory (BPI), Visual Analogue Scale (VAS), Lequesne Functional Index (LFI), Japan Orthopedic Association score (JOA), Range of motion (ROM)
Other Abbreviations: Ultrasonography (US), magnetic resonance imagining (MRI), Chondroitin 4-sulphate (C4S), Chondroitin 6-sulphate (C6S), Hyaluronic Acid (HA), dimethyl sulfoxide (DMSO), Heparin-induced thrombocytopenia (HIT), platelet factor 4 (PF4), temporomandibular joint (TMJ)

Cross-Sectional

First Author (Year)<br>Study Design | Location<br>N of Patients | Study Length	Glucosamine and/or Chondroitin	Intervention (with Dose) and Comparator	Condition	Measures Used
**Ayhan (2024)**<br>**Cross-Sectional | Middle East**<br>**98 | 8 weeks**	Glucosamine + Chondroitin	Combined supplement containing 1250 mg of hydrolyzed type 2 collagen, 750 mg of MSM, 750 mg of glucosamine sulfate, and 400 mg of chondroitin sulfate used once daily<br><br>Comparator: N/A	Osteoarthritis	WOMAC<br>VAS<br>HAQ
**Blakeley (2002)**<br>**Cross-Sectional | Canada**<br>**65 | N/A**	Glucosamine only	Glucosamine (dosing varied)<br><br>Comparator: N/A	Osteoarthritis; Joint Pain; Rheumatoid Arthritis; Fibromyalgia	42 question mail-out questionnaire
**Issa (2021)**<br>**Cross-Sectional | Middle East**	Glucosamine only	TGC-Plus cream (10% glucosamine sulfate and 0.025% capsaicin) 1 gram twice daily	Joint Pain	Numerical pain score tool<br>Joint stiffness

**100 | 12 weeks**		Comparator: N/A		Limitation in joint mobility
**Kantor (2012)**<br>**Cross-Sectional | United States**<br>**9,947 | N/A**	Glucosamine + Chondroitin	Glucosamine, chondroitin, MSM, fish oil, garlic, ginseng, pycnogenol-containing supplements (grape-seed extract, pine bark), ginkgo, and saw palmetto (doses not specified)<br><br>Comparator: N/A	Other: Inflammation	hs-CRP levels
**Kantor (2014)**<br>**Cross-Sectional | United States**<br>**217 | N/A**	Glucosamine + Chondroitin	Glucosamine alone, glucosamine and chondroitin, glucosamine and MSM, or glucosamine and chondroitin and MSM (doses not specified)<br><br>Comparator: N/A	Other: Systemic Inflammation	Biomarkers: hsCRP, Urinary PGE-M, IL-1β, IL-6, IL-8, TNF-α, , soluble TNF receptors I and II
**Lapane (2012)**<br>**Cross-Sectional | United States**<br>**2,679 | N/A**	Glucosamine + Chondroitin	CAM therapies<br><br>Comparator: N/A	Osteoarthritis	CAM prevalence<br>KOOS<br>Joint space narrowing<br>WOMAC<br>SF-12

Scales: Knee Outcomes in Osteoarthritis Survey (KOOS), Western Ontario and McMaster Universities Osteoarthritis Index (WOMAC), Short-Form 12 (SF-12), Health Assessment Questionnaire (HAQ)
Other Abbreviations: Transdermal glucosamine sulfate and capsaicin (TGC), complementary and alternative medicine (CAM), methylsulfonylmethane (MSM), high-sensitivity C-reactive protein (hs-CRP), prostaglandin E2-metabolite (PGE-M), interleukin (IL), , tumor necrosis factor (TNF)

Case-Control

First Author (Year)<br>Study Design | Location<br>N of Patients | Study Length	Glucosamine and/or Chondroitin	Intervention (with Dose) and Comparator	Condition	Measures Used
**Dorais (2018)**<br>**Case-Control | Europe**<br>**758 |**	Glucosamine + Chondroitin	Acetaminophen, NSAIDs, COX-2 inhibitors, narcotics, glucosamine sulfate, and chondroitin sulfate (doses not specified)<br><br>Comparator: N/A	Other: knee replacement	Occurance of Knee Replacement
**Hsu (2019)**<br>**Case-Control | Asia**<br>**864 | 5 years**	Glucosamine only	Glucosamine (dose not specified)<br><br>Comparator: No glucosamine, only NSAIDs	Osteoarthritis	Daily dosage<br>Incidence rate of joint replacement surgery
**Ibáñez-Sanz (2020)**<br>**Case-Control | Europe**<br>**25,811 | 6 years**	Glucosamine + Chondroitin	Chondroitin sulfate, glucosamine, aspirin, indomethacin, diclofenac, aceclofenac, ibuprofen, naproxen, dexketoprofen, and celecoxib (doses not specified)<br><br>Comparator: N/A	Other: colorectal cancer	Incidence of colorectal cancer
**Mazzucchelli (2021)**<br>**Case-Control | Europe**<br>**140,990 | 14 years**	Glucosamine + Chondroitin	Chondroitin sulfate and glucosamine (doses not specified)	Other: AMI	Incidence of AMI<br>Use of chondroitin sulfate and glucosamine

		Comparator: N/A		
**Mazzucchelli (2022)**<br>**Case-Control | Europe**<br>**83,151 | 14 years**	Glucosamine + Chondroitin	Glucosamine and chondroitin sulfate (doses not specified)<br><br>Comparator: N/A	Other: ischemic stroke	Incidence of ischaemic stroke
**Pontes (2018)**<br>**Case-Control | Europe**<br>**22,652 | 5 years**	Glucosamine + Chondroitin	Non-selective NSAIDs, COX-2 selective NSAIDs, glucosamine, chondroitin sulfate, NSAIDs for topical use, opioid analgesics, and other non-opioid analgesics (doses not specified)<br><br>Comparator: N/A	Osteoarthritis; Other: ACE	Incidence of ACE

Other Abbreviations: non-steroidal anti-inflammatory drug (NSAID), cyclooxygenase (COX), acute coronary events (ACE), acute myocardial infarction (AMI)

Case Series/Report

**First Author (Year)**<br>**Study Design | Location**<br>**N of Patients | Study Length**	**Glucosamine and/or Chondroitin**	**Intervention (with Dose) and Comparator**	**Condition/*Condition Caused***	**Measures Used**
**Cerda (2013)**<br>**Case Series | Europe**<br>**151 | 2 months**	Glucosamine + Chondroitin	Glucosamine and/or chondroitin sulfate (dose not specified)	*Liver disease*	Patient questionnaire<br>LFTs
**Chu (2023)**<br>**Case Report| Asia**<br>**1 | 14 days**	Glucosamine + Chondroitin	1500 mg glucosamine sulfate and 1200 mg chondroitin sulfate daily	*Skin rash/allergy*	Physical exam
**Hoban (2020)**<br>**Case Series | Australia**<br>**366 | 11 years**	Glucosamine + Chondroitin	Glucosamine and chondroitin (dose not specified)	*Adverse drug reactions to glucosamine and chondroitin*	Therapeutic Goods Administration Database of Adverse Event Notifications
**Ip (2015)**<br>**Case Report | Canada**<br>**1 | N/A**	Glucosamine + Chondroitin	Glucosamine and chondroitin sulfate 3 tablets per day (dose note specified)	Joint Pain/drug-induced cholestasis	Physical exam<br>Laboratory/imaging data
**Raaijmaakers (2008)**<br>**Case Report | Europe**<br>**2 | N/A**	Glucosamine + Chondroitin	Glucosamine and chondroitin (dose not specified)	Osteoarthritis, Other: Ochronosis	VAS<br>Patient-reported self-assessment
**vonFelden (2013)**<br>**Case Report | Europe**<br>**1 | N/A**	Glucosamine + Chondroitin	Glucosamine and chondroitin sulfate (dose not specified)	*Drug induced acute liver injury*	Physical exam<br>Laboratory/imaging data<br>Immunohistochemistry

Other Abbreviations: Liver function test (LFT)

Other Studies

**First Author (Year)**<br>**Study Design | Location**	**Glucosamine and/or Chondroitin**	**Intervention (with Dose) and Comparator**	**Condition/*Condition Caused***	**Measures Used**

N of Patients | Study Length				
**Arora (2020)**<br>**Randomized, prospective, observational | Asia**<br>**360 | 4 weeks**	Glucosamine only	Celecoxib + Glucosamine sulphate 750 mg twice daily<br><br>Diclofenac sodium + Glucosamine sulphate 750 mg twice daily<br><br>Comparators:<br>Celecoxib only<br><br>Diclofenac sodium only	Osteoarthritis<br>Joint pain	LI<br>VAS
**Conrozier (2019)**<br>**Prospective, Observational | Europe**<br>**2,030 | 6 months**	Glucosamine only	● 2 capsules of GS-Cu once daily for 6 months (each capsule contained 750 mg GS, 1 mg copper sulfate, 50 mg ginger-root extracts, and 12 mg vitamin C)<br>●<br>● Comparator: N/A	Osteoarthritis	Patient self-assessment of treatment observance, reasons for nonadherence, pain scores, treatment efficacy, changes in analgesic intake, and occurrence of adverse events
**Ganti (2018)**<br>**Observational | Asia**<br>**60 | 8 weeks**	Glucosamine + Chondroitin	1.5 g of glucosamine and 1.2 g of chondroitin sulfate daily (group 1)<br><br>Comparators:<br>50 mg tramadol HCL (group 2)<br><br>Sodium hyaluronate 10 mg/mL, 2 mL injection syringe on each joint (group 3)	Temporomandibular joint dysfunction	VAS scale<br>Maximum mouth opening<br>Biomarkers: IL-6, IL-1ß, TNF-α, and PGE2
**Hoffer (2001)**<br>**Pre-clinical | Canada**<br>**28 | 2 days**	Glucosamine only	1 g glucosamine sulfate then (2 days later) 1 g glucosamine sulfate + 1 g acetaminophen<br><br>Comparator: 1 g of anhydrous sodium sulfate	Osteoarthritis	Biomarkers: serum sulfate and synovial sulfate concentration
**Kanzaki (2016)**<br>**Open label, single arm | Asia**<br>**30 | 16 weeks**	Glucosamine + Chondroitin	1,200 mg of glucosamine hydrochloride, 300 mg of shark cartilage extract (60 mg as chondroitin sulfate, 45 mg as type II collagen peptides), 90 mg of quercetin glycosides, 100 mg of fish meat extract (10 mg as imidazole peptides [anserine and carnosine]), 5 mg of salmon nasal cartilage extract (1 mg as proteoglycan), and 5 µg (200 IU) of vitamin D in six tablets taken as six tablets once a day<br><br>Comparator: N/A	Joint pain	JKOM<br>GLFS-5<br>Gait analysis
**Lehrer (2024)**<br>**Retrospective analysis | United States**<br>**N/A | N/A**	Glucosamine only	Glucosamine<br><br>Comparator: budesonide or fluticasone	*Intraocular pressure*<br>*Glaucoma*	Data from UK Biobank, Medwatch, and FinnGen databases

**Martel-Pelletier (2017)**<br>**Post-hoc analysis of an RCT | Canada**<br>**119 | 2 years**	Chondroitin only	Chondroitin sulfate 1200 mg daily<br><br>Comparator: Celecoxib 200 mg daily	Osteoarthritis	Cartilage volume<br>Biomarkers: Hyaluronic acid, CRP, adipsin, leptin, PIIANP, CTX-1, MMP-1, and MMP-3
**Peluso (2016)**<br>**Randomized, controlled, crossover | Europe**<br>**45 | 6 months**	Glucosamine only	Group 1: Oral glucosamine crystalline sulfate (GlcN-S) 1500 mg oral solution for 3 months followed by a combined treatment of both mud-bath therapy and GlcN-S for 3 months<br><br>Comparator: Group 2: Combined treatment of both mud-bath therapy and GlcN-S for 3 months followed by GlcN-S treatment alone for 3 months	Osteoarthritis	Measurements of knee circumferential diameter and ranges of motion<br>WOMAC<br>LFI<br>VAS<br>HAQ<br>GHQ-28
**Persiani (2005)**<br>**Randomized, crossover | Europe**<br>**12 | 22 days**	Glucosamine only	• Crystalline glucosamine sulfate once daily for 3 consecutive days at each of the three selected dose levels (750 mg, 1500 mg, and 3000 mg once daily) in three different study periods separated by a washout period of at least 5 days	None	Plasma glucosamine
**PuigdellívolGrifell (2024)**<br>**Observational | Europe**<br>**186 | 6 months**	Glucosamine + Chondroitin	Artipotect Forte [hydrolyzed collagen (3 g), chondroitin sulfate (0.8 g), glucosamine sulfate (0.7 g), turmeric extract (0.25 g), and devil's claw (0.15 g)] sachet daily in 200 ml of water<br><br>Comparator: N/A	Osteoarthritis	VAS<br>LFI<br>WOMAC

Scales: Visual Analogue Scale (VAS), Lequesne Index (LI), Lequesne Functional Index (LFI), Western Ontario and McMaster Universities Osteoarthritis Index (WOMAC), Japanese Knee Osteoarthritis Measure (JKOM), 5-question Geriatric Locomotive Function Scale (GLFS-5), Health Assessment Questionnaire (HAQ), 28-item General Health Questionnaire (GHQ-28)
Other abbreviations: C-reactive protein (CRP), N-terminal propeptide of collagen IIα (PIIANP), C-terminal crosslinked telopeptide of type I collagen (CTX-1), matrix metalloproteinase (MMP)
