# Supplementary material for: The Safety and Efficacy of Glucosamine and/or Chondroitin in Humans: A Systematic Review
Source: Nutrients. 2025 Jun 24;17(13):2093. doi: 10.3390/nu17132093 (PMC12250884; doi:10.3390/nu17132093)
Supplement: Supplementary file 1 [file nutrients-17-02093-s001.zip › Table S2.pdf]

Table S2. Glucosamine and/or Chondroitin Efficacy Outcomes Across Studies

RCT

Last Name of Author (Year)	Glucosamine and/or Chondroitin Efficacy (with measures of significance, if available)
**Alayat (2017)**	The high intensity laser therapy + glucosamine/chondroitin + exercise group (HILT + GCS + EX) showed significant decrease in VAS as well as in WOMAC subscales (pain, stiffness, and function) after treatment.<br><br>VAS and WOMAC scores were comparable at 6 weeks and 3 months across each group, with HILT + GCS + EX being most effective,<br><br>Pre-VAS score for HILT + GSC + EX group was 7.83 ± 0.72, then 2.04 ± 0.7 after 6 weeks, and 2.73 ± 0.69 after 3 months ($p < 0.0001$)<br><br>Pre-VAS score for GSC + EX was 7.9 ± 0.43, 4.36 ± 0.9 after 6 weeks, and 4.05 ± 0.79 after 3 months ($p < 0.0001$)<br><br>Pre-VAS score for PL + EX was 7.73 ± 0.70, then 3.82 ± 0.73 after 6 weeks, and 4.05 ± 0.79 after 3 months ($p < 0.0001$)<br><br>WOMAC scores were broken into pain, stiffness, and function, with similar patterns between groups as VAS scores.
**Alhayek (2023)**	Significantly decreased after treatment (Jointance gel):<br>● The anterior and posterior glenoid and anterior condylar distances to the pterygoid vertical (PTV) reference plane ($p < 0.001$)<br>● The anterior joint space ($p < 0.001$)<br>Significantly increased after treatment:<br>● The superior distance of the condyle to the Frankfort horizontal reference plane ($p < 0.05$)<br>● The posterior and superior joint spaces ($p < 0.05$)<br>No significant differences between groups:<br>● TMJ linear measurements<br>● Pain and tension levels
**Amalraj (2019)**	Patients treated with both Acujoint and glucosamine/chondroitin had statistically significant improvement in WOMAC score, pain score, functional ability score, visual analog scale (VAS) score and Lequesne functional index values ($p < 0.001$).<br><br>Improvements were significantly better in the Acujoint group compared to the Glucosamine/Chondroitin group in total WOMAC score ($p = 0.006$ and 0.0001), pain score ($p = 0.018$ and 0.0001), functional ability score ($p = 0.016$ and 0.0001), VAS score ($p = 0.009$ and 0.0001) and Lequesne's functional index ($p = 0.031$ and 0.045) at day 60 and day 90, respectively.
**Armagan (2015)**	Post-treatment VAS scores (rest, activation and walking), WOMAC scores (pain, stiffness and function) and 20-m walking times were significantly different from the pre-treatment values ($p < 0.05$) in both the chondroitin sulfate and home exercise groups.<br><br>For MRI findings, there was a significant improvement in only the medial femoral condyle area of the patients in the home exercise group ($p < 0.05$), but no significant differences between the two groups.<br><br>There were no differences between the two groups in any measures.
**Babur (2022)**	There were no significant differences between groups for KOA grade ($p = 1.00$).<br><br>There were no significant differences in the Visual Analogue Scale (VAS), the Knee Injury and Osteoarthritis Outcome Score (KOOS), knee range of motion,

	isometric muscle strength, 5 repetition sit to stand test (5XRSS), and the fall risk score.<br><br>There were no significant differences in body composition parameters other than a greater percentage change in the intervention group in segmental lean mass of the right leg at the 2nd week and of the left leg at the 4th week ($p < 0.05$).
**Basak (2004)**	The Cmax of the powder-filled and timed release formulation (TimeOsamine) was 543.12 ng/ml and 520.98 ng/ml, respectively.<br><br>The tmax of TimeOsamine was delayed by 4.13 h, whereas the powder-filled preparation was 1.00 h.<br><br>The AUC of the 2 doses of TimeOsamine (2 x 500 mg = 1,000 mg) and the 3 doses of the powder-filled formulation (3 x 500 mg = 1,500 mg) was 6,263.32 ng x h/ml and 6,499.55 ng x h/ml, respectively.
**Boeri (2024)**	Follow-up 1:<br>● Sexual symptoms:<br>○ At 3-month follow-up, the total Female Sexual Function Index (FSFI) score significantly improved in both groups (all $p < 0.01$). However, the median FSFI score was higher in the intervention group (capsule with CS and other natural ingredients) than the control group (p < 0 .001). At 3 months, a higher proportion of the intervention group achieved a clinically significant improvement (CSI) in FSFI score (>10 points) as compared with baseline (52% vs 24%, $p = 0.04$. In both groups, all FSFI subdomain scores improved after 3 months of treatment as compared with baseline. FSFI arousal, lubrication, satisfaction, and pain scores at follow-up 1 were higher in the intervention group than the control group (all $p < 0.01$).<br>● Urinary symptoms:<br>○ Median IPSS significantly improved at 3 months vs baseline in the intervention group ($p < 0.01$) and the control group ($p < 0.01$). At follow-up 1, the intervention group showed a lower IPSS as compared with control ($p = 0.03$). A higher proportion of participants in the intervention group vs the control group achieved CMI1 ($p = 0.04$) and CMI2 ($p < 0.01$) in urinary symptoms.<br>Follow-up 2 (after crossover):<br>● Sexual symptoms:<br>○ Median FSFI scores remained stable after crossover in the intervention group, while a significant improvement was reported by participants in the control group vs the 3-month evaluation ($p < 0.01$). After crossover, total FSFI was similar between the intervention and control groups. FSFI arousal, lubrication, satisfaction, and pain scores significantly improved after crossover in the control group when compared with the 3-month evaluation (all $p < 0.01$).<br>● Urinary symptoms:<br>○ Total IPSS significantly improved at 6 months vs follow-up 1 in the control group ($p < 0.01$) but not the intervention group. After crossover, total IPSS was similar between groups. When compared with follow-up 1, storage and voiding symptoms improved after treatment.
**Cahlin (2011)**	There were improvements over time in both groups, and these improvements were statistically significant for all 4 variables in the glucosamine group but only for the VRS in the placebo group. No significant difference was found in any outcome variable between the treatment groups at T1 after 6 weeks of treatment.<br><br>The magnitude of changes from T0 to T1 in all 4 variables did not differ to any statistically significant degree between the glucosamine and placebo arms. The mean difference (and 95% confidence interval [CI]) between the arms in change over time on the visual analog scale (VAS) was 3.7 (95% CI 8.2 to 15.7), on the verbal rating scale (VRS) 0.2 (95% CI 0.5 to 0.9), on opening without pain 1.7 (95% CI 5.1 to 1.6), and on opening with pain 1.2 (95% CI 3.1 to 0.7).<br><br>The improvements in self-rated pain on the VAS from T0 to T1, when split into high or low ratings on the initial VAS, were significantly better in the high-rating subgroups (initial VAS 39 mm) for both treatment arms. The magnitudes of the changes were statistically significantly different from those in the low-rating subgroups (initial VAS 39 and less mm).
**Catanzaro (2013)**	The on-demand use of NSAIDs in both groups was significantly lower as compared to untreated subjects ($p < 0.05$) and similar between the two different treatments.

	Pain assessment:<br>● Starting from the 4-week observation patients treated with LD-1227 showed a rapid and significant decline in VAS score ($p < 0.05$) as compared to the ones supplemented with glucosamine + chondroitin (GC) and to baseline. Several patients reported pain reduction, especially during bed rest at night with the improvement of proper sleep.<br>● As for the assessment of pain during routine physical activity, both treatments yielded a significant and comparable improvement at 4-week observation ($p < 0.05$ vs baseline and vs untreated subjects). However, at 9- and 18-week observation, patients treated with<br>● LD-1227 showed a significantly lower pain intensity as compared to the ones supplemented with GC.<br>● The Lequesne index showed an improvement exclusively following the supplementation of both supplements ($p < 0.01$ vs baseline).<br>● Knee disability and Osteoarthritis Outcome Score (KOOS) scores improvement for function in recreation was statistically significant only in Group A (LD-1227), as early as at week 4 of treatment. At the end of the study the whole parameters of KOOS scores were statistically better in the group treated with LD-1227 as compared to GC.<br>● Pain according to the WOMAC score significantly improved only for Group A, as early as at week 4 ($p < 0.05$ vs GC). Significant improvements in stiffness and difficulty in carrying out normal physical activities were observed by week 4 by both treatments ($p < 0.01$ vs baseline) although LD-1227 achieved significantly better results ($p < 0.05$ vs GC).<br>Biochemistry:<br>● LD-1227 showed a statistically significant reduction of IL-6, hs-CRP and TNF-sR1 already at 4-week observation ($p < 0.05$). The latter two parameters didn't show any significant modification after GC treatment at 4-week observation. At the end of the study period, all tested variables were more significantly reduced by LD-1227 when compared to GC ($p < 0.05$).
**Chopra (2013)**	Significant improvement was seen in each of the intervention groups (active pain, WOMAC pain, WOMAC difficulty, patient global assessment, physician global assessment, and HAQ). The differences between any two intervention groups for the mean change from baseline to completion for primary efficacy measure was within the equivalence range.<br><br>A significant reduction in urinary CTX-II was only observed in the SGCG interventional group (95% CI 1.04, 2.54).
**Clegg (2006)**	The rate of response to glucosamine and chondroitin sulfate, either alone or in combination, was not significantly higher than the rate of response to placebo. However, compared to placebo, the rate of response to chondroitin sulfate was 5.3 percentage points higher ($p = 0.17$), the rate of response to glucosamine was 3.9 percentage points higher ($p = 0.30$), and the rate of response to the combination of glucosamine and chondroitin sulfate was 6.5 percentage points higher ($p = 0.09$).<br><br>The rate of response to the celecoxib control was 10.0 percentage points higher than that for the placebo control ($p = 0.008$).<br><br>The OMERACT–OARSI response rates showed a similar pattern, with no significant differences between the placebo group and the glucosamine, chondroitin sulfate, and combined-treatment groups.<br><br>Analysis of the primary outcome in the subgroup of patients with mild pain showed even smaller treatment effects, with the rate of response ranging from 8.6 percentage points higher in the celecoxib group to 1.9 percentage points higher in the glucosamine group than in the placebo group. None of the differences were significant.<br><br>Treatment effects in the moderate-to-severe pain stratum were more substantial. Results for the primary outcome in this stratum, which included 22 percent of the patients in the trial, indicated that combined treatment was significantly more effective than placebo (24.9 percentage points higher, $p = 0.002$). As compared with placebo, however, celecoxib (difference, 15.1 percentage points; $p = 0.06$), glucosamine (difference, 11.4 percentage points; $p = 0.17$), and chondroitin sulfate (difference, 7.1 percentage points; $p = 0.39$) were not significantly better.<br><br>Similarly, the OMERACT–OARSI response rate ranged from 26.4 percentage points higher with combined treatment ($p = 0.001$) to 10.0 percentage points higher with chondroitin sulfate ($p = 0.24$), as compared with placebo.
**Cohen (2003)**	Between baseline and Weeks 4 and 8, the subjects in the placebo group improved on average in VAS pain, WOMAC, and SF-36 Physical Health. Similar improvements, although of greater magnitude, were seen in the active treatment group (topical glucosamine and chondroitin)..

	VAS scores indicated a greater mean reduction in pain for the glucosamine/chondroitin preparation group compared to the placebo group at both 4 weeks [mean change –2.6 cm vs –1.4 cm] and 8 weeks [mean change –3.4 cm vs –1.6 cm). After 4 weeks the difference between active and placebo groups in their mean reduction from baseline was 1.2 (95% CI 0.1 to 2.4, p = 0.03) and this difference increased to 1.8 after 8 weeks (95% CI for difference between groups, 0.6 to 2.9 cm, p = 0.002).<br><br>Further improvements in VAS pain scores over the 56 days of recording were gradual: at a rate of –0.10 cm/wk (95% CI –0.15 to –0.05 cm) in the placebo group and –0.20 cm/wk (95% CI –0.25 to –0.15 cm) in the active group.
**Cömert Kılıç (2021)**	Pain complaints and joint sounds (p = 0.030 for the control group; p = 0.023 for the study group) showed statistically significant decreases.<br><br>Masticatory efficiency (p < 0.001 for the control group; p = 0.040 for the study group) and lateral mandibular motion (p = 0.040 for the control group; p = 0.004 for study group) showed statistically significant increases in both groups, whereas maximal interincisal opening (MIO) and protrusive mandibular motion showed no significant changes in either group (p > 0.05).<br><br>After estimating the differences between the follow-up and baseline outcomes, the mean changes in the primary outcome variables (VAS scores, MIO, and mandibular motion) showed no statistically significant differences between the two groups (p > 0.05).<br><br>Progressions (reparative remodeling) of hard-tissue TMJ structures were observed on CBCT scans of some participants in both groups.
**Crowley (2009)**	WOMAC score:<br>● The interaction between visit and treatment was significant in UC-II treated group for "pain walking on flat surface" (p = 0.034), "difficulty walking on flat surface" (p = 0.038) and "performing heavy domestic duties" (p = 0.031) as compared to G+C treated group.<br>● UC-II was significantly better than G+C for "ascending stairs at 30 days and 60 days" (p = 0.019 & 0.040 respectively), "at night while in bed" (p = 0.015) at 60 days and difficulty walking on flat surface at 90 days (p = 0.035).<br>● Treatment with UC-II was most effective and reduced the WOMAC scores by 33% compared to 14% in G+C-treated groups after 90 days. Within-group analysis indicated that treatment with UC-II for 90 days significantly (p < 0.05) improved WOMAC scores at all treatment time points measured.<br>Visual analog score (VAS) score:<br>● UC-II was significantly better than G+C for "night pain" (p = 0.040) and "resting pain" (p = 0.020) at 60 days and "pain during climbing up and down stairs" (p = 0.014) and "resting pain" at 90 days (p = 0.034).<br>● Although both the treatments reduced the VAS score, UC-II was found to be more effective with a 40% decrease after 90 days of treatment compared to a 15% decrease in G+C treated groups.<br>● Within-group analysis indicated that subjects on UC-II showed a significant reduction in total VAS scores at Day 60 and Day 90 as compared to baseline. However, subjects on G+C showed a significant reduction in total VAS scores at Day 30 and no significant difference was observed at either Day 60 or Day 90 as compared to baseline.<br>Lequesne score:<br>● UC-II treatment effectively reduced Lequesne's functional index score by 20.1% as compared to 5.9 % by G+C treatment.<br>● Subjects on UC-II demonstrated a significant reduction in total Lequesne's index of severity score from baseline to Day 90, whereas no significant difference from baseline was observed for subjects on G+C at any treatment time points evaluated.
**Czajka (2018)**	Subjects consuming the test product had an overall significant increase in skin elasticity (+40%; p < 0.0001) when compared to placebo.<br><br>Histological analysis of skin biopsies revealed positive changes in the skin architecture, with a reduction in solar elastosis and improvement in collagen fiber organization in the test product group.<br><br>Consumption of the test product reduced joint pain by -43% (p < 0.05) and improved joint mobility (Lysholm score) by +39% (p < 0.05).<br><br>On self-perception questionnaires, 83% of the subjects claimed to have more energy and 70% felt that the supplementation with the test product improved

	their general wellbeing. 78% of subjects in the test product group reported to have less joint discomfort, and more than 60% of the subjects agreed their joint health improved by increasing joint flexibility, mobility, and reducing joint stiffness.<br><br>No significant improvement or change in the responses among subjects consuming placebo was recorded.
**Damlar (2015)**	The reduction in pain levels was significant in both the glucosamine/chondroitin (study) and the tramadol (control) groups. There was no significant difference between two groups in terms of pain reduction.<br><br>The improvement in maximum mouth opening (MMO) was significant with glucosamine/chondroitin ($p = 0.000$) but not with tramadol.<br><br>The MMO improvement was significantly higher in the study group compared to the control group ($p = 0.000$).<br><br>In the study group, a significant decrease was observed in PGE2 level ($p = 0.000$), while the decreases in IL-1β, IL-6 and TNF-α levels were not significant.<br><br>In the control group, no significant decrease was observed in any of the inflammatory cytokines after 8 weeks; moreover IL-1ß and IL-6 levels were increased.<br><br>Alterations of IL-1ß ($p = 0.000$) and IL-6 ($p = 0.010$) levels were significant in the study group while TNF-α and PGE2 levels were not, compared to the control group.
**Das (2000)**	Patients with radiographically mild or moderate OA (n = 72) in the intervention group (glucosamine/chondroitin/manganese) showed significant improvement in the ISK at 4 and 6 months ($p = 0.003$ and $p = 0.04$, respectively).<br><br>The response rate to the medication was 52% vs a 28% response rate to placebo.<br><br>Patients with radiographically severe osteoarthritis (n = 21) did not show significant improvements in the ISK.
**Eraslan (2015)**	Significant improvements were noted in VAS, LYS, and IKDC scores at the end of the study when compared with pre-study values in both glucosamine and placebo groups ($p < 0.001$).<br><br>When the mean changes of scores were compared, no statistical significance was found between the GS and placebo groups ($p > 0.05$).<br><br>The mean percentage deficits of isokinetic values between the operated and healthy knee were compared, and there was no statistically significant difference between the GS and placebo groups ($p > 0.05$).
**Erhan (2012)**	The WOMAC scores for pain, stiffness, and function were significantly improved in both groups (physical therapy plus topical glucosamine-chondroitin sulfate vs. physical therapy plus placebo) at 1 and 4 weeks as compared to the pre-treatment period ($p < 0.05$), however, no difference was determined between pre-treatment and 12 weeks in any of the WOMAC scores. No significant difference was demonstrated between the two groups in WOMAC scores for pain, stiffness, and function at any evaluation ($p > 0.05$).
**Esfandiari (2017)**	N/A
**Filipović (2022)**	One year following the initial assessment, all patients reported pain intensity reduction; however, those in the CGS group experienced significantly lower pain intensity when compared with controls.<br><br>At the first (1-month) check-up, the patients in the NSAID group had lower WOMAC OA index, indicating significant reduction in pain and stiffness, as well as joint function improvement, compared with the CGS group ($p < 0.05$). At the 3-month check-up, lower values of the WOMAC index and similar average reduction in the scores ($p > 0.05$) were registered for both groups. At the 6-month check-up, the CGS group had a significant reduction in the WOMAC pain score ($p < 0.01$), while improvement in stiffness was not statistically significant. Compared with the control group, the CGS group reported a more significant improvement in knee function (based on both WOMAC and Lequesne index). At the final (12-month) check-up, a statistically significant reduction in OA

	intensity ($p < 0.01$) as well as in the Lequesne index ($p < 0.05$) was registered in the CGS group.<br><br>At the end of the study, no reduction in the progression of joint structure damage ($p > 0.05$) was noted in either group.
**Fransen (2015)**	The use of glucosamine-chondroitin resulted in a statistically significant ($p = 0.046$) reduction of 2-year joint space narrowing (JSN) compared to placebo [mean difference 0.10 mm (95% CI 0.002 mm to 0.20 mm)].<br><br>No significant structural effect for the single treatment allocations was detected.<br><br>All four allocation groups demonstrated reduced knee pain over the first year, but no significant between-group differences ($p = 0.93$) were detected.
**Giordano (2009)**	VAS pain scores were significantly lower with GS than placebo during rest at weeks 8, 12, and 16, and during motion at weeks 12 and 16 ($p < 0.05$).<br><br>W-TPS was significantly lower with GS than placebo at weeks 8, 12, and 16 ($p < 0.01$) and at week 20 ($p < 0.05$). W-TSS was significantly lower with GS than placebo at weeks 8, 12, 16, and 20 ($p < 0.05$). W-TPFS was lower with GS than placebo at weeks 8 ($p < 0.05$), 12 ($p < 0.01$), 16 ($p < 0.05$), and 20 ($p < 0.05$).<br><br>Regarding VAS, a statistically significant decrease in pain during rest was observed within the GS group at week 8 ($p < 0.05$), weeks 12 and 16 ($p < 0.001$), and week 20 ($p < 0.05$). Moreover, in the GS group, pain during movement decreased significantly from baseline at weeks 12 and 16 ($p < 0.05$). With GS, W-TPS and W-TPFS were significantly lower than baseline at weeks 8 (both, $P < 0.05$), 12 (both, $p < 0.001$), 16 (W-TPS, $P < 0.001$; W-TPFS, $p < 0.05$), and 20 ($p < 0.05$). W-TSS was significantly lower from baseline at weeks 8, 12, 16, and 20 ($p < 0.05$).<br><br>NSAID and analgesic consumption decreased in the GS group at weeks 4, 8, 12, and 16 ($p < 0.05$).
**Gruenwald (2009)**	There was no statistically significant difference in the number of responders between the two groups for reduction in pain score of ≥ 20% (92.2% group A, 94.3% group B).<br><br>When a higher responder criterion (≥ 80% reduction in the WOMAC pain score) was chosen, the frequency of responders showed a therapeutic and statistical superiority for the combination product of glucosamine sulfate and the omega-3 polyunsaturated fatty acids in patients who complied with the study protocol (group A 44%, group B 32%; $p = 0.044$).<br><br>OA symptoms (morning stiffness, pain in hips and knees) were reduced at the end of the study: by 48.5% to 55.6% in group A and by 41.7% to 55.3% in group B. The reduction was greater in group A than in group B but not significant.
**Herrero-Beaumont (2007)**	Glucosamine sulfate was more effective than placebo in improving the Lequesne score, with a final decrease of 3.1 points, versus 1.9 with placebo [difference between glucosamine sulfate and placebo: -1.2 ($p = 0.032$)].<br><br>The 2.7-point decrease with acetaminophen was not significantly different from that with placebo [difference: 0.8 ($p = 0.18$)].<br><br>There were more responders to glucosamine sulfate (39.6%) and acetaminophen (33.3%) than to placebo (21.2%) ($p = 0.004$ and $p = 0.047$, respectively, versus placebo).
**Hochberg (2008)**	No statistically significant improvement in knee pain compared to placebo was seen among patients randomized to the groups with dietary supplements.<br><br>A subset of patients with moderate-to-severe knee pain at entry who were assigned to the combination of glucosamine and chondroitin sulfate did experience some improvement in knee pain.<br><br>Patients taking chondroitin sulfate were noted to have a statistically significant improvement in knee joint swelling. An exploratory post hoc analysis suggested the effect of chondroitin sulfate on joint swelling occurred more often in patients with milder pain and lower Kellgren-Lawrence Grade at entry.

**Hochberg (2016)**	Clinical outcomes:<br>● The mean change from baseline to 6 months in WOMAC pain score was −185.7 (−200.3 to −171.1) (a decrease of 50.1%) in the chondroitin sulfate plus glucosamine group and −186.8 (−201.7 to −171.9) (a decrease of 50.2%) in the celecoxib group. The corresponding mean difference (95% CI) respected the non-inferiority margin of −40 units: −1.1 (−22.0 to 19.8; $p = 0.92$) in the main analysis.<br>● There were no differences at 6 months between treatment groups in the WOMAC stiffness score, with a decrease of 46.9% in the combination group, compared with a decrease of 49.2% in the celecoxib group ($p = 0.43$); WOMAC function score, with a decrease of 45.5% in the combination group compared with a decrease of 46.4% in the celecoxib group ($p = 0.53$); and visual analogue scale, with a decrease of 48.0% in the combination group versus a decrease of 48.8% in the celecoxib group ($p = 0.92$).<br>● At 6 months, both treatments achieved a 79% response rate ($p = 0.91$). Both groups elicited a reduction from baseline >50% in joint swelling, from 12.5% (33/264) to 5.9% (14/264) for chondroitin sulfate plus glucosamine, and from 14.0% (36/258) to 4.5% (10/258) for celecoxib ($p = 0.54$). A similar reduction was also seen for effusions, from 6.8% (18/264) to 3.0% (7/264) and from 7.8% (20/258) to 4.1% (9/258), respectively ($p = 0.61$).<br>Health-related quality of life:<br>● At 6 months, no differences were apparent between groups in terms of mobility ($p = 0.16$), self-care ($p = 0.94$), usual activities ($p = 0.73$), pain/discomfort ($p = 0.60$), anxiety/depression ($p = 0.21$) or general health status measured by the visual analogue score ($p = 0.54$).
**Kanzaki (2012)**	In GCQ group, all of scores for 'walking', scores for 'stairs – ascending/descending' and the aggregate scores were significantly increased compared to the baseline (indicating clinical improvement) at week 4 or 8 and thereafter ($p < 0.01$ or $p < 0.05$), reaching elevations of 21.5%, 20.4% and 10.4%, respectively, at week 16.<br><br>These scores also improved in placebo at weeks 8 or 12 and thereafter, but the changes were almost one-half smaller than those in the GCQ group at any follow-up time points.<br><br>Individual scores for 'range of motion' and for 'joint swelling' were scarcely changed in both groups throughout the treatment.<br><br>Between-group comparisons in magnitude of changes from baseline revealed that significant differences were seen in scores for 'walking' at week 12 ($p < 0.05$) and week 16 ($p < 0.01$), scores for 'stairs – ascending/descending' at week 16 ($p < 0.05$) and aggregate scores at week 12 ($p < 0.05$) and week 16 ($p < 0.01$).<br><br>Knee joints in both groups had reductions in scores for each of the three pain subscales compared with baseline (indicative of pain relief) with increasing weeks of treatment and reached statistical significance at almost all follow-up time points ranging from week 4 to week 16 ($p < 0.01$ or $p < 0.05$). The extent of reduction in scores for all three pain subscales appeared to be greater in the GCQ group than that in the placebo group.
**Kanzaki (2015)**	In subjects eligible for efficacy assessment, there was no significant group x time interaction. There were improvements in knee-joint functions and locomotor functions in both groups, but there was no significant difference between the groups.<br><br>In subjects with mild-to-severe knee pain at baseline, knee-extensor strength at week 8 104.6 ± 5.0% body weight vs 92.3 ± 5.5% body weight, $p = 0.030$) and the change in normal walking speed at week 16 (0.11 ± 0.03 m/s vs 0.05 ± 0.02 m/s, $p = 0.038$) were significantly greater in the GCQID group than in the placebo group.<br><br>Normal walking speed at week 16 (1.36 ± 0.05 m/s vs 1.21 ± 0.02 m/s, $p < 0.05$) was significantly greater in the GCQID group than in the placebo group in subjects with K-L grade I.
**Kawasaki (2008)**	Comparison performed 18 months later:<br>● Regarding the subcategories, range of motion was improved significantly only in the glucosamine group.<br>● No significant differences were observed between the groups on the scores measured 18 months later ($p = 0.94$). Although a significant difference was observed between the VAS scores at the baseline and those measured 18 months later, no such significant difference was observed between the groups regarding the scores measured 18 months later ($p = 1.95$).<br>● A significant difference was observed regarding the WOMAC score of each group between the scores at the baseline and those measured 18 months later. However, there was no statistically significant difference between the groups regarding the scores measured 18 months later (p =

	1.70).<br>● A significant difference was observed between the scores of stiffness at baseline and those measured 18 months later in the glucosamine and risedronate groups ($p < 0.01$), whereas no significant difference was observed in the control group. However, there was no significant difference between the groups regarding statistical data obtained 18 months later ($p = 1.10$).<br>Factors affecting therapeutic effect:<br>● When comparing the lowest tertile group with the highest tertile, improvement of ΔJapan Orthopaedic Association score (ΔJOA) and ΔWOMAC was observed in the lowest tertile group with a significant difference ($p < 0.05$).
**Khanna (2020)**	Curcumagalactomannosides (CGM)-glucosamine significantly improved the treadmill walking score (baseline 95.36 ± 14.68m, mean difference of 301.35 ± 74.46m; $p < 0.001$).<br><br>Chondroitin-glucosamine had only a minor improvement in the walking score with a mean difference of 89.78 ± 19.25m.<br><br>CGM-glucosamine significantly reduced VAS scores (60.46% improvement) on the 84th day from the baseline ($p < 0.001$). The mean difference was 4.27 ± 0.6.<br><br>The chondroitin-glucosamine group saw a moderate improvement of 21.25% in the VAS score, with an average difference of 1.4 ± 0.4.<br><br>There was a significant improvement in the KPS score of the CGM-glucosamine group ($p < 0.001$) compared to chondroitin-glucosamine.<br><br>A significant variation in the total WOMAC ($p < 0.001$) and pain intensity ($p < 0.001$) scores was observed in the CGM-glucosamine group compared to the chondroitin-glucosamine group.<br><br>CGM-glucosamine was the most effective at easing the difficulty in physical function ($p \leq 0.001$).
**Kongtharvonskul (2016)**	VAS pain score:<br>● At the 24-week mark there was no significant difference between the two groups, with an estimated mean difference of 0.09 (95 % CI -0.75 to 0.94).<br>● The mean VAS scores in the pCGS plus diacerein group at 1, 2, 3, 4, 5, and 6 months were 4.07, 3.58, 3.61, 3.36, 3.32, and 3.15, respectively; the corresponding values in the pCGS and placebo group were 4.72, 4.19, 3.74, 3.47, 3.29, and 2.77. The overall mean VAS scores were 6.72 ± 1.69 and 6.92 ± 1.56 in the combined treatment and monotherapy groups, respectively, but there was no statistically significant difference ($p = 0.607$).<br>WOMAC total score:<br>● At 24 weeks there was no significant difference between the two groups, with an estimated mean difference of -0.1 (95 % CI -14.95 to 14.75).<br>● Mean WOMAC total scores were plotted by treatment and time, which indicated declining WOMAC scores in both treatment groups. The mixed-effects regression model indicated no significant difference between the two groups at each distinct time point.<br>WOMAC pain score:<br>● At 24 weeks there was no significant difference between the two groups, with an estimated mean difference of 0.26 (95 % CI -3.34 to 3.86). Mean WOMAC pain scores were plotted by treatment and time, which indicated declining WOMAC scores in both groups.<br>WOMAC stiffness score:<br>● At 24 weeks there was no significant difference between the two groups, with an estimated mean difference of -0.32 (95 % CI -1.87 to 1.24). Mean WOMAC stiffness scores were plotted by treatment and time, which indicated declining WOMAC scores in both treatment groups.<br>WOMAC function score:<br>● AT 24 weeks there was no significant difference between the two groups, with an estimated mean difference of 0.01 (95 % CI -10.15 to 10.16). Mean WOMAC function scores decreased over time for both treatments. Applying the mixed-effects regression model indicated no significant difference between the two groups at each distinct time point.<br>Minimal joint space width:<br>● At 24 weeks there was no significant difference between the two groups, with an estimated mean difference of 0.04 mm (95 % CI -0.35 to 0.27).

**Kwoh (2014)**	The OR for the likelihood of decreased cartilage damage over 24 weeks in any WORMS-scored subregion of the knee in the glucosamine treatment group compared to the control group was 0.938 (95% confidence interval [95% CI] 0.528, 1.666).<br><br>Compared to subjects treated with glucosamine, control subjects showed more improvement in bone marrow lesion (BML) scores (adjusted OR 0.537, 95% CI 0.291, 0.990) but no difference in worsening BMLs (adjusted OR 0.691, 95% CI 0.410, 1.166) over 24 weeks.<br><br>There was no indication that treatment with glucosamine decreased the excretion of urinary CTX-II (β = -0.10, 95% CI -0.21, 0.002), a molecular marker of cartilage tissue degradation.
**Leffler (1999)**	Statistical significance was seen in the patient assessment of treatment result (p = 0.02) and the visual analog scale for pain at clinic visits (p = 0.02) and in the diary (p = 0.02).<br><br>Results were mainly attributable to improvements in knee symptoms over back symptoms. The overall summary score for the knee data showed a mean change compared with placebo of -16.3% (p = 0.049) and the visual analog scale for pain showed a mean change of -26.6% during the clinic visits (p = 0.048) and -28.6% in the diary data (p = 0.02). Physical exam score showed a mean change of -43.3% (p = 0.01)<br><br>Trends in physical examination scores, acetaminophen use, disability score of Lequesne and Roland, patient assessment of handicap, and physician assessment of severity were not significant. Running times did not change.
**Lomonte (2018)**	The analgesic efficacy evaluated by the investigator in the GS/CS group was 88.9, 95%CI: 75.2, 95.8% and in the Cosamin DS group was 85.4%; 95%CI: 70.1, 93.4%.<br><br>The mean reduction in the pain intensity was significant in both groups (p < 0.001), with no difference between them.<br><br>The primary efficacy analysis demonstrated the non-inferiority of the GS/CS group compared with the Cosamin DS group; the lower limit of the 90% confidence interval (CI) between the two groups (−8.39%) was higher than the established margin of non-inferiority of -10.00%.<br><br>The rate of “no pain” subjects at the end of-treatment visit was 66.7% (95% CI: 50.9 to 79.6%) in the GS/CS group and 52.3% (95% CI: 36.9 to 67.3%) in the Cosamin DS group, and the improvement was observed when compared to V1, statistically significant for both groups (ITT, p < 0.001).<br><br>Mean reduced pain intensity as per VAS in the GS/CS group was 4.27 cm (95% CI: 3.44 to 5.12) and in the Cosamin DS group was 3.58 cm (95% CI: 2.82 to 4.34), in the ITT population. Both decreases were statistically significant (p < 0.001).
**Lomonte (2021)**	Mean reductions of WOMAC pain score were -35.1 mm in the GS/CS group and -36.5 mm in the Condroflex (reference product/RP) group.<br><br>Absolute change in WOMAC total score at 24 weeks: -90 for GS/CS and -93.4 for RP (CI -8.7; 14.4).<br><br>In both PP and ITT population, the upper limit of the confidence interval of the difference between the adjusted mean of both treatments for the pain subscale was lower than the non-inferiority margin of 7 mm established prior to the study, confirming the non-inferiority of the new GS/CS fixed-dose combination versus the RP.
**Lubis (2017)**	At the 12th week, there was a significant difference between the treatment groups on the WOMAC score (p = 0.03) and on the Visual Analog Scale (VAS) score (p = 0.004). When analyzed between weeks, the glucosamine-chondroitin-MSM (GCM) treatment group was found statistically significant on WOMAC score (p = 0.01) and VAS score (p < 0.001). On further subgroup analysis, the glucosamine-chondroitin (GC) treatment group was found statistically significant at the 12th week compared to the placebo group (p = 0.005). While on the 12th week, GCM treatment group was found significant compared to week 0 (p < 0.001).<br><br>At week 12 GCM showed a significant difference in VAS score compared to placebo group (p = 0.001). Within the GCM group itself, there was a significant difference in week 12 compared to week 0 (p < 0.001).

	For score differences, comparison of WOMAC score analysis showed a significant difference between GC, GCM, and placebo in week 4 (p = 0.049) and week 12 (p = 0.01). In addition, VAS scores also showed significant differences between groups in week 8 (p = 0.006) and week 12 (p < 0.001).
**Lugo (2016)**	At day 180, the UC-II group demonstrated a significant reduction in overall WOMAC score compared to placebo (p = 0.002) and GC (p = 0.04).<br><br>Supplementation with UC-II also resulted in significant changes for all three WOMAC subscales: pain (p = 0.0003 vs. placebo; p = 0.016 vs. GC); stiffness (p = 0.004 vs. placebo; p = 0.044 vs. GC); physical function (p = 0.007 vs. placebo).<br><br>No significant changes were observed between the GC and placebo cohorts regardless of the type of analytical model used.<br><br>The UC-II supplemented group had a significant decrease in mean VAS score at day 180 versus both placebo (22.6 vs. 17.0; 95 % CI -9.5 to -1.8; p = 0.002) and GC (22.6 vs. 18.4; 95 % CI -8.0 to -0.4; p = 0.025). In contrast, the GC group was not significant compared to placebo at any time.
**Luo (2022)**	A significant reduction of the total scores from baseline was seen in all treatment groups (p < 0.05) at the end of week 4, 8, and 12.<br><br>Compared with the placebo group, the TII collagen group and G+C group significantly improved the overall joint health measured by the change in WOMAC total score (week 12: TII collagen = -32.47 ± 19.51 and G+C = -33.74 ± 24.64 vs. placebo = -13.84 ± 17.61; p < 0.05) and relieved knee joint pain (week 12: TII collagen = -5.69 ± 3.66 and G+C= -6.03 ± 4.72 vs. placebo = -2.71 ± 3.95; p < 0.05).
**Magrans-Courtney (2011)**	Participants in both treatment and placebo groups experienced significant reductions in body mass (-2.4 ± 3%), fat mass (-6.0 ± 6%), and body fat (-3.5 ± 4%) with no significant changes in fat free mass or resting energy expenditure.<br><br>Perception of knee pain (-49 ± 39%) and knee stiffness (-42 ± 37%) was decreased while maximal strength (12%), muscular endurance (20%), balance indices (7% to 20%), lipid levels (-8% to -12%), homeostasis model assessment for estimating insulin resistance (-17%), leptin (-30%), and measures of physical functioning (59%), vitality (120%), and social function (66%) were improved in both groups with no differences among groups.<br><br>Functional aerobic capacity was increased to a greater degree for those in the supplement group, while there were some trends suggesting that supplementation affected perceptions of knee pain (p < 0.08).
**Mazières (2007)**	At the end of treatment, the decrease in pain was -26.2 (24.9) and -19.9 (23.5) mm and improved function was -2.4 (3.4) (-25%) and -1.7 (3.3) (-17%) in the chondroitin sulphate and placebo groups, respectively (p = 0.029 and 0.109).<br><br>The OMERACT-OARSI responder rate was 68% in the chondroitin sulphate and 56% in the placebo group (p = 0.03).<br><br>The investigator's assessments and short form 12 (SF-12) physical component reported improvement more frequently in the chondroitin sulphate than in the placebo group (p = 0.044 and 0.021, respectively).<br><br>No significant difference was observed between treatment groups for changes in biomarkers over 24 weeks.<br><br>There was a significant difference between non-responders and responders according to the OARSI criteria for 24 week changes of CTX-I (p = 0.018) and CTX-II (p = 0.014).
**Messier (2007)**	WOMAC function and pain did not differ significantly between the groups at 6- or 12-month follow-up.<br><br>There were no significant differences between the groups in 6-min walk or knee strength.<br><br>Balance was better in the placebo group compared to the GH/CS group at 6 months (p = 0.01) and 12 months (p = 0.05).
**Michel (2005)**	Radiographic measures:<br>● Patients who received placebo experienced significant reductions in the mean joint space width (p = 0.001 compared with baseline) and minimum joint space width (p = 0.05 compared with baseline). In contrast, the loss of joint space was null in the CS group. The difference in loss between the

	two groups was significant for the mean joint space width ($p = 0.04$) and for the minimum joint space width ($p = 0.05$).<br>● For 75 patients (36 in the CS group and 39 in the placebo group), the baseline joint space width was 1 mm on the partial flexion view radiographs. The results were similar in the CS group, but the loss was greater in the placebo group; the difference in change between the groups was ($p = 0.006$) and ($p = 0.01$).<br>Symptoms:<br>● Over the 2-year study period, the total WOMAC score did not show a significant improvement.<br>● The intent-to-treat analysis yielded improvement for the CS group on all WOMAC subscales, including pain, stiffness, and function, while the placebo group showed less improvement on the pain and stiffness subscales and some worsening on the function subscale on average.
**Minoretti (2024)**	Both the HA and the Glc + CS groups exhibited significant improvements at the end of the study relative to baseline for observed in pain at rest, pain during movement, range of motion, and the overall WOMAC scores ($p < 0.05$), including its pain, stiffness, and physical function subscales.<br><br>The HA group outperformed the Glc + CS group in the alleviation of pain at rest, pain during movement, and on the WOMAC pain subscale, with all differences being statistically significant ($p < 0.05$).<br><br>Both groups showed a significant elevation in serum adropin levels from baseline ($p < 0.05$), with the HA group experiencing a more substantial increase when compared to the Glc + CS group ($p < 0.05$).
**Monfort (2017)**	fMRI of patella pain showed significantly greater activation reduction under CS compared with placebo in the region of the mesencephalic periaqueductal gray ($p < 0.05$).<br><br>The CS group showed pre/post-treatment activation reduction in the cortical representation of the leg ($p < 0.05$).<br><br>No effects of CS were detected using the interline pressure test.
**Morita (2018)**	Both groups showed a significant improvement in Lequesne's index (LI) and VAS scores at any time compared to baseline ($p < 0.05$). There were no significant differences in LI or VAS scores between the groups at any time point.<br><br>In the subgroup with severe symptoms (Lequesne's index ≥ 8), the chondroitin sulfate dose of 1560 mg/d improved pain faster after 6 and 9 months' therapy.<br><br>No dose-related effects were found on cartilage oligomeric matrix protein or hyaluronic acid levels.
**Nakamura (2007)**	No significant differences were found in swollen joint count and painful joint count between groups.<br><br>Changes of the face scale and VAS for pain showed significant improvement in the glucosamine compared to the placebo group ($p < 0.05$).<br><br>In the glucosamine group, 12.5% of patients exhibited improvement satisfying the ACR20 criteria, whereas in the placebo group, 7.7% of patients exhibited ACR20 improvement.<br><br>ESR and CRP levels did not change, but serum MMP-3 levels significantly decreased in the glucosamine group ($p < 0.05$).<br><br>Results of the patients' self-evaluations and the physicians' global evaluations indicated that the glucosamine treatment produced noticeable improvements in symptoms ($p < 0.05$).
**Nakasone (2011)**	The pain subscale scores decreased time-dependently and reached a significant level at weeks 8, 12 and 16 compared with the baseline ($p < 0.05$) in the test group, whereas no such significant changes were noted at any time points in the placebo group.<br><br>In respect to the JKOM symptom subscales, individual scores for three of the four subscales, namely 'pain/stiffness', 'condition in daily life', and 'general

	activities', as well as scores for the aggregated total symptoms, were all significantly improved at weeks 8, 12 and 16 compared with baseline in the test group ($p < 0.01$).<br><br>Significant decreases in individual scores for the three subscales, 'pain/stiffness', 'condition in daily life', and 'general activities', and scores for the aggregated total symptoms were also noted in the placebo group ($p < 0.05$ each).<br><br>At week 8, significant differences between the test and placebo groups were noted in scores for the 'general activities' (-2.7 ± 2.3 vs. -0.9 ± 1.9; $p < 0.05$) and in scores for the aggregated total symptoms (-13.0 ± 8.9 vs. -5.0 ± 10; $p < 0.05$).<br><br>For VAS pain subscales, the magnitude of score reductions from baseline appeared greater at all of the time points in the test group than in the placebo group; differences in scores for 'pain on walking' between the test and the placebo group at week 8 (-31.6 ± 22.4 vs. -13.4 ± 30.5) and those at week 16 (-41.3 ± 20.5 vs. -21.4 ± 30.5) achieved statistical significance ($p < 0.05$ each).
**Nash (2018)**	In the GC group, the average WOMAC score was reduced by 14.80% on Day 30 and 33.7% on Day 180 compared with the baseline value (Day 0). From baseline, CSE reduced the WOMAC score by 29.80% at Day 30 and 70.3% at Day 180.<br><br>Baseline WOMAC values were not significantly different between the CSE and GC groups. These values, however, were continually reduced throughout the duration of the study in the CSE group, and were significantly ($p < 0.05$) different from the GC group at all the other time points.
**Navarro (2015)**	Statistically significantly lower geometric mean CRP concentrations were observed after the GC intervention compared to placebo (-23%, $p = 0.048$). There were no significant differences in other biomarkers.<br><br>In the proteomics analyses, several pathways were significantly different between the interventions after Bonferroni correction, the most significant being a reduction in the "cytokine activity" pathway (p = 2.6 x 10-16), after glucosamine and chondroitin compared to placebo.
**Navarro (2019)**	Nine genera were significantly different between interventions (FalseDiscovery Rate < 0.05).<br><br>Abundances of four Lachnospiraceae genera, two Prevotellaceae genera, and Desulfovibrio were increased after G&C compared to placebo, while Bifidobacterium and a member of the Christensenellaceae family were decreased.
**Navarro (2020)**	In unadjusted models, mean serum IL-6 decreased (by 24%; $p = 0.01$) from pre- to post-intervention with GH+CS, but was not significant after controlling for multiple testing.<br><br>After adjustment for multiple covariates, IL-6 remained statistically significantly lower after intervention with GH+CS (by 9%; $p = 0.001$), and satisfied the FDR threshold of < 0.05.<br><br>After treatment with celecoxib, there were increases in mean serum CCL20 (by 24%; $p < 0.05$), CSF3 (by 20%; $p = 0.03$), and WNT16 (by 9%; $p = 0.04$), but these biomarkers were no longer statistically significant after controlling for multiple testing.
**Nguyen (2001)**	Effects of administration of active and inactive medications:<br>● CS-GH caused a decrease in the visual analog scale (VAS) from 42 +/- 24 to 36 +/- 28, a decrease of six points, not statistically significant. The mean VAS for the placebo group decreased from 49 +/- 17 to 32 +/- 24, a decrease of 17 points, statistically significant.<br>McGill Pain Questionnaire:<br>● CS-GH for three months did not result in a statistically significant change in the sensory, affective, miscellaneous, total, and number of words Pain Rating Indices (PRI) of the McGill Pain Questionnaire. The evaluative Pain Rating Index decreased from 3.4 to 2.4, which was statistically significant.<br>● The placebo group showed statistically significant decreases in the sensory, evaluative, miscellaneous, and number of words PRI. Affective and total PRI were not statistically significant. The evaluative Pain Rating Index also decreased one point from 3.1 to 2.<br>Mood and Functioning:<br>● The CS-GH group showed a slight decrease in the mood and functioning score that was not statistically significant. The placebo group showed a slight increase that was also not significant.

	TMJ palpation:<br>● The average initial TMJ palpation scores for the inactive and active medication groups were 6.1 and 5.9, respectively. At the end of three months, both groups showed a decrease in the TMJ palpation score, with the CS-GH group being statistically significant ($p = 0.05$), but the placebo group was not ($p = 0.07$).<br>Myofascial pain:<br>● In both CS-GH and placebo groups the myofascial pain score showed improvement but the difference in both groups was not statistically significant.<br>Jaw Range of Motion (ROM):<br>● There was a six millimeter and a three millimeter increase in jaw ROM in the CS-GH group and the placebo group, respectively, but neither was statistically significant
**Nieman (2013)**	● Joint pain severity was significantly reduced in Instaflex (glucosamine) compared to placebo (8-week WOMAC, ↓ 37% versus ↓ 16%, respectively, interaction effect $p = 0.025$).<br>● The pattern of change in the total WOMAC score was not statistically significant between groups 29.4 ± 2.0 to 19.0 ± 1.9, placebo 30.0 ± 2.0 to 24.6 ± 1.0, interaction effect $p = 0.074$).<br>● Interaction effects for the WOMAC joint stiffness (↓ 26% versus ↓ 18%, respectively, interaction effect $p = 0.325$) and joint function index scores (↓ 36% versus ↓ 19%, respectively, interaction effect $p = 0.117$) were not significant.<br>○ Improvements in ability to perform daily activities and stiffness scores in Instaflex compared to placebo were most evident for the 74% of subjects reporting knee pain (8-week WOMAC function score, ↓ 39% versus ↓ 14%, respectively, interaction effect $p = 0.027$; stiffness score, ↓ 30% versus ↓ 12%, respectively, interaction effect $p = 0.081$).<br>● Patterns of change in SF-36, systemic inflammation biomarkers, and the 6-minute walk test did not differ significantly between groups.
**Pavelká (2002)**	Progressive joint space narrowing with placebo use was −0.19 mm (95% confidence interval, −0.29 to −0.09 mm) after 3 years. Conversely, there was no average change with glucosamine sulfate use (0.04 mm; 95% confidence interval, −0.06 to 0.14 mm), with a significant difference between groups ($p = 0.001$).<br><br>Fewer patients treated with glucosamine sulfate experienced predefined severe narrowings (0.5 mm): 5% vs 14% ($p = 0.05$).<br><br>Symptoms improved modestly with placebo use but as much as 20% to 25% with glucosamine sulfate use, with significant final differences on the Lequesne index and the WOMAC total index and pain, function, and stiffness subscales.
**Pelletier (2016)**	Cartilage volume:<br>● In the medial compartment and medial condyle, the cartilage volume loss was significantly less in the CS group at 24 months ($p = 0.018$ and $p = 0.008$, respectively) compared with celecoxib. The results from the generalised linear mixed-model analysis at 24 months showed $p = 0.038$ for the medial compartment and $p = 0.015$ for the medial condyle. However, the results of the adjusted analysis for the ATP population ($n = 120$) showed a significant reduction in cartilage volume loss in CS-treated patients at 12 months for the medial compartment ($p = 0.049$) and at 24 months for the medial compartment and medial condyle ($p = 0.021$ and $p = 0.010$, respectively)<br>The results from the generalized linear mixed-model analysis showed $p = 0.043$ and $p = 0.035$ for the medial compartment at 12 months and 24 months, respectively, and $p = 0.015$ for the medial condyle at 24 months.
**Petersen (2011)**	No differences between groups were observed in gains in muscle cross-sectional area (CSA).<br><br>Training combined with ibuprofen increased maximal isometric strength by an additional 0.22 Nm/kg ($p = 0.04$), maximal eccentric muscle strength by 0.38 Nm/kg ($p = 0.02$), and eccentric muscle work by 0.27 J/kg ($p = 0.04$) in comparison with placebo.

	Training combined with glucosamine increased maximal concentric muscle work by an additional 0.24 J/kg versus placebo (p = 0.01).
**Provenza (2015)**	At 16 weeks, pain reduction, as compared to baseline (GI = -30.9 ± 1.5; GII = -28.7 ± 1.5; GIII = -29.7 ± 1.5 mm) was significant for all groups (p < 0.001).<br><br>Values for the Lequesne's index (LI) were also significantly reduced for all groups (GI = -3.8 ± 0.2; GII = -3.7 ± 0.2; GIII = -3.9 ± 0.2; p < 0.001).
**Puente (2017)**	After 2 weeks of treatment, D-002 and GS/CS reduced significantly (p < 0.00001) the total WOMAC score by 44.6% and 40.2% as compared to baseline. Significant (p < 0.00001) and marked reductions of 72.1% (D-002) and 78.5% (GS/SC) were seen at week 12.<br><br>Both treatments significantly decreased pain (78.6% with D-002, 84.3% with GS/SC), stiffness (82.6% and 89.3%, respectively) and function (67.9% and 74.2%, respectively) WOMAC scores from the second week on therapy.<br><br>At week 2 (first interim check-up), pain score was significantly reduced with D-002 and GS/SC (p < 0.00001 versus baseline for both comparisons).
**Railhac (2012)**	A significant inter-readers correlation was seen for the assessment of cartilage volumes, number of cysts, and osteophytes (correlation coefficients from 0.951 to 0.980 within investigator and from 0.714 to 0.957).<br><br>After 48 weeks, symptoms improved in both groups. The total cartilage volume increased in the Structum group (+180 mm3), whereas there was a loss in total cartilage volume in the placebo (-46 mm3; NS).<br><br>No statistically significant differences between groups were observed for the other MRI parameters.<br><br>No correlations were evidenced between key MRI parameters changes and symptoms.
**Reginster (2001)**	The 106 patients on placebo had progressive joint-space narrowing, with a mean joint-space loss after 3 years of -0.31 mm (95% CI -0.48 to -0.13).<br><br>There was no significant joint-space loss in the 106 patients on glucosamine sulphate: -0.06 mm (-0.22 to -0.09).<br><br>For minimum joint-space narrowing, a significant loss was found with placebo and a non-significant change with glucosamine sulphate (final difference between groups: 0.46 mm; p = 0.033).<br><br>As assessed by WOMAC scores, symptoms worsened slightly in patients on placebo compared with the improvement observed after treatment with glucosamine sulphate. The difference in symptoms between the final group averages was significant (p = 0.016).
**Rindone (2000)**	There was no statistical difference between the two groups in scores on the VAS at 30 days for resting (p = 0.66) or walking (p = 0.69).<br><br>There was also no difference at 60 days for resting (p = 0.81) or walking (p = 0.90).<br><br>There was also no statistical difference between groups in the mean change from baseline in scores on the VAS (p = 0.77).
**Roman-Blas (2017)**	In the modified intent-to-treat population, CS/GS combination therapy was inferior to placebo in the reduction of joint pain (mean ± SD change in VAS global pain score over 6 months 211.8 ± 2.4 mm [19% reduction] in patients receiving CS plus GS versus 220.5 ± 2.4 mm [33% reduction] in patients receiving placebo; peak between-group difference in global pain score at 6 months 8.7 mm [14.2%], p < 0.03)<br><br>There was no difference between groups in the per-protocol completers. The change in VAS global pain scores was similar in both groups (p = 0.072).<br><br>Both placebo treatment and CS/GS combination treatment improved to a similar extent the total WOMAC score as well as the pain and function WOMAC subscale scores, both in the mITT population and in the per-protocol completers.
**Rondanelli (2019)**	The Tegner Lysholm Knee Scoring (TLKS) scale of the CS group showed a statistically significant increase (+10.64 points; p < 0.01), while the WOMAC score decreased (-12.24 points; p < 0.01).

	The results also showed a significant decrease in the CS group compared to placebo in the CRP level (-0.14 mg/dL, CI 95% -0.26; -0.04; $p < 0.01$) and ESR level (-5.01 mm/h, CI 95% -9.18; -0.84, $p < 0.01$).<br><br>The CS group reported an improvement in pain on the VAS for both left and right knees (p = 0.001).
**Rondanelli (2020)**	The between-groups analysis of covariance showed that chondroitin had a significant effect when compared to placebo on the WOMAC scale (p = 0.000) and CRP (p = 0.022).<br><br>For intra-group differences, the result was significant in the CS group for BMI, WOMAC, CRP, total cholesterol and Homeostasis Model Assessment (HOMA) ($p < 0.05$).
**Sawitzke (2008)**	There were no significant treatment differences in JSW loss over two years compared to placebo. The G group had the least average loss (0.013 mm at 2 yrs) [95% CI: −0.379,0.074]; while the G + CS group had the greatest average loss (0.194 mm at 2 yrs) [95% CI: 0.214,0.271].<br><br>JSW loss was greater for K&L Grade 3 than K&L Grade 2 knees and increased with time.<br><br>The unadjusted mean placebo JSW loss was substantially less (0.34 mm) in the placebo group than anticipated by the study design (0.40 mm loss over two years), while it was 0.273 mm for K&L Grade 2 and 0.523 mm for K&L Grade 3 placebo treated knees.
**Scroggie (2003)**	N/A
**Sevimli (2020)**	More than 80% of the patients with OA included in the study relieved pain with the OTC glucosamine-chondroitin supplement.<br><br>There was a significant improvement in functional abilities as assessed by the WOMAC score in patients treated with the OTC supplement.<br><br>There was a statistically significant reduction in the average BMI in the third and sixth months when compared to the baseline values ($p < 0.05$).
**Sterzi (2016)**	VAS at rest was found to be reduced between baseline (T0) and 8 weeks (T1), as well as between T0 and 12 weeks (T2) (F = 13.712; p = 0.0001), with no differences between the experimental and placebo groups (F = 1.724; p = 0.191).<br><br>VAS at motion revealed a significant “group x time-check interaction” (F = 2.491; p = 0.032), with increasing effect of time on VAS reduction (F = 17.748; p = 0.0001). This was most pronounced in the experimental group at 8 weeks (F = 3.437; p = 0.045).<br><br>The Lequesne Index showed reductions at T1 and T2 compared to T0 (F = 9.535; P = 0.0001), along with group effect, since the experimental group presented a lower score at T2 (F = 7.091; p = 0.009).<br><br>No significant changes were found in the knee ROM and inflammation markers.
**Thomas (2021)**	Curcumagalactomannosides (CGM) group individuals showed a tremendous improvement (206%) in the walking performance when compared to the chondroitin sulphate (CHN)–glucosamine (GLN)-treated subjects (85.69%).<br><br>CGM treatment also exhibited a 47.02% reduction in VAS score, whereas there was only 24.67% reduction observed in the group treated with a high dose of CHN–GLN (p = 0.000).<br><br>There was a significant improvement observed in the total (35.01%, p = 0.000), stiffness (32.93%, p = 0.091), and physical function (36.44%, p = 0.000) scores of CGM group when compared to the CHN–GLN group (16.46%, 25.97% and 12.56%, respectively).<br><br>The changes in the WOMAC pain score (31.18%, p = 0.764) of CGM subjects were nonsignificant compared to the double-dose treatment of CHN–GLN

	(26.62%). The CGM group had a 21.42% improvement in the KPS score, which was almost equivalent to the 23.81% improvement expressed by the CHN–GLN group.
**Tío (2017)**	Synovitis presence:<br>● Significant differences were observed in the number of patients with synovitis after 6 months of treatment (16 (80%) acetaminophen (ACT)-treated vs 9 (34.61%) CS-treated patients; p = 0.002), mainly due to a significant reduction in synovitis presence with CS treatment (p = 0.022)<br>● When treatment effect was stratified by initial synovitis condition, a significant decrease was observed in CS-treated patients with initial synovitis (reduction from 18 to 7 patients (61.11%); p = 0.001) and a significant increase among ACT-treated patients without initial synovitis (increased to 6 patients (85.71%) p = 0.031)<br>● Significant differences between CS and ACT treatments were detected regarding the presence of synovitis at the end of the study period: 7 (38.89%) vs 10 (76.92%) in the case of patients with synovitis at baseline (p = 0.036); 2 (25%) vs 6 (85.71%) for patients without synovitis at baseline (p = 0.041), respectively<br>Ultrasound variables:<br>● In the CS-treated group, synovial membrane thickness measured at each time point decreased significantly (p = 0.001), while ACT did not produce any overall change at an ultrasound level.<br>● At the end of the study, synovial membrane thickness was reduced by a mean of 1.5 mm (39.83%, p = 0.003) with CS treatment. CS treatment maintained this significant effect in patients displaying synovitis at baseline (p = 0.001 for global comparison; 1.9 mm (45.42%) synovial thickness reduction after 6 months of treatment, p = 0.004).<br>● The presence of effusion at 6-month follow-up differed by treatment group (9 (45%) in ACT-treated patients vs 4 (15.38%) in CS-treated patients; p = 0.0247)<br>Clinical variables:<br>● Self-assessed pain levels, measured by VAS at all four visits and compared globally, differed significantly only in the patients without synovitis at baseline who were treated with CS (p = 0.028). At 6 months follow-up, a significant reduction was observed not only in this subgroup (19.625 points (34.43%) decrease; p = 0.04) but also in the total group of CS-treated patients (11.42 points (21.87%) decrease; p = 0.0049).<br>● Overall comparison of the Lequesne index data obtained at the four visits showed significant differences only with CS treatment (p < 0.001 for total patients).<br>● At the end of the treatment, both therapies had significantly improved joint function [3.616 points (31.97%) reduction in CS-treated group, p < 0.001; 2.3 points (21.4%) reduction in ACT-treated group, p = 0.016].
**Truong (2019)**	Compared to baseline, there was a statistically significant reduction in the WOMAC score in all three groups. The WOMAC total score was reduced by 13.1, 16.3, and 20.1 points in the control group, Group A, and Group B after 12 weeks of treatment, respectively (p < 0.001). In a paired-wise comparison, there was a greater reduction in the total WOMAC score in Group B compared to the control group, and this difference was statistically significant (p = 0.04)<br><br>WOMAC total score at baseline and after 4 weeks, 8 weeks, and 12 weeks of treatment in the per-protocol population were 43.46, 43.47, and 45.87 in the control group, Group A, and Group B, respectively. After 12 weeks of treatment, these were reduced to 30.67, 27.81, and 25.26 (p < 0.001) in the control group, Group A, and Group B, respectively
**Tsuji (2016)**	A significant group x time interaction was observed on the JKOM score. According to the post hoc test, it significantly decreased (i.e., improved knee function) from the 4- to 12-week follow-up in the Glu/Cho group and the Glu/Cho group score was significantly lower than the control group at the 12-week follow-up.<br><br>There was a significant interaction on household physical activity.<br><br>There was no significant interaction on VAS or physical performance tests.
**Uebelhart (2004)**	Lequesne's algo-functional index (AFI) decreased significantly by 36% in the CS group after 1 year as compared to 23% in the placebo group.<br><br>The intensity of pain decreased respectively by 42% at month 9 and 12 in the CS group vs. 25% in the placebo group. Analysis of variance for multiple comparisons showed a significant difference between both treatment groups at month 9 and 12 (p < 0.05).

	A statistically significant reduction in the mean walking time was observed in the CS group vs. the placebo group as of month 6 onwards ($p < 0.05$) and still improved with time until month 12.<br><br>Radiological progression at month 12 showed significantly decreased joint space width in the placebo group with no change in the CS group.
**Usha (2004)**	Glu, MSM, and their combination significantly improved signs and symptoms of osteoarthritis compared with placebo.<br><br>There was a statistically significant decrease in mean pain index from 1.74 ± 0.47 at baseline to 0.65 ± 0.71 at week 12 with Glu ($p < 0.001$).<br><br>MSM significantly decreased the mean pain index from 1.53 ± 0.51 to 0.74 ± 0.65, and combination treatment resulted in a more significant decrease in the mean pain index (1.7 ± 0.47 to 0.36 ± 0.33; $p < 0.001$).<br><br>After 12 weeks, the mean swelling index significantly decreased with Glu and MSM, while the decrease in swelling index with combination therapy was greater (1.43 ± 0.63 to 0.14 ± 0.35; $p < 0.05$) after 12 weeks.<br><br>The combination produced a statistically significant decrease in the Lequesne index.
**Velickovic (2023)**	A minor increase was observed in the second experimental group (750 mg of GS, 600 mg of naCS, and 200 mg of SAMe) after six months using ultrasonography to evaluate articular cartilage thickness ($p < 0.05$).<br><br>There was no difference in the delta changes between groups for the VAS, TLKS, WOMAC, and SF-36.<br><br>The only serum inflammatory marker outside the reference range was IL-1β, but no significant changes were observed after six months.
**Vicenzino (2019)**	Multiple linear regression analyses indicated that there were no significant differences between the knee guard device (with glucosamine and chondroitin gel) and diclofenac gel for changes in the primary outcomes KOOS-F ($p = 0.58$) and AFS ($p = 0.43$).<br><br>The 95% confidence interval (-2.89 to 5.15) of the estimated treatment difference for KOOS-F between treatments is within the lower (-5.61) and upper (5.61) bounds of the equivalence margin, which suggests that the two treatments are comparable. The 7% equivalence margin for the AFS from the NSAID group was -2.97 to 2.97. The mean difference was 2.13, which is within this equivalence margin. However, the CI for the estimated treatment difference for AFS between treatments was -3.11 to 7.37, which exceeds the equivalence margin, indicating considerable variability in the AFS response.
**Wang (2021)**	At Week 8, significant reductions from baseline in the WOMAC pain (-2.6 ± 1.68, $p < 0.0001$), stiffness (-1.2 ± 1.50, $p = 0.007$), physical function (-5.8 ± 4.39, $p < 0.0001$), and total (-9.4 ± 5.82, $p < 0.0001$) scores were observed in the A+HA group but not in the placebo group.<br><br>Significant differences in the mean change of WOMAC scores from baseline at Week 8 between groups were detected ($p < 0.01$).<br><br>At Week 8, the A+HA group also showed significant improvements in SF-36 physical functioning (2.7 ± 3.10, $p = 0.001$) and bodily pain (0.7 ± 1.50, $p < 0.05$) domains.<br><br>Although the A+HA group had a higher increase in the SF-36 total score than the placebo group, the difference was not statistically significant (2.1 ± 12.75 vs 0.3 ± 19.66, $p = 0.12$).
**Wang (2021)**	After 8 weeks of treatment, the A+HA group failed to demonstrate a significant symptomatic efficacy and quality of life improvement in terms of Knee Injury and Osteoarthritis Outcome Score, WOMAC, SF-36, and Chinese version of Pittsburgh Sleep Quality Index as compared to the placebo group.<br><br>However, the mean changes in most of the SF-36 scale scores were numerically higher in the A+HA group than in the placebo group.
**Wildi (2011)**	Cartilage volume:<br>● CS group compared with those in the placebo group experienced a significant reduction in cartilage volume loss in the global knee at 6 months ($p = 0.030$) that persisted at 12 months ($p = 0.021$). A similar significant reduction was seen at both 6 and 12 months in the lateral compartment ($p =$

	0.015 and p = 0.004, respectively) and the tibial plateaus (p = 0.002 and p = 0.017, respectively).<br>● The reduction in both the lateral and medial tibial plateau was statistically different at 6 months (p = 0.018 and p = 0.016, respectively), with a trend at 12 months (p = 0.068 and p = 0.052, respectively). The lateral condyle also demonstrated a trend towards a reduction at 6 months (p = 0.062) and a significant difference at 12 months (p = 0.006).<br>BML score:<br>● BML data showed no differences between the two groups at 6 months. At 12 months a trend favoring the CS group was found for the global knee (p = 0.062), with a significant difference for the lateral compartment (p = 0.035) and the lateral condyle (p = 0.044).<br>Synovitis:<br>● No difference was found between the two groups during the double-blind phase for the changes in the mean global synovial thickness (both were −0.1 mm) and in the percentage of subjects having joint swelling (CS, 20%; placebo, 23.5%; p = 0.767).<br>● Patients on concomitant CS and NSAID treatment (n = 8) (1.3 ± 0.3 mm) at 6 months demonstrated significantly (p = 0.029) less synovial membrane thickness than the placebo group receiving NSAIDs (n = 10) (1.6 ± 0.3 mm) and a lower incidence of joint swelling (p = 0.092).<br>Symptoms and Function:<br>● No significant difference in disease symptoms assessed by VAS and WOMAX questionnaires (WOMAC total, pain, stiffness and unction) or quality of life (SF-36) at any time point of the study was observed (change in VAS for CS and placebo, respectively, from baseline to 6 months: −14.8 ± 23.7 mm, −20.3 ± 22.1 mm, p = 0.359; from baseline to 12 months:−21.0 ± 27.1 mm, −24.7 ± 25.0 mm, p = 0.607.<br>Change in WOMAC pain subscale:<br>● From baseline to 6 months: −79.7 ± 105.6, −94.4 ± 96.9, p = 0.572<br>● From baseline to 12 months: −99.2 ± 96.7, −124.4 ± 85.3, p = 0.327
**Wilkens (2010)**	At baseline, mean RMDQ scores were 9.2 (95% confidence interval [CI], 8.4-10.0) for glucosamine and 9.7 (95% CI, 8.9-10.5) for the placebo group (p = 0.37).<br><br>At 6 months, the mean RMDQ score was the same for the glucosamine and placebo groups (5.0; 95% CI, 4.2-5.8).<br><br>At 1 year, the mean RMDQ scores were 4.8 (95% CI, 3.9-5.6) for glucosamine and 5.5 (95% CI, 4.7-6.4) for the placebo group.<br><br>No statistically significant difference in change between groups was found when assessed after the 6-month intervention period and at 1 year: RMDQ (p = 0.72), LBP at rest (p = 0.91), LBP during activity (p = 0.97), and quality-of-life EQ-5D (p = 0.20).
**Xia (2016)**	The response rate of a 20% decrease in WOMAC pain in the HA group and GS group was significantly higher than that in the placebo group (differences of 43.5%, p < 0.001 and 25.4%, p = 0.021, respectively), and the response rates in the HA group were significantly greater than those in the GS group (differences of 18.1%, p = 0.037).<br><br>The response rates of 50% decrease in WOMAC pain showed a similar pattern, with significant differences between the placebo group and both the HA group (differences of 43.4%, p < 0.001) and the GS group (differences of 26.9%, p = 0.003). The HA group was not statistically superior to the GS group (differences of 16.5%, p = 0.113).
**Yue (2012)**	The rate of response (20% decrease in WOMAC pain) in the combination group was significantly higher than that in the placebo control (differences of 23.4%, p = 0.006).<br><br>The response rates of 50% decrease in WOMAC pain showed a similar pattern, with insignificant differences between placebo group and chondroitin sulfate group (differences of 12.2%, p = 0.049) as well as the glucosamine group (differences of 11.3%, p = 0.068). The combination group (differences of 15.7%, p = 0.016) was statistically superior to the placebo group.
**Zegels (2013)**	After 3 months of follow-up, no significant difference was demonstrated between the oral daily single dose of CS 1200 formulation and the three daily capsules of CS 400.

	Patients treated with CS 1200 or CS 3*400 were significantly improved compared to placebo after 3 months of follow-up in terms of LI ($p < 0.001$) and VAS ($p < 0.01$).
**Zenk (2002)**	In the glucosamine sulfate-treated group, a significant improvement was found in stiffness and total WOMAC OA Index scores from baseline to week 6 ($p \leq 0.05$ for both) but not in the pain or activities scores.<br><br>There was a significant improvement from baseline to week 6 for the MPC-treated group for all 4 scores ($p \leq 0.005$).<br><br>In the placebo group, no significant changes were found in any of the WOMAC OA Index scores.
**Zhang (2010)**	There was no change in mean joint space in the intervention group between baseline and 8-month follow-up ($p = 0.51$), while in the placebo group, the mean joint space decreased by 0.19 mm over the 8-month treatment ($p < 0.0001$).<br><br>There was no significant difference in mean joint space between two groups after the 8-month follow-up ($p = 0.84$). The results did not change after adjustment for sex and age. There was statistical significance in mean joint-space narrowing in the intervention and placebo group over the 8-month treatment ($p < 0.0001$).<br><br>After 8 months, 24 of 31 patients (77.4%) in the intervention group had a slight mean joint-space narrowing of less than 0.1 mm, compared with seven of 35 patients (20.0%) in the placebo group ($p < 0.0001$).
**Zhang (2021)**	After intervention, the blood routine index IgM rheumatoid factor, albumin/globulin, erythrocyte sedimentation rate and inflammatory factors TNF-α, IL-6, IL-1β, hs-CRP levels in the ComG were evidently better than those in the celecoxib group, while Lequesne score and VAS pain score were lower than those in the celecoxib group ($p < 0.01$).

Cohort

First Author (Year)	Efficacy (with p-values or CIs)
**Bell (2012)**	N/A
**Bhimani (2023)**	N/A
**Cho (2019)**	Treatment patterns:<br>● SYSADOA users used more analgesics (71.9% vs. 58.7%, p=0.08) but less corticosteroids (7.0% vs. 11.6%, p=0.33) compared to patients using NSAIDs without SYSADOA, though the difference was not statistically significant.<br>● The frequency of intra-articular injection was higher in the SYSADOA users than in the non-users (33.3% vs. 9.0%, $p < 0.01$). Intra-articular injection with hyaluronic acid was more frequent in SYSADOA users than in the non-users (26.3% vs. 5.8%, $p < 0.01$). Intra-articular injection with corticosteroids was more frequent in SYSADOA users than in non-users, but the difference was not statistically significant (7.0% vs. 3.2%, $p = 0.25$).<br>Impact of SYSADOA use on the discontinuation of NSAIDs:<br>● 44 patients discontinued NSAIDs during follow-up. Their treatment duration with NSAIDs had a mean 310.0 (± 325.6 days). Of reasons for discontinuation, 25 patients discontinued due to good effects and 4 patients due to ineffectiveness.<br>● The frequency of NSAID discontinuation: during follow-up was similar in the SYSADOA user group (n = 13) and the non-user group (n = 31) at 22.8% vs. 20.0%, respectively ($p = 0.66$). When the reason for NSAID discontinuation was limited to "good effect," the prevalence in each group was not

	different (12.3% in the SYSADOA user group and 11.6% in the SYSADOA non-user group, p = 0.89).<br>● In the Cox proportional hazard model adjusted for age, sex, comorbidities, KL grade in knee X-ray, and total knee replacement (TKR), SYSADOA use was associated with a higher probability of NSAID discontinuation (hazard ratio, HR 2.97, 95% confidence interval, CI 1.42~6.22). Comorbidity with gastrointestinal disease (HR 4.74, 95% CI 1.06~21.27) was also associated with the discontinuation of NSAIDs. In addition, TKR (HR 6.21, 95% CI 2.66~14.49) was associated with the discontinuation of NSAIDs.
**Cho (2023)**	N/A
**Hotaling (2011)**	N/A
**Kantor (2016)**	N/A
**King (2020)**	N/A
**Li (2023)**	N/A
**Lila (2023)**	Changes in the Knee Injury and Osteoarthritis Outcome Score (KOOS) scale:<br>● The mean score increases from baseline to the end of Week 64 were 22.87 (95%CI: 21.56-24.18), 20.78 (95%CI: 19.43-22.13), 16.60 (95% CI: 15.59-17.61), and 24.87 (95% CI: 23.41-26.34) on Pain, Symptoms, Physical Function (KOOS-PS), and Quality of Life subscales, respectively, ($p < 0.001$ for all). For all KOOS subscales, the largest score increases were achieved by Week 16-24 (observation for 4-6 mo), and the achieved effects further remained at the mean values for all subscales with a tendency to increase ($p < 0.001$ for all)<br>Changes in the Hip Disability and Osteoarthritis Outcome Score (HOOS) scale:<br>● The mean increases from baseline to the end of Week 64 were 22.81 (95% CI: 20.47-25.16), 19.93 (95% CI: 17.49-22.36), 18.77 (95% CI: 16.61-20.93), and 22.71 (95% CI: 20.14-25.28) on Pain, Symptoms, Physical Function (HOOS-PS), and Quality of Life subscales, respectively. Similar to changes in the KOOS scale, the highest score increases in the HOOS subscales were achieved by Week 16-24 with a tendency to increase during further follow-up ($p < 0.001$ for all)
**Ma (2019)**	N/A
**Pocobelli (2010)**	N/A
**Raynauld (2016)**	The Jonckheere-Terpstra trend test indicated that treatment with Glu/CS significantly reduced the cartilage volume loss in the global knee, associated with the lateral compartment ($p < 0.05$).<br><br>Multivariate analysis further demonstrated that the extent of the treatment's positive effect was related to exposure time to treatment, the protective effect at 6 years being significant in participants exposed to 2 or more years of treatment ($p < 0.05$).
**Roubille (2015)**	In the (-)analgesics/NSAIDs group, Ext+ participants taking Glu/CS had significantly less cartilage volume loss in the medial plateau at 24 months ($p \leq 0.010$, univariate and multivariate analyses).<br><br>In the (+)analgesics/NSAIDs group at 24 months, Ext- participants taking Glu/CS had less cartilage volume loss in the global ($p \leq 0.002$, univariate and multivariate analyses) and medial and lateral plateaus ($p = 0.034$ and $p = 0.013$, respectively, multivariate analysis).<br><br>No significant difference in JSW loss was found between the groups.

Rovati (2016)	Of 6451 patients in the PEGASus cohort, 315 patients received crystalline glucosamine sulfate, they were exposed for 481 2-month time units and had an incident use of NSAIDs of 18.7%.<br><br>In the control cohort, NSAID incident use was 23.8%.<br><br>Crystalline glucosamine sulfate significantly decreased the risk of NSAID consumption by up to 36% (OR = 0.64; 95% CI: 0.45-0.92) in the primary analysis foreseen by the protocol. The OR for crystalline glucosamine sulfate was 0.74 (95% CI: 0.54-1.01) in a sensitivity analysis accounting for an extension of the study and of the control cohort.<br><br>None of the other SYSADOAs showed any decrease in the use of NSAIDs.
Yang (2015)	During the study period, 18% of the participants initiated treatment with glucosamine/chondroitin.<br><br>After adjustment for potential confounders with marginal structural models, we found no clinically significant differences between users at all assessments and never-users of glucosamine/chondroitin in WOMAC Pain: 0.68 (95% CI: -0.16 to 1.53); WOMAC Stiffness: 0.41 (95% CI: 0 to 0.82); WOMAC Function: 1.28 (95% CI: -1.23 to 3.79); or JSW: 0.11 (95% CI: -0.21 to 0.44).
Yu (2022)	N/A
Zheng (2023)	N/A
Zheng (2023)	N/A
Zhou (2023)	N/A

Non-RCT Experimental

First Author (Year)	Efficacy (with p-values or CIs)
Belcaro (2014)	Karnofsky Index was significantly higher in the Meriva + glucosamine than in the chondroitin + glucosamine group:<br>● Meriva + glucosamine:<br>○ Baseline: 71.2 ± 5.4<br>○ 4 months: 93.4 ± 6.4<br>○ p < 0.05 vs baseline<br>● Chondroitin + glucosamine:<br>○ Baseline: 79.6 ± 6.6<br>○ 4 months: 71.6 ± 6.2<br>○ P < 0.05 vs chondroitin+glucosamine<br>A similar finding was reported for the WOMAC scores, both in the physical and emotional domains.<br>The walking distance at the treadmill test was significantly higher in the Meriva + glucosamine group than in the control group already at 1 month:<br>● Meriva + glucosamine:<br>○ Baseline: 85.6 ± 12.0<br>○ 1 month: 213 ± 15.*+<br>○ 2 months: 278 ± 21.1*+<br>○ 4 months: 374 ± 31.4*+<br>○ *p < 0.05 vs chondroitin+glucosamine; + p < 0.05 vs baseline

	• Chondroitin + glucosamine:<br>• Baseline: 88.3 ± 18.4<br>○ 1 month: 102 ± 19.6<br>○ 2 months: 167.3 ± 16+<br>○ 4 months: 224.5 ± 32.6+<br>○ +p < 0.05 vs baseline<br>The need for concomitant drugs and medical attention decreased in both groups: however, the use of Meriva + glucosamine was associated with a reduced need for concomitant drugs and medical attention than the association of chondroitin+glucosamine.
**Greenlee (2013)**	From baseline to week 24, 46% of patients had improved pain/stiffness according to OMERACT-OARSI criteria.<br><br>At week 24, there were improvements in pain and function as assessed by WOMAC and M-SACRAH, and in pain interference, severity, and worst pain as assessed by BPI (all $p < 0.05$).<br><br>Estradiol levels did not change from baseline.<br><br>At week 12, approximately one third of women demonstrated ≥20 % improvement in pinch, tripod, and power grip strength (via Martin dynamometer), which was maintained at week 24. Participants also demonstrated improvements in mean scores in pinch (right hand, $p = 0.07$), tripod (right hand, $p = 0.02$), and power (right hand, $p = 0.01$) grip strength.<br><br>There were no changes in physical well-being or the endocrine symptom sub-scale from baseline to 12 or week 24.<br><br>There were no reported differences in use of analgesics from baseline to week 12 or 24 ($p > 0.05$).
**Klein (2003)**	Nonresponders:<br>• There were a total of 13 patients who responded minimally with average improvements in visual analogue scores of 14% and disability scores of 8% ($p < 0.001$). All four patients who were disabled for more than 1 year failed to respond.<br>Responders:<br>• Seventeen patients were judged to have a good or excellent response by virtue of an improvement in disability and/or visual analogue pain scores of at least 50%. There was an average reduction in the disability score of 72% and an average reduction of the visual analogue pain score of 76% in these 17 patients. The response to treatment was gradual in all patients, but all responders had significant improvements after the first treatment, whereas nonresponders failed to show significant improvements with the first or subsequent treatments.<br>• Three of the seven patients with prior IDET procedures were in the excellent response group, and four were in the nonresponder group.
**Kubový (2012)**	Slight deterioration in all of the WOMAC parameters (knee pain, knee stiffness, daily activities) was observed in the untreated group but only achieved a significant level for dissipated energy ($p < 0.05$).<br><br>The treated group (glucosamine and chondroitin) improved in all the parameters. Statistically significant difference was found for energy dissipation in the joint during movement ($p < 0.05$).<br><br>Rheological properties of at least one knee improved in 21% of patients in the treatment group. This was not observed in the control group.<br><br>The positive effects of the SYSADOA persisted for 3 months after the end of treatment.
**Matsuno (2009)**	OA patients showed a significant improvement in pain symptoms, daily activities (walking and climbing up and down stairs), and visual analogue scale ($p < 0.05$). OA patients also had significant changes in the synovial fluid properties with respect to the protein concentration, molecular weight of hyaluronic acid, and chondroitin 6-sulphate concentration ($p < 0.05$).

	These effects were not observed in the RA patients.
**Muftic (2024)**	Assessment of pain through the VAS pain scale on the first day and at the end of the 3-month study showed a statistically significant reduction in pain ($p < 0.05$).<br><br>Analysis of the quality of life at the beginning of the study showed that 22 subjects performed activities with many difficulties, and at the end of the study only 5 subjects performed activities with many difficulties.
**Persiani (2007)**	There was a good correlation between the endogenous glucosamine concentrations in plasma and synovial fluid (Spearman's $r = 0.78$, $p < 0.01$). On the other hand, endogenous synovial fluid concentrations were lower ($p = 0.001$) than those in plasma (median value 45.5%)<br><br>The relative increases from baseline were similar in the two compartments, with only a marginally higher median increase in the synovial fluid compared with plasma: 21.5 folds vs. 20.5 folds ($p = 0.11$), suggesting that the drug has a similar distribution between the two compartments<br><br>Similarly to endogenous levels at baseline, absolute post-treatment glucosamine concentrations were higher in plasma than in the synovial fluid ($p = 0.001$), with a median synovial/plasma concentration ratio of 76.5% (i.e., the median concentration in synovial fluid was only 23.5% lower than that in plasma). There was a very high degree of correlation between post-treatment concentrations in the two compartments (Pearson's $r = 0.96$, $p < 0.0001$)
**Puigdellivol (2019)**	Patients showed a reduction of pain of 3.77 ± 1.77 points after 6 months ($p < 0.0001$) in the VAS.<br><br>The total reduction in the Lequesne Functional Index was 6.30 ± 4.08 points after 6 months ($p < 0.0001$), with significant reductions in all subindexes of the scale.<br><br>A similar pattern was found for the WOMAC index, with an overall reduction of 22.49 ± 14.03 points after 6 months ($p < 0.0001$) and significant reductions in all subindexes.
**Shankland (1998)**	80% of those in the study reported a decrease in TMJ noises with an associated decrease in joint pain and swelling.<br><br>Of the remaining 20%, 2% reported worse symptoms than before entering the study, 10% did not comply with the study, and 8% reported no noticeable change in symptoms.
**Tokhiriyon (2019)**	In 10 patients, a positive effect was noted on the 14th day of treatment. The subjects noted a decrease in joint soreness, which was confirmed by a reduction in the pain index. Patients who took the product also noted the improved appearance and strengthening of hair and nails.<br><br>The observations of the doctor-cosmetologist, conducted before and after two-month intake of BAA, revealed a reduction in dryness of the skin of the face and body in 57% of the examined cases. In 64% of the clients, the complex helped to reduce swelling, and in 71% it improved the contour of the face.<br><br>A test for the microcirculation activity (nail bed reaction) showed that the subjective sensation of improvement in complexion and external attractiveness was accompanied by the activation of the microcirculatory bed.<br><br>Where at the beginning of BAA intake, the same reaction of the nail bed to depression was observed in both groups, by the end of the treatment period, the speed of the microcirculatory bed increased significantly.<br><br>When assessing the state of postoperative suture dynamics, it was shown that the inclusion of the specialized product in the rehabilitation process improved healing. The scar in patients taking BAA was characterized by greater tenderness, less hyperemia, and infiltration.
**Vreju (2019)**	The US examination permitted the detailed evaluation of the femoral hyaline cartilage thickness, with statistically significant differences before and after treatment at the level of the medial compartment, both in the dominant (1.59 ± 0.49 vs. 1.68 ± 0.49, $p = 0.0013$) and non-dominant knee (1.73 ± 0.53 vs. 1.79 ± 0.52, $p = 0.0106$).

	The US and the MRI correlated well (r = 0.63) and showed no radiographic progression in knee osteoarthritis after one year of treatment with specific SYSADOA. The US also showed improvement in the cartilage thickness of the medial compartment.
**Weimann (2001)**	N/A

Cross-Sectional

First Author (Year)	Efficacy (with p-values or CIs)
**Ayhan (2024)**	The median VAS-pain score decreased from 6 at Visit 1 to 3 at Visit 3.<br><br>From Visit 1 to Visit 3, the median total WOMAC score decreased from 26.04 to 9.38, WOMAC-pain subscale score from WOMAC-stiffness score from 25 to 12.50, and WOMAC-physical function score from 26.47 to 10.29. Regarding the quality of life, HAQ scores also decreased from 0.40 to 0.15 from Visit 1 to Visit 3.<br><br>For all scores, the differences between the three visits were statistically significant ($p < 0.001$ for all). Moreover, the differences between Visit 1 and Visit 2, Visit 1 and Visit 3, and Visit 2 and Visit 3 were also significant ($p < 0.001$ for all).<br><br>For Visit 2 and Visit 3, the patients reported that the preparation of the supplement was easy and that the daily dose, taste, odor, appearance, and density of the supplement were suitable. The patient compliance with the supplement was a median of 96.77% both for Visit 2 and Visit 3.
**Blakeley (2002)**	The factors that had the largest influence over the decision to take GLS were advice from friends (40%; n = 26), magazine articles, newspapers and books (29%; n = 19), family members (26%; n = 17), family practitioners (20%; n = 13), and health-store personnel (15%; n = 10). Information and advertisements provided by television (15%; n = 10) or found in the Internet (11%; n = 7) also impacted on the decision making.<br><br>Fifty-seven percent (n = 37) indicated they always used the same brand. A smaller number stated they had no brand of choice. Approximately one-third identified cost as the reason for choosing a particular brand. Other reasons included quality issues, availability, satisfaction with results, and the desired combination of ingredients and/or dosages.<br><br>The vast majority (86%; n = 56) reported that they were managing and monitoring their own GLS treatment. The remaining 8% (n = 5) stated their doctor was responsible for this management. All participants had been using GLS for less than 5 years. Forty-three percent (n = 28) had been taking it for a year or less; 31% (n = 20) from 1 to 3 years; and 23% (n = 15) from 3 to 5 years. Eighty-two percent (n = 53) stated they had taken the product regularly but only one-third (n = 21) took the average recommended dose of 1500 mg per day. Over one-third took 1000 mg or less (n = 24) or 2000 mg (n = 3).<br><br>Some only took 500 mg daily while others took the same amount twice or three times per day. Still others used higher doses of 1000 mg once or twice daily. Many (37%; n = 24) determined the dose themselves after reading instructions on the bottle. Others relied on information found in articles, books or on television and the Internet and/or the advice of friends or family members, pharmacists, and health-food store personnel.<br><br>The few (18%; n = 12) who stopped taking GLS did so because of cost, ineffectiveness, inability to obtain the product, and/or preference for other treatments such as anti-inflammatory drugs. The vast majority of the subjects perceived GLS to be at least somewhat beneficial in the treatment of arthritis symptoms. Forty-five percent (n = 29) found it very helpful, 22% (n = 14) moderately so, and 11% (n = 7) perceived that it helped a little. Only five individuals (8%) found the product to be not at all helpful.
**Issa (2021**	There was a significant reduction of numerical pain score (7 ± 1.40 vs. 3.53 ± 2.13, $p < 0.05$), with significant reduction in the limitation of joint movement (6.18 ± 2.14 vs. 3.47 ± 2.23, $p < 0.05$) after 12 weeks.<br><br>The need for analgesics and the number of doctor's visits were significantly reduced (1.99 ± 2.77 vs. 0.71 ± 1.90, $p < 0.05$), (1.11 ± 1.28 vs. 0.06 ± 0.293, $p < 0.05$) respectively.

Kantor (2012)	In the fully adjusted model, regular use of glucosamine was associated with a statistically significant 17% reduction in hs-CRP levels (as compared with nonuse) (ratio = 0.83, 95% confidence interval (CI): 0.74, 0.93) and chondroitin was associated with a 22% reduction in hs-CRP (ratio = 0.78, 95% CI: 0.67, 0.92). Regular use of fish oil was also associated with a significant 16% reduction in hs-CRP levels (ratio = 0.84, 95% CI: 0.71, 0.997).<br><br>The study observed significant interactions by gender for the associations of glucosamine use with hs-CRP (P-interaction = 0.05) and chondroitin use with hs-CRP (P-interaction = 0.03). Among women, regular glucosamine use was associated with a 27% reduction in hs-CRP (ratio = 0.73, 95% CI: 0.61, 0.88), and regular chondroitin use was associated with a 33% reduction in hs-CRP (ratio = 0.67, 95% CI: 0.53, 0.84), while the associations among men were small and nonsignificant. Lastly, the study observed a significant interaction between ginseng use and gender (P interaction = 0.03), with the association being evident in men (ratio = 0.84; 95% CI: 0.72, 0.98) but not in women.
Kantor (2014)	In multivariate models, high users (> 14 pills/week) of glucosamine had 28% lower hsCRP than nonusers (ratio: 0.72; 95% CI, 0.47-1.08; p for trend = 0.09) and high users of chondroitin had 36% lower hsCRP than nonusers (ratio, 0.64; 95% CI, 0.39-1.04; p for trend = 0.03).<br><br>Compared with nonusers, high users of glucosamine had 24% lower PGE-M (ratio, 0.76; 95% CI, 0.59-0.97; p for trend = 0.10), while high users of chondroitin had 27% lower PGE-M (ratio, 0.73; 95% CI, 0.55-0.98; p for trend = 0.07). It appeared that low use (< 14 pills/week) of glucosamine and chondroitin led to a partial benefit in terms of CRP reduction, but reduction in PGE-M was limited to those who took at least 2 pills per day.
Lapane (2012)	CAM use was prevalent (47%), with 24% reporting use of both CAM and conventional medication approaches. In addition, 54% used chondroitin and 59% used glucosamine.<br><br>Multi-joint OA was correlated with all treatments [adjusted odds ratios (aOR) conventional medications only: 1.62; CAM only: 1.37 and both: 2.16].<br><br>X-ray evidence of severe narrowing (OARSI grade 3) was associated with use of glucosamine/chondroitin (aOR: 2.20) and use of both (aOR: 1.98).<br><br>The Western Ontario and McMaster Universities (WOMAC)-Pain Score was correlated with conventional medication use, either alone (aOR: 1.28) or in combination with CAM (aOR: 1.41 per one standard deviation change).<br><br>Knee Outcomes in Osteoarthritis Survey (KOOS)-Quality of Life (QOL) and Short Form (SF)-12 Physical Scale scores were inversely related to all treatments.

Case-Control

First Author (Year)	Efficacy (with p-values or CIs)
Dorais (2018)	N/A
Hsu (2019)	The mean treatment duration of the glucosamine-treated group (40.38 ± 7.89 months) was less than the NSAID-treated group (45.82 ± 3.89 months).<br><br>The most common medication-taking habit was 250 mg 3 times a day for 3 months and discontinued for 3 months.<br><br>Only 0.7% of patients used the recommended daily dosage of 1500 mg.<br><br>Patients using GS had a higher incidence rate of joint replacement surgery than those who did not use GS (p = 0.001).
Ibáñez-Sanz (2020)	N/A
Mazzucchelli (2021)	N/A

**Mazzucchelli (2022)**	N/A
**Pontes (2018)**	N/A

Case Series/Report

First Author (Year)	Efficacy (with p-values or CIs)
**Cerda (2013)**	N/A
**Chu (2023)**	N/A
**Hoban (2020)**	N/A
**Ip (2015)**	N/A
**Raaijmaakers (2008)**	Using 1500 mg of glucosamine and 400 mg of chondroitin sulfate:<br>● The first patient with (diagnosed with ochronosis) reported a VAS of 8/10 before treatment and a VAS of 0/10 after four months of initiating the treatment.<br>● The second patient (diagnosed with pain in knee and lower back stiffness) reported a VAS of 10/10 before treatment and a VAS of 5.5/10 after four months of initiating the treatment.<br>● Both continued the treatment on their own initiative, reported a positive effect on daily activities and started again to do some walking, swimming and cycling. They also reported improvement in their mental status.
**vonFelden (2013)**	N/A

Other Studies

First Author (Year)	Efficacy (with p-values or CIs)
**Arora (2020)**	Lequesne Index Score:<br>● Highest (12.68 ± 2.23) was found in the second group (glucosamine sulphate with celecoxib) and the lowest (9.13 ± 2.47) was with the diclofenac-treated group as compared within all groups.<br>● Data was calculated at different time intervals and showed the highest percentage at week 4 with the combination of glucosamine sulphate and diclofenac sodium (47.70%) as compared with other treated groups respectively (18.60%; 14.27 % and 31.65%) and similar data was also found to be at week 2.<br>● The diclofenac-treated group showed the highest percentage (31.65%), when compared with the celecoxib-treated group and in the combination of glucosamine sulphate with celecoxib-treated group.<br>● The p-value for Lequesne Index data was $p < 0.0001$.<br>Visual Analogues Score (VAS):<br>● Value revealed 5.22 ± 1.44 with the treated group (combination of glucosamine with diclofenac sodium) than the other combination (glucosamine sulphate with celecoxib treated group), the value was 7.22 ± 1.99.<br>● The VAS score was in order of glucosamine sulphate with diclofenac sodium > diclofenac sodium > celecoxib > glucosamine with celecoxib (37.16% > 31.05% > 24.87% > 18.14%).<br>● The p-value for VAS data was $p < 0.0001$.

**Conrozier (2019)**	Medication compliance was self-reported as good by 80% of patients.<br><br>At the end of the study, pain scores were significantly lower than at baseline (1.8 ± 2.0 and 3.5 ± 3.2 at rest and during exercise, respectively; $p < 0.001$).<br><br>83% reported improvement in pain.<br><br>13% rated the treatment as highly effective, 50% effective, 25% moderately effective, and 12% ineffective.<br><br>Observance was weaker in active compared to retired patients ($p = 0.005$), in patients not taking concomitant treatment ($p = 0.008$), or who had never been treated for OA ($p = 0.001$). Observance was correlated with pain decrease ($p < 0.0001$) and with lack of adverse effects ($p < 0.001$).
**Ganti (2018)**	Pain VAS score and maximum mouth opening improved in all three groups ($p < 0.05$).<br><br>In group 1 (glucosamine and chondroitin), pain VAS score was 7.12 ± 2.8 before and 3.20 ± 1.5 after treatment ($p = 0.01$). In group 2 (tramadol), pain VAS score was 7.28 ± 2.5 before and 3.25 ± 2.2 after treatment ($p = 0.02$). In group 3 (sodium hyaluronate), pain VAS score was 7.55 ± 3.4 before 3.38 ± 2.7 after treatment ($p = 0.01$).<br><br>Maximum mouth opening (MMO) improved in all three groups ($p < 0.05$).<br><br>IL-1 ß and IL-6 significantly decreased after treatment in group 1 ($p < 0.05$).<br><br>IL-1 ß significantly increased in group 2 ($p < 0.05$).<br><br>There were no significant differences in inflammatory mediators in group 3.
**Hoffer (2001)**	The serum sulfate concentration of 7 normal subjects was 331 mol/L before ingestion of 1.0 g glucosamine sulfate and 375 mol/L 3 hours after ($p < 0.05$). Serum sulfate concentrations decreased from 325 to 290 mol/L when the same dose of glucosamine sulfate was ingested with 1 g of acetaminophen ($p < 0.05$).<br><br>Oral sodium sulfate did not significantly increase the serum sulfate concentration ($p = 0.15$).<br><br>Synovial fluid and serum sulfate concentrations were closely similar when measured in 15 patients undergoing diagnostic needle aspiration of a knee effusion ($r = 0.99$, slope = 0.97, $p < 0.0001$).
**Kanzaki (2016)**	The VAS score for JKOM knee pain, JKOM total score, GLFS-5 score, and all VAS scores for knee pain in various daily situations significantly decreased at week 4 and thereafter ($p < 0.05$ or $p < 0.01$) compared with the baseline.<br><br>The questionnaire scores reflecting frequency of tripping while walking or ascending/descending stairs significantly decreased at week 8 and thereafter ($p < 0.05$ or $p < 0.01$) compared with the baseline.<br><br>At 16 weeks, normal walking speed ($p < 0.01$), stride length ($p < 0.01$), and angle of soles at the end of the stance phase were all significantly increased ($p < 0.01$), but cadence did not change significantly during the intervention period.<br><br>There were significant intercorrelations of changes in normal walking speed, stride length, angle of soles at the end of the stance phase, between changes in stride length, and total JKOM score.
**Lehrer (2024)**	N/A
**Martel-Pelletier (2017)**	In patients with levels of biomarkers of inflammation (HA, leptin and adipsin) lower than the median, those treated with chondroitin sulfate

	demonstrated less cartilage volume loss in the medial compartment, condyle, and plateau ($p \leq 0.047$).<br><br>Patients treated with chondroitin sulfate with higher levels of MMP-1 and MMP-3 (biomarkers of cartilage catabolism) had less cartilage volume loss in the medial compartment, condyle, and plateau ($p \leq 0.050$).<br><br>Patients with higher levels of PIIANP and CTX-1 (biomarkers related to collagen anabolism and bone catabolism, respectively) had reduced cartilage volume loss in the medial condyle ($p \leq 0.026$) in the chondroitin sulfate group.
**Peluso (2016)**	In group 1 (GlcN-S for 3 months followed by combined treatment with mud-bath), no significant difference was shown in the comparison from month 0 to month 3. Significant improvements were seen from month 3 to month 6 on Flexion ROM (p = 0,041), Extension ROM (p = 0.0006), Lequesne algo-functional index (p = 0.002), and HAQ (p = 0.005).<br><br>In group 2 (combined treatment with GlcN-S and mud-bath for 3 months followed by GlcN-S alone), the comparison between month 0 and month 3 showed a significant improvements in Flexion ROM (p = 0.036), Extension ROM (p = 0.011), WOMAC index (p = 0.001), Lequesne algo-functional index (p = 0.0001), VAS (p = 0.001), HAQ (p = 0.007) and GHQ-28 (p = 0.007). When comparing month 3 and month 6, despite an improvement of all the variables, no significant difference was shown.
**Persiani (2005)**	Endogenous plasma levels of glucosamine were detected (10.4-204 ng/ml, with low intra-subject variability).<br><br>Glucosamine was rapidly absorbed after oral administration and its pharmacokinetics were linear in the dose range 750-1500 mg, but not at 3000 mg, where the plasma concentration-time profiles were less than expected based on dose-proportionality.<br><br>Plasma levels increased over 30-fold from baseline and peaked at about 10 mM with the standard 1500 mg once-daily dosage.<br><br>Glucosamine distributed to extravascular compartments and its plasma concentrations were still above baseline up to the last collection time.<br><br>Glucosamine elimination half-life was only tentatively estimated to average 15 h.
**PuigdellívolGrifell (2024)**	Participants showed a significant reduction in self-perceived pain after 3 and 6 months of treatment ($p < 0.0001$).<br><br>Lequesne Functional Index score was significantly reduced at 3 months and at 6 months of treatment ($p < 0.0001$).<br><br>The WOMAC index was significantly reduced after 3 and 6 months of treatment ($p < 0.0001$).<br><br>Significant reductions in WOMAC subdomains ($p < 0.0001$) were observed.
