# Supplementary material for: The Safety and Efficacy of Glucosamine and/or Chondroitin in Humans: A Systematic Review
Source: Nutrients. 2025 Jun 24;17(13):2093. doi: 10.3390/nu17132093 (PMC12250884; doi:10.3390/nu17132093)
Supplement: Supplementary file 1 [file nutrients-17-02093-s001.zip › Table S3.pdf]

Table S3. Glucosamine and/or Chondroitin Safety Outcomes Across Included Studies

RCT

Author Last Name (Year)	Safety Outcomes Included
**Alayat (2017)**	N/A
**Alhayek (2023)**	N/A
**Amalraj (2019)**	No significant changes in serum, urine, and hematological parameters in either group except for hs-CRP, which decreased significantly in both groups.<br><br>No serious adverse events were observed in either group.
**Armagan (2015)**	N/A
**Babur (2022)**	N/A
**Basak (2004)**	No adverse effects were reported or noted during the course of the study.
**Boeri (2024)**	N/A
**Cahlin (2011)**	Eight patients in the glucosamine group and 2 in the placebo group suspended the medication prematurely, after 14 days on average in both groups. Five patients in the glucosamine group ascribed this to unacceptable gastrointestinal side effects. The remaining 3 and the 2 patients in the placebo group reported other different adverse effects or did not specify any reason for their cessation.<br><br>After the follow-through of the trial, with 6 weeks of medication for the remaining 22 participants in the glucosamine group, a further 5 patients reported adverse reactions, all gastrointestinal. Of the 27 participants in the placebo group who completed the 6 weeks of medication, 3 reported adverse reactions, all also gastrointestinal. Therefore, a total of 10 patients in the glucosamine group and 3 in the placebo group who entered the trial reported gastrointestinal side effects.
**Catanzaro (2013)**	Both supplements were well tolerated and no allergic reactions were reported. In the glucosamine/chondroitin group, 36% (9/25) of patients complained of dyspepsia that was mild in intensity.
**Chopra (2013)**	There were no significant differences between the groups except for 12 patients (5 SGCG, 4 SGC and 3 glucosamine) who withdrew due to a study drug-related adverse event (AE) [pruritus, epigastric discomfort, nausea, oral ulcers and elevated serum glutamic pyruvic transaminase (SGPT)/alanine aminotransaminase (ALT)].<br><br>Clinically the adverse events (AEs) were predominantly mild and required only symptomatic treatment. None required hospitalization or any special/invasive intervention.<br><br>Twenty-six patients (11 SGCG, 4 glucosamine, 9 SGC and 2 celecoxib) in the study cohort showed an asymptomatic elevation in SGPT that was often accompanied by a proportionately smaller increase (<3 ULN) in other liver serum enzymes [aspartate aminotransaminase/serum glutamic oxaloacetic transaminase (SGOT) and alkaline phosphatase (ALP)] and all other normal liver functions including serum bilirubin and normal eosinophil count.
**Clegg (2006)**	Adverse events were generally mild and evenly distributed among the groups.

	Three serious adverse events were judged by the investigator to be related to study treatment: congestive heart failure (in a patient receiving combined glucosamine chondroitin treatment), stroke (in a patient receiving celecoxib), and chest pain (in a patient receiving glucosamine).<br><br>As compared with the placebo group, the celecoxib group had a significantly lower incidence of headache and nausea but had a nonsignificant but higher incidence of increased blood pressure.<br><br>Patients who received chondroitin sulfate had the highest incidence of musculoskeletal and connective-tissue events and the lowest incidence of vomiting.
**Cohen (2003)**	● Adverse events appeared to be of a minor nature and were equally distributed between the 2 groups. They included:<br>○ Upper respiratory tract infection and rash/skin numbness (3 in each group)<br>○ Muscle stiffness/soreness (1 in each group)<br>● Additional effects in the intervention group include one case each of nausea, fatigue, blurred vision, and gallstones.
**Cömert Kılıç (2021)**	No complications relating to the injections and use of supplements were observed during the treatment and follow-up periods.
**Crowley (2009)**	Overall, there were 58 adverse events noted in the subjects receiving G+C treatment, whereas, only 35 adverse events were observed in the UC-II group. In terms of severity, 60% of mild and 38% of moderate adverse events were experienced by subjects on G+C in comparison to 43% and 54% by subjects on UC-II. A higher number of subjects (23%) on G+C demonstrated adverse events possibly related to the product as compared to 11.4% of subjects on UC-II.<br><br>For UC-II the possible adverse events related to products were constipation and headaches (intermittently). For G+C the possible adverse events related to products were bloating, stomach pain, rash, water retention (edema around eyes and scars), hives on face and chest, and headache. However, there was no significant difference in the occurrence of adverse effects between the two treatment groups.
**Czajka (2018)**	No adverse events were reported.
**Damlar (2015)**	3 subjects were excluded due to blood aspiration and 1 due to vertigo owing to tramadol HCL. No other adverse effect was observed in either of the groups.
**Das (2000)**	The incidence of adverse events in the intervention group (17%) was less than the placebo group (19%). The most common adverse effects were gastrointestinal upset, which occurred in 15% of those in the control group and 21% of those in the placebo group.
**Eraslan (2015)**	None of the participants reported any side-effects related to supplementation and took any analgesic drug (such as NSAIDs) during the ingestion period of the study.
**Erhan (2012)**	One of the patients complained about temporary erythema that did not affect the patient's participation in the study.
**Esfandiari (2017)**	The mean IOP at the time of presentation was 12.4 ± 2.7 mmHg in glucosamine and 13 ± 2.8 mm Hg in the placebo group (p = 0.329). At month 1 the corresponding values were 12.6 ± 2.4 and 12.9 ± 2.4 mmHg (p = 0.868).<br><br>At 3 months follow-up, there was a statistically significant increase in IOP. 13.5 ± 2.3 for glucosamine and 13 ± 2.7 mm Hg for placebo (p = 0.002).<br><br>About 34.1% in treatment and 12.5% in the placebo group had clinically significant (defined as ≥ 2 mm Hg) rise in IOP at final follow-up (p = 0.023).<br><br>Mean age in those with significant rise in IOP was 66 vs 57.7 years in patients with < 2 mm Hg (p = 0.034).<br><br>There was no statistically significant difference between the two groups regarding the variance in ocular response analyzer parameters (p = 0.340).<br><br>Among those using glucosamine supplement, only one patient complained of GI upset during the first month of trial.

**Filipović (2022)**	In both treatment and control groups, patients dropped out due to dissatisfaction with the treatment, adverse gastrointestinal tract events (dyspepsia, nausea), hypertension, non-compliance, personal reasons, and/or medication unaffordability. Differences between groups were not assessed.
**Fransen (2015)**	During the 2-year follow-up, 126 (21%) participants withdrew from study treatment, only 34 (6%) for possibly-related medical events, with no major differences between the allocation groups.<br><br>Adverse events included blood glucose issues, cardiac problems, gastrointestinal/rash, and other medical events.
**Giordano (2009)**	The incidence of adverse events was greater in placebo (40.0%) compared to GS (36.7%).<br><br>Adverse events for glucosamine included musculoskeletal pain, flu syndrome, constipation, headache, diarrhea, itch, and heartburn.<br><br>Serious AEs occurred in 2 patients (6.7%) from the GS group and 1 patient (3.3%) from the placebo group.
**Gruenwald (2009)**	There was no statistically significant group difference in AEs (p = 0.521). 23 (12.9%) patients experienced AEs between the two groups, 12 in group A (glucosamine + omega-3s) and 9 in group B (glucosamine + other oils); 1 patient from both groups terminated the study). Four AEs were mild, eight were moderate, and nine were of stronger intensity.<br><br>All AEs were not serious. For 19 of the AEs (90.5%) a connection between the intake of the investigational product and the AEs was unlikely. In only two cases (itching, reddening, and small pimples on both feet and ankles, or stomach ache after intake; both group A) a connection was assessed as possible or probable. This resulted in a side effect rate of only 2.2% for the combination product.<br><br>The investigated clinical and biochemical parameters showed only marginal changes.<br><br>The global safety evaluation by the investigator and patients was assessed with over 93% commenting “very good” or ”good.”
**Herrero-Beaumont (2007)**	The number of adverse events reported during treatment was similar in all 3 groups: 89 with placebo, 96 with acetaminophen, and 95 with glucosamine sulfate.<br><br>The most frequent adverse events were of minor clinical significance and did not differ in frequency between groups. They included dyspepsia, abdominal pain, diarrhea, respiratory tract infections, gastroenteritis, coughing, headache, dizziness, back pain, neck pain, fall, and injury.<br><br>Routine laboratory examinations indicated that more patients in the acetaminophen group developed abnormalities in liver function (as reflected by levels of transaminases and gamma glutamyl transferase [GGT]); abnormalities were detected in 21 patients in the acetaminophen group versus 2 and 6 in the glucosamine sulfate and placebo groups, respectively.
**Hochberg (2008)**	Serious adverse events were seen in less than 4% of patients; specifically there were no deaths, nonfatal myocardial infarctions or GI bleeds. One patient taking celecoxib had a cerebrovascular accident and two patients had transient ischemic attack (one taking celecoxib and one glucosamine). Increased blood glucose levels were observed across all groups.
**Hochberg (2016)**	The overall proportion of subjects having at least one treatment-emergent AE were 51.0% (155/304) in the chondroitin sulfate plus glucosamine group and 50.5% (151/299) in the celecoxib group.<br><br>Three serious AEs were judged to be probably related to the study group, two in the chondroitin sulfate plus glucosamine group (Helicobacter pylori gastritis and allergic reaction) and one in the celecoxib group.
**Kanzaki (2012)**	Comparable numbers of subjects in both groups reported experiencing one or more adverse event(s) during the treatment<br><br>Frequent adverse events included cold symptoms, myalgia/muscle stiffness, arthralgia, gastric distress, and diarrhea.<br><br>There were no appreciable differences between the two groups in frequency or pattern of events.

	All of the self-reported adverse events were mild or intermediate in intensity and occurred only temporarily, and were judged by the investigator as medically unrelated to the treatment<br><br>Routine laboratory tests including those for glucose metabolism (blood glucose level, urinary glucose) did not show any great abnormalities in hematologic or metabolic functions in both groups during the 16-week treatment.
**Kanzaki (2015)**	All self-reported adverse events were transient and of mild or intermediate intensity.<br><br>No adverse effect of treatment was identified when results were analyzed on an individual-subject basis.
**Kawasaki (2008)**	The number of dropouts in the glucosamine, risedronate, and control groups was 17, 18, and 12, respectively. All dropouts in the risedronate group indicated the side effect of discomfort in the upper digestive tract as the reason for dropping out. In the glucosamine group, a sensation of abdominal distension was confirmed in one subject, and heartburn and gastric pain were observed in seven subjects of the risedronate group.
**Khanna (2020)**	N/A
**Kongtharvonskul (2016)**	The estimated risk of diarrhea after treatment was very close between the two groups (0.084 [0.051-0.136] for pCGS plus diacerein vs. 0.081 [0.048-0.138] for pCGS plus placebo) with an RR of 1.026 (95 % CI 0.559-1.885).<br><br>The risks of dyspepsia were also very similar (i.e., 0.030 [0.015, 0.059] vs 0.033 [0.017, 0.066]) with an RR of 0.910 (95 % CI 0.432-1.918).<br><br>The most common adverse event was abnormal urine (red or orange color), seen in 87.7% of patients receiving pCGS plus diacerein and in 66.2% of the patients receiving pCGS plus placebo.<br><br>For approximately one-third of the patients, the adverse events were related to the gastrointestinal system (diarrhea, gastritis, constipation, and nausea); however, there were no significant differences between groups in this regard. Approximately 10% of patients in both groups reported skin reactions.
**Kwoh (2014)**	There was no difference in reported side effects between the 2 groups. At the 24-week follow-up visit, 12.9% of the glucosamine group and 15.1% of the control group reported side effects (p = 0.6738).
**Leffler (1999)**	Side effect frequency was similar to that at baseline. No patients reported symptoms that required termination of the study. There were no effects on vital signs, occult blood testing, and hematologic parameters.
**Lomonte (2018)**	At least one adverse event was reported in 50 subjects, with 27 subjects (54.0%; 95% CI: 39.5 to 67.9%) in the GS/CS group and 23 subjects (46.0%; 95% CI: 32.1 to 60.5%) in the Cosamin DS group. There was no statistically significant difference between groups (p = 0.549)<br><br>The most commonly reported adverse events were flu and hyperglycemia, to which no relation with the treatment group was observed (p = 0.436 and p = 0.674, respectively)<br><br>Only one subject, in the GS/CS group, discontinued the study due to an adverse event (nausea).<br><br>Thirteen subjects reported adverse events potentially related to the study treatment, with 8 (16%; 95% CI: 7.6 to 29.7%) subjects in the GS/CS group and 5 (10%; 95% CI: 3.7 to 22.6%) subjects in the Cosamin DS group, not statistically different from each other (p = 0.552). The most commonly reported adverse reactions corresponded to upper gastrointestinal tract (dyspepsia, epigastralgy, nausea and heartburn) and changes in glucose counts.
**Lomonte (2021)**	A total of 1076 AEs were reported during the study, of which 504 occurred in the GS/CS group and 572 in the reference group, most of which were mild intensity.<br><br>The number of AEs considered related to the study treatment were 116 (23%) in the GS/CS group and 160 (28%) in the reference group.

	The most frequent treatment-related AEs were headache (12.7% in the GS/CS group, 14.4% in the reference group) and impaired glucose tolerance (2.9% in the GS/CS group, 5.5% in the reference group).
**Lubis (2017)**	N/A
**Lugo (2016)**	No clinical or statistically significant changes were reported for any of the hematologic, blood biochemistry, vital signs results, or urinalysis results.<br><br>A total of 45 AEs were reported during the 180-day study period: 9, placebo; 28, GC; and 8, UC-II. The majority (62%) of these occurred in the GC group.<br><br>15 of 45 events were classified as possibly related to supplementation, 14 of which belonged to the GC group and 1 to placebo. The 14 possible events linked to GC supplementation were primarily gastrointestinal in nature.
**Luo (2022)**	No statistically significant difference between the groups was observed in any safety parameters (vitals and biomarkers) during baseline ($p > 0.05$)<br><br>Both vitals and biomarkers did not change beyond the normal range over the study period.<br><br>None of the AEs observed during the study were related to the study products.
**Magrans-Courtney (2011)**	No adverse events reported.
**Mazières (2007)**	Seventy-five patients (49%) in the chondroitin sulphate group and 76 (49%) in the placebo group reported at least one treatment-emergent adverse event.<br><br>A total of 18 AEs possibly or probably related to treatment in 14 patients in the chondroitin sulphate group and 20 AEs in 16 patients in the placebo group were reported.<br><br>In both treatment groups, the majority (50%) of these AEs were related to gastro-intestinal troubles including dyspepsia, nausea, vomiting, abdominal pain and diarrhea.
**Messier (2007)**	No adverse events were reported except for hair loss in one participant in the GH/CS group.
**Michel (2005)**	There was no statistically significant difference in the frequency of any event between the two groups. Adverse events led to the withdrawal of 9 patients from each study group. Only 2 events were judged to be possibly related to CS (abdominal pain and nausea in 1 patient each).
**Minoretti (2024)**	Treatment-emergent adverse events in both the HA and Glc + CS groups were infrequent and did not show any significant differences between the two groups. No clinically relevant changes in laboratory values were observed in any of the study participants, regardless of their treatment group.
**Monfort (2017)**	N/A
**Morita (2018)**	One patient treated with the high-dose CS developed nausea. Other patients in both groups reported digestive discomfort, digestive irritation, or mild allergic reactions. In total, 20 and 13 patients dropped out in the low- and high-dose groups, respectively.
**Nakamura (2007)**	N/A
**Nakasone (2011)**	8 subjects in the test group and 6 subjects in the placebo group reported at least one intervention-associated adverse event.<br><br>All adverse events were of mild intensity and were judged by the medical investigator as unrelated to the intervention.<br><br>Adverse events most frequently reported from both the test and placebo subjects were respiratory symptoms (asthma, sore throat, cough, rhinorrhea, malaise and/or fever) and pain (hip, tooth or head).

**Nash (2018)**	N/A
**Navarro (2015)**	N/A
**Navarro (2019)**	No adverse events were reported.
**Navarro (2020)**	N/A
**Nguyen (2001)**	N/A
**Nieman (2013)**	N/A
**Pavelká (2002)**	There were no differences between groups in safety. 64% of patients receiving placebo and 66% receiving glucosamine sulfate reported at least 1 adverse event during the 3 years of study. The most frequently reported complaints were attributable to the gastrointestinal tract and liver systems and consisted predominantly of transient episodes of abdominal pain and dyspeptic symptoms.
**Pelletier (2016)**	A total of 78 patients (80.4 %) in the CS group and 79 patients (81.4 %) in the celecoxib group reported at least one AE during the study.<br><br>The only AE that patients in the CS group experienced significantly more of than patients in the celecoxib group was back pain.<br><br>No death occurred during the study. There were no significant differences between groups for serious adverse events (SAE).
**Petersen (2011)**	N/A
**Provenza (2015)**	There were 1,824 reported adverse events evenly distributed that were generally considered mild corresponding to 619, 589, and 616 in GI, GII, and GIII, respectively.<br><br>All but one of the seven (0.38 %) reported serious adverse events were considered as not related to study medications. Mesenteric occlusion had a fatal event, and was considered possibly related to study drug.
**Puente (2017)**	Vital signs and blood parameters were not affected by the treatments, and individual values remained within normal ranges.<br><br>Three subjects (1 in D-002 group, 2 in GS/SC group) referred some AE during the trial: urinary infection (D-002), heartburn (GS/SC) and stomach pain (GS/SC)
**Railhac (2012)**	Treatment-emergent adverse event (TEAE) incidence was higher in the Structum group: 18 out of 25 patients (72%) versus 13 out of 23 (56%) in the placebo group.<br><br>As expected in such a patient population, musculoskeletal and connective tissue disorders were the most frequent TEAEs, namely reported by 40% of patients under Structum and 17% under placebo. They were followed by infections and infestations, reported by 24% of patients under Structum and 30% of patients under placebo, and 20% and 22% of patients, respectively, experienced gastrointestinal disorders.<br><br>Most AEs were of mild or moderate intensity.<br><br>Only two patients in the Structum group and one in the placebo group experienced AEs which were considered possibly, or probably, related to the study drug.
**Reginster (2001)**	93% with placebo and 94% with glucosamine sulphate reported at least one adverse event. There were no substantial differences between groups in frequency or pattern of events. Most of them were transient and of mild to moderate severity. In about half the cases, events were referred to the gastrointestinal system (mainly including abdominal pain and disturbed defecation) and may be also referred to the rescue medication, without differences between groups. Among adverse events leading to patient dropout, few single episodes were serious and were all judged as unrelated to the study treatment.

	Routine laboratory tests did not show any great abnormalities in system organs or metabolic functions in the two groups during the study. There was no change in glycemic homoeostasis, with fasting plasma glucose concentrations decreasing slightly in the glucosamine sulphate group.
**Rindone (2000)**	Seventeen patients (34%) taking glucosamine experienced side effects compared with 11 (23%) taking placebo. Most side effects in both groups were mild and self-limiting; side effects included loose stools, nausea, heartburn, and headache.<br><br>2 participants taking glucosamine and 4 taking placebo withdrew from the study due to adverse side effects, but the difference between groups was not statistically significant (p = 0.67).
**Roman-Blas (2017)**	A small number of dropouts related to AEs was reported in both groups. The incidence of dropout was higher in the CS/GS combination therapy group, and reasons for dropping out mainly consisted of abdominal symptoms such as diarrhea, upper abdominal pain, and constipation.
**Rondanelli (2019)**	No significant side effects were observed in the experimental group.
**Rondanelli (2020)**	N/A
**Sawitzke (2008)**	N/A
**Scroggie (2003)**	Hemoglobin A1c:<br>● Posttreatment hemoglobin A1c concentrations were not significantly different between groups (p = 0.20), nor were there any significant differences within groups before and after treatment.<br><br>Adverse effects:<br>● There were 4 withdrawals from the glucosamine-treated group. Three were related to comorbidities (myocardial infarction, congestive heart failure, and atrial fibrillation) and 1 to a possible adverse reaction (excessive flatus).<br>● No other patient reported any adverse effects of glucosamine therapy, and no patient had any change in their diabetes management.
**Sevimli (2020)**	N/A
**Sterzi (2016)**	No adverse events were reported during the study.
**Thomas (2021)**	Both the CGM and standard drug treatments were well tolerated without exhibiting any serious adverse events.<br><br>There were no significant differences observed in the clinical parameters including lipid profile, renal (creatinine and BUN), and hepatic function tests (SGOT and SGPT) from the baseline to end of the study, in both the study groups, indicating the safety of the supplements used at the particular dosage and duration.
**Tío (2017)**	N/A
**Truong (2019)**	At week 12, there were no significant abnormal changes in hematology and clinical chemistry compared to baseline. There were no remarkable changes in the complete blood counts, differential white blood cell counts, hepatic and renal functions, lipid profiles, BMI, and vitals in all three groups.<br><br>Three adverse events were reported in Group A: facial edema (n = 1), headache (n = 1), and hypertension (n = 1). In all cases, the adverse events were confirmed as unrelated to the investigational products.
**Tsuji (2016)**	No adverse events were reported.
**Uebelhart (2004)**	Only minor adverse events were observed in both groups. Those possibly related to the treatment were gastrointestinal (epigastralgia, pyrosis and nausea) and occurred in both CS and PBO groups with a frequency of four and six events, respectively.

	The global assessment of tolerability expressed by both the patients and the physicians was very similar and no difference was observed between the two groups except for a significantly better tolerability score for the CS group after 1 month of treatment.
**Usha (2004)**	Except for mild gastrointestinal discomfort, no patient had any serious side effect. The main adverse event was diarrhea, occurring in > 5% patients, and this was more common in the Glu group.<br><br>None of the patients discontinued therapy due to any adverse drug reaction.
**Velickovic (2023)**	Nausea and dyspepsia were the most commonly reported adverse events; however, there was no statistically significant difference between the groups.
**Vicenzino (2019)**	Skin breakdown (13, 24%) and skin irritation (28, 51%) were present in significantly more ($p < 0.05$) of those who wore the knee guard device, with only 1 (2%) of the NSAID gel group reporting skin break down
**Wang (2021)**	N/A
**Wang (2021)**	22 (27.2%) subjects had at least 1 AE (12 [30.8%] subjects in the A + HA group, 10 [23.8%] subjects in the placebo group), and none of them was treatment-related. Upper abdominal pain was the most frequent reported AE in both groups (2 [5.1%] subjects in the A + HA group, 2 [4.8%] subjects in the placebo group). There was no AE that led to study product discontinuation or study discontinuation.
**Wildi (2011)**	Non-serious adverse events were equally distributed within the two treatment groups except for infections and skin disorders, which were more frequent in the CS group.
**Wilkens (2010)**	Mild adverse events were reported in 40 patients in the glucosamine group and 46 in the placebo group ($p = 0.48$).
**Xia (2016)**	AEs reported were generally mild and evenly distributed among groups. The overall incidence of adverse events has no statistically significant differences ($p = 0.479$) between the HA group (10%) and GS group (6%).<br><br>In the HA group, the five reported AEs were local reactions in the target knee, and the AEs resolved without treatment in 1 week. The three reported AEs of GS included mainly gastrointestinal disorders. No AEs occurred in the placebo group.
**Yue (2012)**	Adverse events reported were generally mild and evenly distributed among groups. A total of 23 adverse events were reported in 18 patients. No serious adverse events were reported.<br><br>In general, notable abnormal laboratory test results did not follow any clinically relevant pattern in any treatment group.
**Zegels (2013)**	No statistically significant difference was demonstrated between the three treatment groups in the mean number of adverse events or in the number of subjects in each group with at least one adverse event.
**Zenk (2002)**	14 adverse effects were reported in all 3 groups. Two reports of nausea were considered possibly related to use of glucosamine.<br><br>No long-term adverse events of any treatment were reported.
**Zhang (2010)**	18.2% with placebo and 16.1% with chondroitin sulfate and glucosamine reported having an adverse event during the 8-month follow-up, and there was no difference between the two groups ($p = 0.95$).<br><br>Adverse events included abdominal pain and headache. Two of 40 patients in each group withdrew due to adverse events, and there were no significant differences.
**Zhang (2021)**	The incidence rate of adverse reactions in the celecoxib group was 15%, and that in the ComG was 4.41%, with statistically evident difference ($p < 0.01$).

Cohort

First Author (Year)	Safety
**Bell (2012)**	5362 deaths were recorded, for a death rate of 10.2 deaths per 1000 person-years. 20.2% of participants ever used glucosamine, and about two-thirds of glucosamine supplements used by participants included chondroitin and about 10% contained methylsulfonylmethane (MSM). Current (baseline) glucosamine supplement use of any formulation was associated with a decreased risk of total mortality compared to never use (HR 0.82, 95% CI 0.75–0.90). The adjusted hazard ratios were HR 0.86 (95% CI 0.78–0.96) for chondroitin, 0.78 (95% CI 0.67–0.91) for glucosamine without chondroitin, and non-significant for MSM.<br><br>Glucosamine<br>● The study observed a non-significant reduced risk of mortality from total cardiovascular disease (HR 0.88 95% CI 0.74–1.06), with similar results for the subcategory of ischemic heart disease. There was a significant 13% decreased risk of death from cancer among current users of glucosamine (HR 0.87 95% CI 0.76–0.98). The study also observed a risk reduction for current use of glucosamine and all other causes of death other than cardiovascular disease and cancer (HR 0.74 95% CI 0.56–0.81), and within those other causes, a strong specific reduction in mortality due to respiratory disease (HR 0.59 95% CI 0.41–0.83), the subcategory with the most deaths. The hazard ratio for current glucosamine use with total mortality was 0.80 (95% CI 0.72–0.89), with CVD mortality was 0.84 (95% CI 0.6–1.03), with cancer mortality was 0.83 (95% CI 0.72–0.96) and with other causes of mortality was 0.75 (95% CI 0.57–0.96).<br><br>Chondroitin<br>● Results for current use of chondroitin with mortality were similar to those for current use of glucosamine, except there was no association between current chondroitin use and total cancer mortality (HR 0.94 95% CI 0.81–1.10).
**Bhimani (2023)**	4908 persons died over a median of 8.1 years of follow-up (max: 16.75 years), including 1086 deaths due to cancer, 840 deaths due to cardiovascular disease, and 2969 deaths due to other causes. Of these deaths, 12.2% were among those who regularly used any glucosamine, and 8.8% in those who did not.<br><br>Despite glucosamine use seemingly inversely associated with mortality in minimally adjusted models (hazard ratio (HR): 0.70; 95% CI: 0.59-0.84), following multivariable adjustment, no association was observed (HR: 1.02; 95% CI: 0.85-1.23).<br><br>Similar results were observed for chondroitin and joint use of glucosamine and chondroitin, with no association observed after multivariable adjustment (chondroitin: HR: 1.05; 95% CI: 0.87-1.27; glucosamine chondroitin: HR: 1.05; 95% CI: 0.86-1.28; or methylsulfonylmethane (MSM): HR: 1.02; 95% CI: 0.74-1.40).<br><br>A significant inverse association was found after regular use of glucosamine/chondroitin for both cardiovascular mortality and other cause mortality rate in the minimally adjusted model. The association did not remain statistically significant for the cardiovascular mortality (glucosamine: HR 0.72; 95% CI: 0.45-1.14, chondroitin: HR: 0.75; 95% CI: 0.46-1.21) and was null for other cause mortality rate (glucosamine: HR: 1.00; 95% CI: 0.79-1.25, chondroitin: HR: 0.98; 95% CI: 0.78-1.23).<br><br>An HR of 1.08 (95% CI: 0.90-1.31) was observed in the early years of follow-up for glucosamine, compared with 0.86 (95% CI: 0.57-1.29) in the later years of follow-up. An HR of 1.15 (95% CI: 0.93-1.42) was observed for chondroitin in early follow-up, compared with 0.79 (95% CI: 0.53-1.19) in later follow-up. For both glucosamine and chondroitin, there was no evidence of interaction by year of study entry (p-interaction: 0.98 and p-interaction: 0.99, respectively).
**Cho (2019)**	N/A
**Cho (2023)**	Main analysis:<br>● Genetically predicted chondroitin and glucosamine intake were significantly associated with lower eGFR (p = 2 x 10^-4 and p = 6 x 10^-12,

	respectively). In contrast, genetically predicted vitamin/mineral supplement intake was significantly associated with a higher eGFR (p = 1 × 10^−25).<br>Validation analysis:<br>● Chondroitin and vitamin/mineral supplement intake showed results concordant with those of the main analyses. In other words, regular chondroitin intake was associated with a lower eGFR, whereas regular vitamin/mineral intake and kidney function were associated with a higher eGFR in different outcome datasets (p = 4 x 10^-31 and p = 4 x 10^-19, respectively). Regarding glucosamine intake, however, the results of the main analyses were not replicated when analyzed using the log-eGFRcr meta-analysis summary statistics provided by the CKDGen and UKB (p = 3 x 10^-6).<br>Sensitivity analysis for chondroitin intake:<br>● Regarding the MR results for chondroitin intake and kidney function, an MR-Egger intercept p < 0.05 was observed, indicating potential pleiotropy.
**Hotaling (2011)**	Six commonly used anti-inflammatory supplements (glucosamine, chondroitin, saw palmetto, ginko biloba, garlic and fish oil) did not show any statistically significant reduction in incident UC in the multivariate model (HR for high use glucosamine vs. non-use 0.99 CI 0.65,1.50, p-trend = 0.432; HR for high use chondroitin vs. non-use 1.06 CI 0.65,1.73, p-trend = 0.622; HR for high use fish oil vs. non-use 0.87 CI 0.50,1.51, p-trend = 0.497)
**Kantor (2016)**	672 CRC cases occurred.<br><br>Associations were comparable across cohorts, with a RR of 0.79 (95% CI: 0.63-1.00) observed for any use of glucosamine and a RR of 0.77 (95% CI: 0.59-1.01) observed for any use of chondroitin.<br><br>Use of glucosamine in the absence of chondroitin was not associated with risk of CRC, whereas use of glucosamine + chondroitin was significantly associated with risk (RR: 0.77; 95% CI: 0.58-0.999).<br><br>The association between use of glucosamine + chondroitin and risk of CRC did not change markedly when accounting for change in exposure status over follow-up (RR: 0.75; 95% CI: 0.58-0.96), nor did the association significantly vary by sex, aspirin use, body mass index, or physical activity.<br><br>The association was comparable for cancers of the colon and rectum.
**King (2020)**	Of the participants, 658 (3.94%) had been taking glucosamine/chondroitin for a year or longer.<br><br>During follow-up (median, 107 months), there were 3366 total deaths (20.17%); 674 (20.02%) were due to CVD. Respondents taking glucosamine/chondroitin were less likely to have CVD mortality (hazard ratio [HR] = 0.51; 95% CI, 0.28-0.92).<br><br>After controlling for age, use was associated with a 39% reduction in all-cause (HR = 0.61; 95% CI, 0.49-0.77) and 65% reduction (HR = 0.35; 95% CI, 0.20-0.61) in CVD mortality.<br><br>Multivariable-adjusted HR showed that the association was maintained after adjustment for age, sex, race, education, smoking status, and physical activity (all-cause mortality, HR = 0.73; 95% CI, 0.57-0.93; CVD mortality, HR = 0.42; 95% CI, 0.23-0.75).
**Li (2023)**	84,895 (18.9%) participants reported regular glucosamine use at baseline.<br><br>During a median of 12.5 years follow-up, glucosamine use was significantly associated with an increased risk of overall cancer [HR, 1.04; 95% confidence interval (CI), 1.01-1.06], skin cancer (HR, 1.11; 95% CI, 1.07-1.15), and prostate cancer (HR, 1.07; 95% CI, 1.01-1.13), and with a reduced risk of lung cancer (HR, 0.88; 95% CI, 0.79-0.97) after adjusting for potential confounders.<br><br>Statistical interaction was observed for gender, age, and education for the association of glucosamine use with overall cancer risk. These results remained unchanged in the sensitivity analyses.

**Lila (2023)**	307 AEs were reported in 190 (17.2%) participants. The most common AEs for system organ classes of MedDRA were: "Musculoskeletal and connective tissue disorders" (5.3%), "Gastrointestinal disorders" (4.7%), and "Infections and infestations" (4.4%). The most common AEs by the Preferred Term included OA (worsening of the main diagnosis) (1.2%), upper abdominal pain (1.3%), upper respiratory tract infections (2.3%), and headache (1.3%). In most patients, AEs were mild [108 patients (9.8%)] or moderate [78 patients (7.1%)].<br><br>AEs considered by the investigators as "related to the study product" were reported for 31 (2.8%) patients (a total of 33 AEs) and mainly included gastrointestinal disorders [25 AEs in 24 (2.2%) patients]. The most common AE was upper abdominal pain [12 (1.1%) patients].<br><br>Serious AEs (SAEs) were reported in 14 (1.3%) patients, including atrial fibrillation (2 patients), cholecystectomy, coronary bypass, hip arthroplasty, knee arthroplasty, hemorrhoids, ischemic thrombotic stroke, radicular pain syndrome, cholelithiasis, foot fracture, and myositis (one patient for each SAE). Additionally, one patient had several cardiac-related SAEs (atrial fibrillation, angina pectoris, chronic heart failure, ischemic cardiomyopathy, myocardial ischemia, and tachycardia) and another patient had gastrointestinal disorders (gastric ulcer and gastric ulcer bleeding, which was fatal).
**Ma (2019)**	The study recorded 10204 incident CVD events, 3060 CVD deaths, 5745 incident CHD events, and 3263 incident stroke events. In the age adjusted analyses, the study found significant inverse associations between glucosamine use and risk of total CVD events, CVD death, CHD, and stroke (all $p < 0.001$)<br><br>In the multivariable adjusted analyses, the hazard ratios associated with glucosamine use were 0.85 (95% confidence interval 0.80 to 0.90; $p < 0.001$) for total CVD events; 0.78 (0.70 to 0.87; $p < 0.001$) for CVD death; 0.82 (0.76 to 0.88; $p < 0.001$) for CHD; and 0.91 (0.83 to 1.00; $p = 0.04$) for stroke.<br><br>The study analyzed the relations between glucosamine use and subtypes of CHD and stroke. For CHD, glucosamine use was associated with significantly lower risks of non-fatal CHD (hazard ratio 0.84, 95% confidence interval 0.77 to 0.91; $p < 0.001$) and fatal CHD (0.70, 0.59 to 0.85; $p < 0.001$). For stroke, glucosamine use was associated with a marginally significantly lower risk of non-fatal stroke (0.91, 0.82 to 1.01; $p = 0.08$), but it was not associated with risk of fatal stroke (0.87, 0.68 to 1.13; $p = 0.30$). There was no significant inverse association between glucosamine use and risk of ischemic stroke (0.92, 0.80 to 1.03; $p = 0.14$) or hemorrhagic stroke (0.89, 0.75 to 1.07; $p = 0.21$)
**Pocobelli (2010)**	None of the vitamin or mineral 10-y average intakes were associated with total mortality.<br><br>Among the nonvitamin-nonmineral supplements, only glucosamine and chondroitin were associated with total mortality. The hazard ratio (HR) when persons with a high intake of supplements (4 d/wk for 3 y) compared with nonusers was 0.83 (95% CI: 0.72, 0.97; p for trend = 0.009) for glucosamine and 0.83 (95% CI: 0.69, 1.00; p for trend = 0.011) for chondroitin.
**Raynauld (2016)**	N/A
**Roubille (2015)**	N/A
**Rovati (2016)**	N/A
**Yang (2015)**	N/A
**Yu (2022)**	During a median follow-up of 6.13 years, 64,600 new-onset CVD, 26,530 CHD, and 17,832 stroke events occurred.<br><br>Glucosamine usage was significantly associated with CVD (HR: 1.10; 95% CI: 1.08-1.11) and CHD (HR: 1.12; 95% CI: 1.09-1.15), but not with stroke (HR: 1.03; 95% CI: 0.99-1.06).<br><br>The highest CVD risk was shown in the adherent group (HR: 1.68; 95% CI: 1.59-1.78), followed by the partially adherent group (HR: 1.26, 95% CI: 1.22-1.30), and the non-adherent group (HR: 1.03; 95% CI: 1.02-1.05), with a significant dose-response relationship (p-trend < 0.001).
**Zheng (2023)**	Associations of glucosamine use with incident dementia:

	● A statistically significant inverse relationship was found between glucosamine use and risk for all-cause dementia (HR 0.81; 95% CI 0.73-0.90), Alzheimer's disease (AD) (HR 0.78; 95% CI 0.65-0.92), and vascular dementia (HR 0.68; 95% CI 0.54-0.87).<br>● The hazard ratios of glucosamine users in multivariable-adjusted models were 0.84 (95% CI 0.75 to 0.93) for all-cause dementia; 0.83 (95% CI 0.71 to 0.98) for AD; and 0.74 (95% CI 0.58 to 0.95) for vascular dementia.<br>Mendelian randomization:<br>● Genetically determined regular glucosamine use was associated with a decreased risk for all-cause dementia [Inverse variance-weighted (IVW) odds ratio, 0.85; 95% CI 0.76 to 0.95)], AD (IVW odds ratio, 0.85; 95% CI 0.78 to 0.93) and vascular dementia (IVW odds ratio, 0.64; 95% CI 0.42 to 0.96).<br>The genetic liabilities for regular glucosamine, vitamin, chondroitin use, and osteoarthritis were evaluated. Use of glucosamine continued to have a significant effect on all-cause dementia (IVW odds ratio, 0.88; 95% CI, 0.8-0.95; p < 0.001), AD (IVW odds ratio 0.78; 95% CI, 0.72-0.85; p < 0.001) and vascular dementia (IVW odds ratio, 0.73; 95% CI, 0.57-0.94; p < 0.001).
**Zheng (2023)**	Genetically determined regular use of glucosamine is associated with a reduced risk of HF, with an inverse variance-weight (IVW) odds ratio of 0.91 (95% CI, 0.85 to 0.97).<br><br>In the MVMR analysis, the study evaluated the joint effect of genetic liabilities for regular use of glucosamine, vitamin, and chondroitin. The results showed that the use of glucosamine remained significantly associated with a decreased risk of HF (IVW odds ratio, 0.92; 95% CI, 0.87 to 0.96; p < 0.001).<br><br>The study identified that glucosamine intake was associated with decreased CHD (IVW β = -0.08; 95% CI, -1.14 to -0.03; p = 0.003) and stroke (IVW β = -0.06; 95% CI, -0.11 to -6.0 10^-3; p = 0.030). In the second stage, we used genetic instruments for CHD and stroke, to analyze the mediators' causal influence on HF risk. We found causal evidence for effects of CHD (IVW β = 0.12; 95% CI, 0.06 to 0.19; p < 0.001) and stroke (IVW β = 0.24; 95% CI, 0.07 to 0.41; p = 0.006) on HF after adjusting for glucosamine intake.<br><br>The study identified that the mediation effect of CHD was -1.03 x 10^-2 (95% CI, -2.03 x 10^-2 to -1.12 x 10^-3; p = 0.021) with a mediated proportion of 10.5% (95% CI, 7.6% to 13.4%) and the mediation effect of stroke was -1.40 x 10^-2 (95% CI, -2.71 x 10^-2 to -6.82 x 10^-4; p = 0.035) with a mediated proportion of 14.4% (95% CI, 10.8% to 18.0%). The two-mediator combination accounted for 22.7% (95% CI, 17.2% to 28.2%) of the effect of glucosamine intake.
**Zhou (2023)**	Overall, habitual glucosamine use was significantly associated with a lower risk of incident vascular dementia (adjusted HR, 0.82; 95%CI, 0.70-0.96), but not significantly associated with incident Alzheimer's disease (adjusted HR, 1.02; 95%CI, 0.92-1.14) and incident frontotemporal dementia (adjusted HR, 0.95; 95%CI, 0.63-1.43).<br><br>The inverse association between habitual glucosamine use and incident vascular dementia was more pronounced in participants with concomitant supplementation of calcium (P-interaction = 0.011), and those without concomitant supplementation of zinc (p-interaction = 0.018).<br><br>APOE ε4 dosage and baseline cognitive function did not significantly modify the relationships of glucosamine use with incident vascular dementia or Alzheimer's disease (All p-interactions > 0.05).

Non-RCT Experimental

**First Author (Year)**	**Safety**
**Belcaro (2014)**	No adverse events were reported.
**Greenlee (2013)**	Most commonly reported side effects were grade 1 headache (28%), grade 1 dyspepsia (15%), and grade 1 nausea (17%).

**Klein (2003)**	All 30 patients experienced varying degrees of postinjection pain.<br><br>One patient developed increased leg pains for 2 months after the procedure, which completely resolved.
**Kubový (2012)**	N/A
**Matsuno (2009)**	No adverse reactions were apparent in either the RA or OA patients.
**Muftic (2024)**	N/A
**Persiani (2007)**	No safety issues reported in the study.
**Puigdellivol (2019)**	Patients (2.3%) reported mild adverse events in the form of gastric dyspepsia (1 patient, in visit after 3 months) and nausea (2 patients, one after 3 months and the other after 6 months).<br><br>No major adverse events were noted.
**Shankland (1998)**	Gastrointestinal upset was the only reported side-effect of taking these supplements, reported by only two individuals. These symptoms subsided immediately when the supplements were discontinued.
**Tokhiriyon (2019)**	N/A
**Vreju (2019)**	N/A
**Weimann (2001)**	If incubated with platelets and sera from HIT patients, glucosamine sulfate did not cause a serotonin release of >20% with the sera tested at any of the concentrations used.<br><br>All sera showed positive results with PF4/heparin complex ELISA; however, no enhanced binding to PF4/glucosamine sulfate complexes was found over the whole range of glucosamine sulfate concentrations tested.<br><br>Preincubation of platelets with glucosamine sulfate at any given concentration had no effect on platelet activation by HIT antibodies in the presence of heparin (0.2 IU/ml).

Cross-Sectional

First Author (Year)	Safety
**Ayhan (2024)**	No severe side effects were observed related to the study medication.
**Blakeley (2002)**	Subjects did not report any instances of adverse effects resulting from taking GLS along with other natural health products (NHPs) or conventional medications.<br><br>Only three individuals (5%) reported any problems or complications associated with taking GLS. These included dyspepsia, headache, nausea/vomiting, gas, and skin rash.
**Issa (2021**	Of those who completed the study (n = 79), one patient reported mild skin irritation.

**Kantor (2012)**	N/A
**Kantor (2014)**	N/A
**Lapane (2012)**	N/A

Case-Control

First Author (Year)	Safety
**Dorais (2018)**	Exposure to narcotics and glucosamine/chondroitin sulfate was relatively similar between cases and controls.<br><br>For glucosamine/chondroitin sulfate exposure, 23.0% of cases and 23.1% of the controls had exposure between 1-79% and 31.7% of cases and 31.1% of controls had exposure ≥ 80%.<br><br>Exposure = the percentage (%) of all available follow ups at which the subjects reported currently using oral OA therapies.<br><br>No significant association was found between the occurrence of knee replacement (KR) and exposure to any of the oral OA therapies (including glucosamine/chondroitin) within the 3 years prior to KR.<br>● For exposure 1-79% to glucosamine/chondroitin sulfate: p = 0.573, CI 0.50-1.48, OR 0.85<br>● For exposure ≥ 80%: p = 0.236, CI 0.43-1.23, OR 0.73
**Hsu (2019)**	N/A
**Ibáñez-Sanz (2020)**	Chondroitin sulfate and glucosamine consumption and colorectal cancer risk:<br>● Ever use of chondroitin sulfate was associated with a nonsignificant 4% reduced risk of colorectal cancer (OR: 0.96; 95% CI, 0.91-1.01). This association was stronger for past exposures (OR for exposure more than 3 years before the index date: 0.92; 95% CI, 0.86-0.98).<br>● There was a slight dose-response trend with longer duration of use (OR for >36 months: 0.92; 95% CI, 0.83-1.02) and cumulative daily defined doses (DDDs) of exposure (OR for >240 DDDs: 0.91; 95% CI, 0.84-0.99). In the stratified analysis by NSAIDs, a paradoxical opposite association was observed: users of only chondroitin sulfate had a higher risk of colorectal cancer (OR: 1.16; 95% CI, 1.00-1.34), while among NSAID users, chondroitin sulfate was negatively associated with colorectal cancer risk (OR: 0.93; 95% CI, 0.88-0.98, p interaction = 0.0037)<br>● Ever use of glucosamine was associated with a significant 8% reduced risk of chondroitin sulfate (OR: 0.92; 95% CI, 0.87-0.97). This risk was similar for recent or past exposures. There was a dose-response relationship for duration, and more evident for cumulative DDDs (OR for >240 DDDs: 0.85; 95% CI, 0.77-0.94). In the stratified analysis by NSAID use, an effect modification was not observed (p interaction = 0.52).<br>● The stratified analysis showed that there was an increased protective association with concurrent use of chondroitin sulfate and glucosamine (OR: 0.83; 95% CI, 0.70-0.98) suggesting a possible synergistic effect.<br>● The protective association of chondroitin sulfate was stronger among men (OR: 0.90; 95% CI, 0.84-0.97) than women (OR: 0.98; 95% CI, 0.92-1.02; p interaction = 0.053), but no effect modification was observed in glucosamine users (p = 0.15).<br>NSAID consumption and colorectal cancer risk:<br>● Ever use on any NSAID, with a frequency of 35% among controls, had a protective association with colorectal cancer, with an 11% risk reduction (OR: 0.89; 95% CI, 0.87-0.92). This protective association was higher with longer exposure (OR for >10 years: 0.79; 95% CI, 0.76-0.84) and with higher cumulative dose (OR for >1,800 DDD: 0.80; 95% CI, 0.77-0.84).<br>● Chondroitin sulfate use was more frequent among cases than controls (OR: 1.10; 95% CI, 0.90-1.33). Similarly, there was a lack of protective

	association for glucosamine in the small subgroup that consumed it without NSAIDs (OR: 0.96; 95% CI, 0.87-1.07). The use of chondroitin sulfate or glucosamine simultaneously to NSAID showed, however, an increased protective association compared with the use of either drug alone (OR: 0.80; 95% CI, 0.72-0.88). Both the combination of NSAID + chondroitin sulfate or NSAID + glucosamine had a similar OR = 0.82 (95% CI, 0.76-0.88).
**Mazzucchelli (2021)**	89 cases (0.38%) and 757 controls (0.64%) were current users of CS at index date, yielding an AOR of 0.57 (95% CI: 0.46-0.72). The reduced risk of AMI among current users was observed in both short-term (< 365 days, AOR = 0.58; 95% CI: 0.45-0.75) and long-term users (> 364 days AOR = 0.56; 95% CI:0.36-0.87), in both sexes (men, AOR = 0.52; 95% CI:0.38-0.70; women, AOR = 0.65; 95% CI:0.46-0.91), in individuals over or under 70 years of age (AOR = 0.54; 95% CI:0.38-0.77, and AOR = 0.61; 95% CI:0.45-0.82, respectively) and in individuals at intermediate (AOR = 0.65; 95% CI:0.48-0.91) and high cardiovascular risk (AOR = 0.48; 95% CI:0.27-0.83), but not in those at low risk (AOR = 1.11; 95% CI:0.48-2.56).<br><br>In contrast, the current use of glucosamine was not associated with either increased or decreased risk of AMI (AOR = 0.86; 95%CI:0.66-1.08).
**Mazzucchelli (2022)**	106 cases (0.76%) and 803 controls (1.16%) were current users of glucosamine or CS at index date, yielding an AOR of 0.66 (95% CI: 0.54-0.82) (for glucosamine, AOR: 0.55; 95% CI: 0.39-0.77; and for CS, AOR: 0.77; 95% CI: 0.60-0.99).<br><br>The reduced risk of ischemic stroke among current users was observed in both sexes (men, AOR: 0.69; 95% CI: 0.49-0.98; women, AOR: 0.65; 95% CI: 0.50-0.85), in individuals above and below 70 years of age (AOR: 0.69; 95% CI: 0.53-0.89 and AOR: 0.59; 95% CI: 0.41-0.85, respectively), in individuals with vascular risk factors (AOR: 0.53; 95% CI: 0.39-0.74) and among current/recent users of nonsteroidal anti-inflammatory drugs (NSAIDs) (AOR: 0.71; 95% CI: 0.55-0.92).<br><br>Regarding duration, the reduced risk was observed in short-term users (< 365 days, AOR: 0.61; 95% CI: 0.48-0.78) but became nonsignificant in long-term users (> 364 days AOR: 0.86; 95% CI: 0.57-1.31).
**Pontes (2018)**	Crude risks of Acute Coronary Events (ACE) showed significant associations for all exposures except for aceclofenac, celecoxib and etoricoxib, and selective cox-2 inhibitors as a group; crude odds ratios (OR) were of protective sign for glucosamine, chondroitin sulphate and SYSADOAS as a group.<br><br>Individual active principles within the opioid group did not show significant risks in the adjusted model. Adjusted models did not show significant associations for COX-2 selective NSAIDs (p = 0.720), topical NSAIDs (p = 0.539), glucosamine (p = 0.904), chondroitin sulphate (p = 0.313), paracetamol (p = 0.058), nor metamizole (p 0.871).

Case Series/Report

First Author (Year)	Safety
**Cerda (2013)**	Twenty-three patients (15.2% of the total) acknowledged having consumed products containing glucosamine (6 patients), chondroitin sulfate (16 patients) or both (1 patient).<br><br>In 21 out of the 23 patients it could not be established whether the liver had sustained drug-induced damage since no elevation in aminotransferase above the usual values was observed in association with the administration of glucosamine or chondroitin sulfate.<br><br>A relationship between an elevation in transaminases and product consumption was detected in 2 cases, both of which had taken glucosamine. One patient was a 71-year-old woman with chronic hepatitis C who had taken glucosamine sulfate during one year and presented an elevation in aminotransferases of 5 to 7-fold greater than the normal values during this treatment. Serum transaminases returned to the usual values after treatment discontinuation.<br><br>The second case was a 77-year-old woman with chronic hepatitis C who had taken glucosamine for 3 months in 1977 and had presented an allergic cutaneous reaction attributed to the drug. At that time the transaminase values rose 4-fold above normal. In the follow-up liver tests taken in June 2011, a minimum

	elevation of alanine aminotransferase similar to previous analyses was observed.
**Chu (2023)**	Within 3 hours of the first dose of glucosamine sulfate and chondroitin sulfate, the patient noticed the appearance of an itchy rash on her torso and legs. Over the next few hours, the rash spread across her entire body, accompanied by facial swelling and stiffness in the chest. The patient also experienced nausea, dizziness, and vomiting.
**Hoban (2020)**	71.85% of cases (n = 263) were found to have hypersensitivity reactions. Of these 263 cases, 92 cases were classified as mild (eg, pruritus, urticaria and lip edema), 128 cases classified as moderate (such as dyspnea, nausea and abdominal pain), and 43 cases classified as severe (including amnesia, gait disturbance, somnolence and hypotension).
**Ip (2015)**	Case report of drug-induced cholestatic jaundice due to GS and CS.
**Raaijmaakers (2008)**	N/A
**vonFelden (2013)**	Intake of a preparation containing glucosamine and chondroitin sulfate resulted in a biopsy-proven acute and severe autoimmune hepatitis.<br><br>Response to steroids was favorable and resulted in complete remission of the patient. Diagnostic work-up of the case revealed no other possible cause of liver injury, and causality assessment using the Roussel Uclaf Causality Assessment Method (RUCAM) resulted in a possible causal relationship between intake of glucosamine and chondroitin sulfate and the adverse hepatic reaction.

Other Studies

First Author (Year)	Safety
**Arora (2020)**	The study demonstrates better GI tolerability of GS as compared to ibuprofen.
**Conrozier (2019)**	Tolerability was rated as excellent in 97% of patients.<br><br>Among patients with AEs (3%), 1.7% reported gastrointestinal disturbances (diarrhea, bloating, constipation, epigastric pain) and 0.5% allergic skin reactions. No severe AE was reported.
**Ganti (2018)**	N/A
**Hoffer (2001)**	N/A
**Kanzaki (2016)**	N/A
**Lehrer (2024)**	In UK Biobank subjects, glucosamine use was associated with increased corneal compensated IOP ($p = 0.002$, two-tailed t-test). This was also true in subjects without glaucoma ($p = 0.002$, two-tailed t-test). No significant association of glucosamine and IOP was detected in subjects with a diagnosis of glaucoma.<br><br>In MedWatch, 0.21% of subjects taking glucosamine reported glaucoma, 0.29% of subjects using budesonide reported glaucoma, and 0.22% of subjects using fluticasone reported glaucoma. In contrast, 0.08% of subjects using any other drug reported glaucoma. This variability is significant ($p < 0.001$, two-tailed Fisher exact test).<br><br>Data from FinnGen on risk of primary open angle glaucoma (POAG) or glaucoma in subjects using glucosamine prior to the diagnosis of the disease revealed a significantly increased risk for both POAG (hazard ratio 2.35) and glaucoma (hazard ratio 1.95).

**Martel-Pelletier (2017)**	N/A
**Peluso (2016)**	No adverse events were reported in either group.
**Persiani (2005)**	There were no safety issues during or after treatment with any of the tested doses.
**PuigdellívolGrifell (2024)**	At 3 months, one patient reported fluid retention; and at 6 months, one patient reported nausea. These AEs were considered mild and not related to the study treatment, and did not lead to patients' study withdrawal.
