# Supplementary figures and images for: The Safety and Efficacy of Glucosamine and/or Chondroitin in Humans: A Systematic Review
Source: Nutrients. 2025 Jun 24;17(13):2093. doi: 10.3390/nu17132093 (PMC12250884; doi:10.3390/nu17132093)

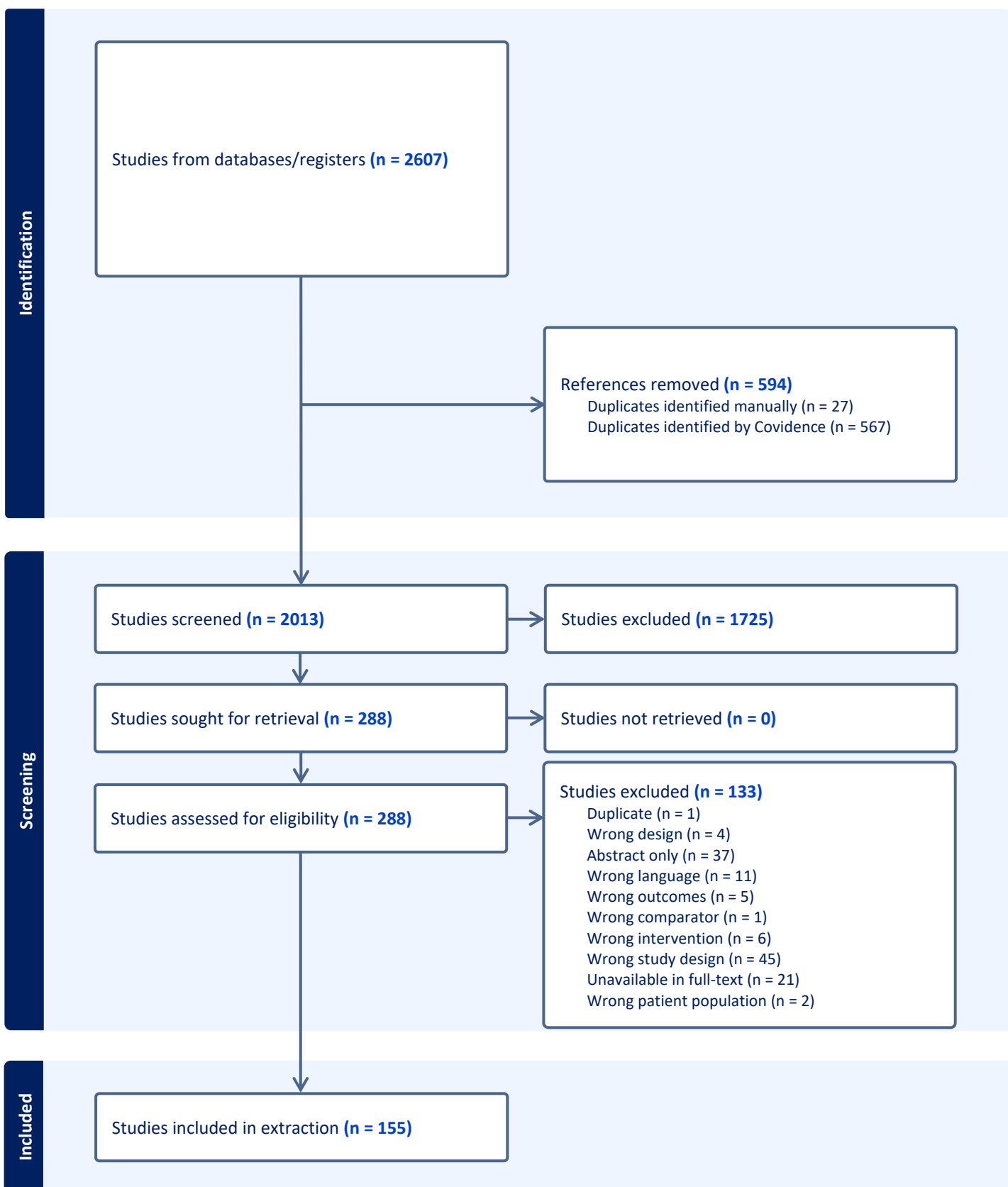

Supplement: Supplementary file 1 [file nutrients-17-02093-s001.zip › PRISMA Figure.pdf]
